# Vitamin B_2_ Catabolism: Nature’s Route from Riboflavin to Acetoacetate and Pyruvate

**DOI:** 10.1021/acscentsci.5c01234

**Published:** 2025-11-20

**Authors:** Sreyashi Sinha, Xiaohong Jian, Sanjoy Adak, Saad Naseem, Jessica L. Steiner, Dmytro Fedoseyenko, Aarthy Thiagarayaselvam, Tadhg P. Begley

**Affiliations:** Department of Chemistry, 14736 Texas A&M University, College Station, Texas 77843, United States

## Abstract

Here we report the cloning and complete *in vitro* reconstitution of the enzymes of the riboflavin catabolic pathway. The pathway begins with the oxidative removal of ribose to form lumichrome, which is then solubilized by a P450-catalyzed oxidation of the C7 methyl group, followed by hydrolytic degradation of the C-ring pyrimidine. Loss of C4, in a thiamin-dependent heterocycle decarboxylation, is followed by a xanthine oxidase and Rieske dioxygenase-mediated degradation of the quinoxaline ring. Catechol dioxygenase then catalyzes the conversion of the resulting A-ring-derived catechol to form 4-methyl-6-carboxypyrone. This is cleaved through a hydrolysis/hydration/retroaldol sequence to form pyruvate and acetoacetate, both of which are substrates for the citric acid cycle. The elucidation of the riboflavin catabolic pathway fills an important gap in our understanding of riboflavin metabolism and sets the stage for evaluating the impact of riboflavin catabolism on human and animal nutrition as well as the function of lumichrome as a quorum sensor mimic in the rhizosphere.

## Graphical abstract

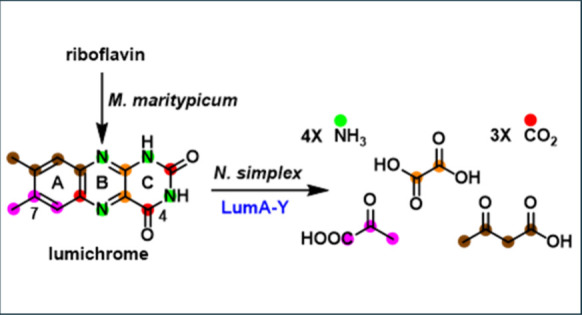

## Introduction

Riboflavin (Vitamin B_2_) is required for metabolism in all forms of life. Its discovery dates to 1879 when Blyth isolated a water-soluble component of cows’ milk whey and named it lactochrome.^1^ By the early 1900s, experiments demonstrated that a milk-derived factor was required for normal physiological development in rats. Nearly 40 years later, this factor was finally structurally characterized and named riboflavin.^2,3^ In 1939, Sebrell and Butler established that riboflavin is an essential nutrient for humans.^4^ Today, approximately 9,000 tons of riboflavin are produced annually for use in human and animal nutritional supplements.^5^ Increasingly, riboflavin production via fermentation is replacing traditional chemical synthesis methods.^6^

Riboflavin (**1**) serves as the precursor for the FAD and FMN flavin cofactors. Flavins are highly versatile cofactors,^7–15^ with flavoenzymes catalyzing a wide range of transformations, including oxidoreductases (90%), transferases (4.3%), lyases (2.9%), isomerases (1.4%), and ligases (0.4%).^16^ Flavins function as electron transfer cofactors by transitioning between oxidized, semiquinone, and reduced states. Reduced flavins are also capable of reacting with molecular oxygen to form C4a or N5 hydroperoxide, which facilitates the insertion of oxygen atoms into various metabolites.^17^ Additionally, flavins can act as hydride donor/acceptor cofactors.

Riboflavin is an essential vitamin for both animals and humans, with a recommended dietary allowance (RDA) of 1.3 mg/day.^18^ Insufficient dietary intake of riboflavin or mutations in riboflavin transport genes are the primary causes of riboflavin deficiency, which, if untreated, can lead to anemia and neurodegeneration.^19^ While the biosynthetic pathway of riboflavin has been extensively characterized in bacteria, fungi, and plants,^20–22^ its catabolism remains poorly understood. In 1958, *Pseudomonas RF* was identified as a riboflavin-catabolizing organism.^23^ Proposed intermediates in its catabolic pathway include ribityl-6,7-dimethylquinoxaline dione (**2**), 6,7-dimethylquinoxaline dione (**3**), and 3,4-dimethyl-6-carboxypyrone (**4**), as shown in Figure 1. However, none of the enzymes involved in this catabolic pathway were reconstituted, and the strain was not preserved.

**Figure 1:**
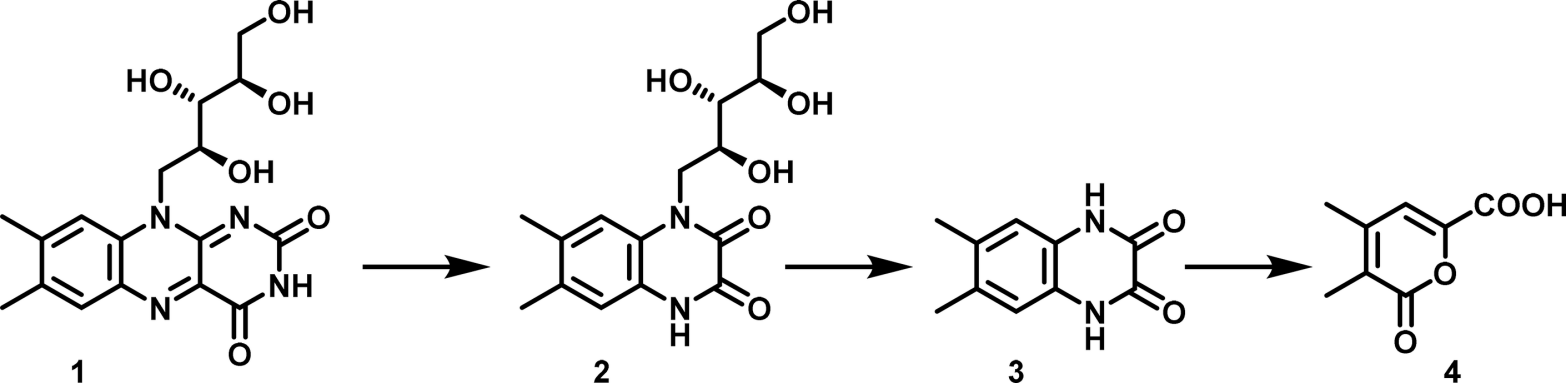
Proposed pathway for the *Pseudomonas RF*-mediated degradation of riboflavin.

Half a century later, the first riboflavin catabolic gene cluster was identified in a *Microbacterium maritypicum* G10 strain isolated by screening soil samples for bacteria able to grow on riboflavin as a carbon and nitrogen source.^24–26^ In this strain, a set of 5 genes including an FMN-dependent riboflavin monooxygenase (RcaE), catalyzes the conversion of riboflavin to ribose (**6**) and lumichrome (**5**).^24,27^ Ribose is used to sustain cell growth, and unmodified lumichrome precipitates from the growth medium (Figure 2a). Lumichrome (**5**) has been previously characterized as a riboflavin degradation product and is ubiquitous in the rhizosphere.^28^ In this study, we have identified the first bacterial lumichrome catabolic gene cluster (Figure 2b) in *Nocardioides simplex*. The corresponding enzymes were overexpressed, purified, and biochemically characterized, enabling the complete reconstitution of the riboflavin catabolic pathway.

**Figure 2:**
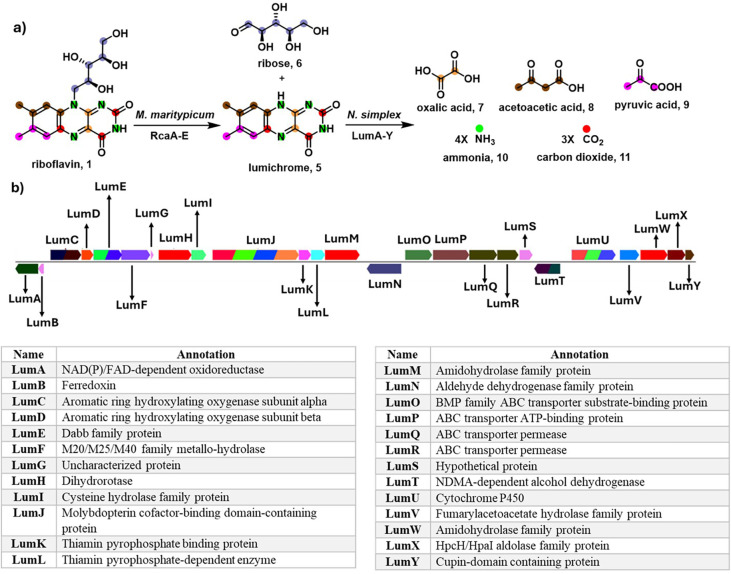
a) Outline of the bacterial riboflavin catabolic pathway as currently understood. The genes for lumichrome formation were from *Microbacterium maritypicum* G10, and those for degradation were from *Nocardioides simplex*. b) The lumichrome catabolic gene cluster and preliminary gene function assignments. The experimentally derived functional assignments and the detailed riboflavin catabolic pathway are shown in Figure 12.

## Methods and Results

### Isolation of a Lumichrome Catabolic Strain

Soil samples from various sites were cultured in minimal media supplemented with lumichrome, leading to the isolation of a strain capable of using lumichrome as its sole carbon and nitrogen source. 16S rRNA sequencing identified the strain as *Nocardioides simplex*. The genome sequence of the related *N. simplex* ATCC 6946 ^29^ was available, and we confirmed its ability to grow on lumichrome. Therefore, we used this strain for our studies. While our studies were in progress, Takaya reported the isolation of three other lumichrome-catabolizing *Nocardioides* strains,^25^ but the lumichrome catabolic operon was not identified.

### Identification of the Catabolic Operon

The catabolic operon was identified by first proposing a set of chemically plausible lumichrome catabolic pathways. Lumichrome, a highly insoluble metabolite (solubility: 4.8 mg/L, 20 μM), is readily solubilized by *N. simplex* cultures. This suggests that the early steps in lumichrome catabolism might involve lumichrome solubilization by oxidation of the methyl groups to carboxylic acids or by hydrolysis of the uracil ring. Subsequent degradation may then occur through catechol dioxygenase-mediated ring cleavage of the diaminobenzene moiety (Figure 3).

**Figure 3:**
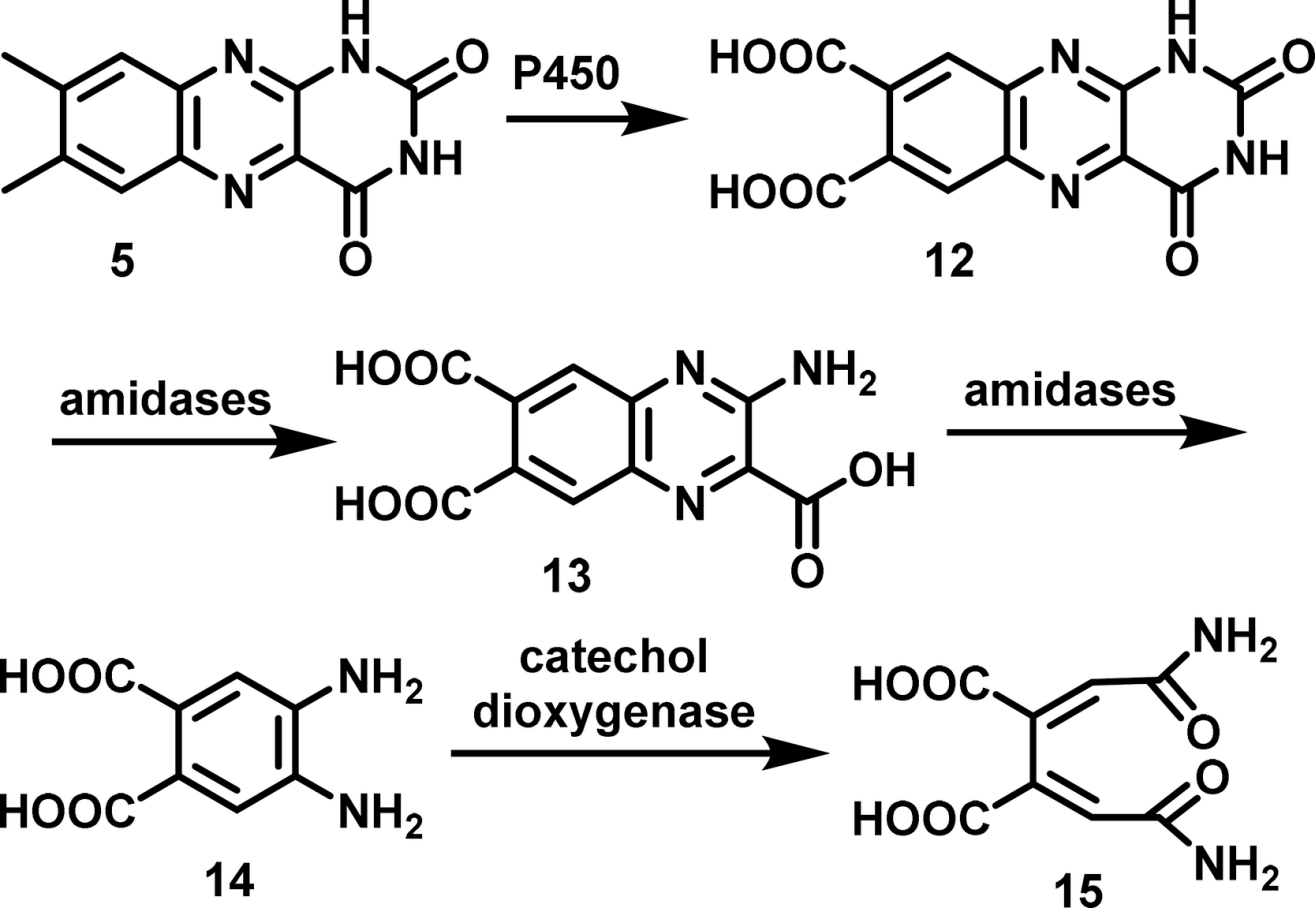
Outline of the initial hypothesis for lumichrome catabolism in *N. simplex*.

Based on this analysis, we searched the *N. simplex* genome, using the EFI sequence analysis tools,^30,31^ for gene clusters containing a P450, multiple amidases, and a catechol dioxygenase. This analysis led to the identification of a hypothetical 25-gene lumichrome catabolic operon (Figure 2b).

### Lumichrome Solubilization by Oxidation of the Aryl Methyl Group

Sequence analysis demonstrated that LumU is a single subunit P450 in which the heme, the ferredoxin, and the ferredoxin reductase domains are all encoded on a single polypeptide.^32^ Consequentially, no additional electron transfer proteins are required for catalytic activity.

LumU catalyzes the oxidation of lumichrome (**5**) to 7-carboxylumichrome (**18**) via alcohol and aldehyde intermediates (Figure 4), similar to other reported same-site multistep P450-catalyzed reactions.^33–35^ This oxidation achieves a 10-fold increase in lumichrome solubility (20 μM to 180 μM).

**Figure 4:**
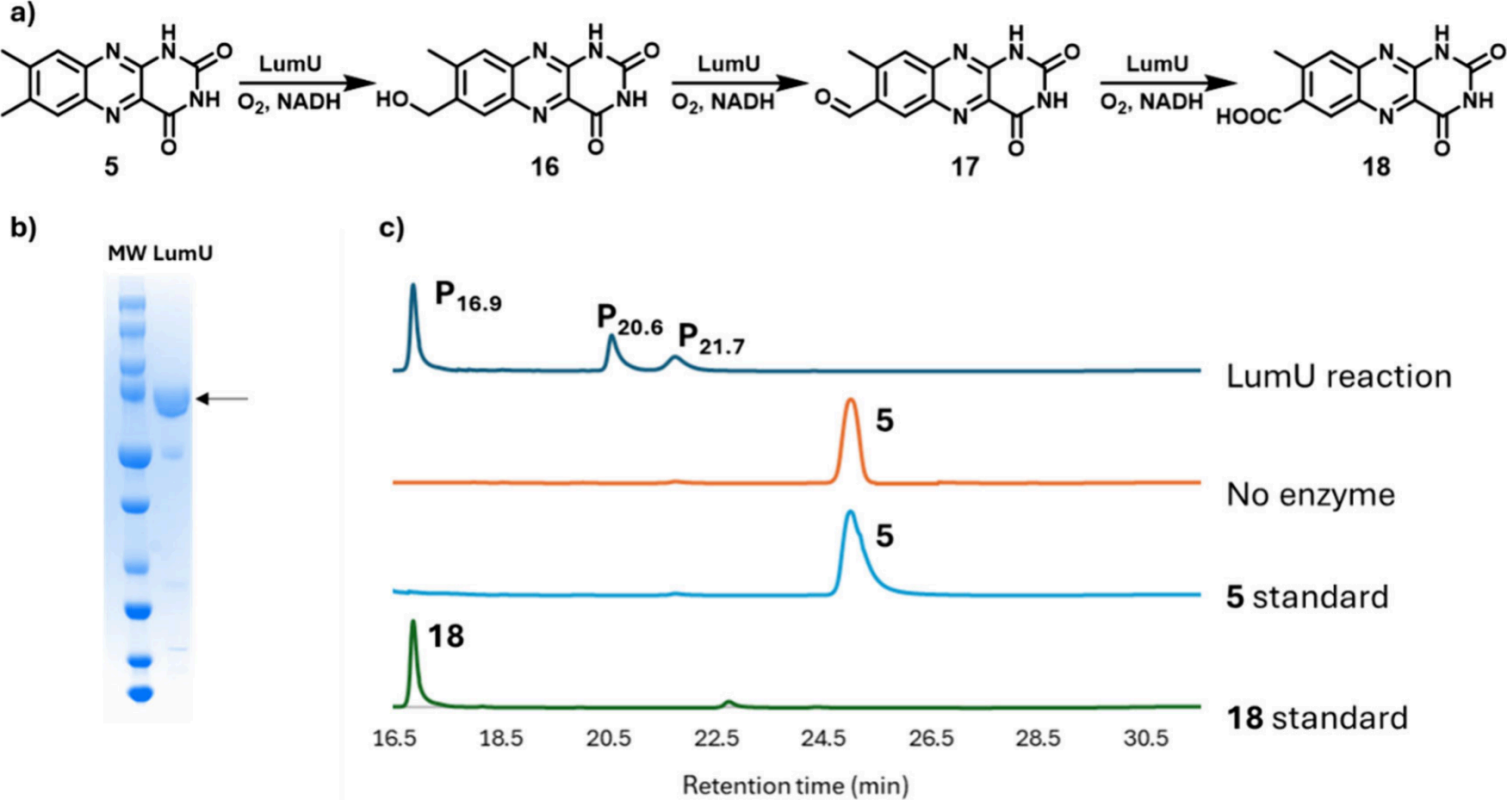
a) Reaction catalyzed by LumU. b) SDS-PAGE analysis of purified LumU (81 kDa). c) Chromatographic analysis of the LumU-catalyzed reaction (Figure S5). The final reaction product (P_16.9_) was initially identified as **18** by comigration with synthetic 7-carboxylumichrome (**18**) (Figures S1–S4) and further characterized by MS and NMR analysis (Figures S6 and S7). The intermediates P_20.6_ and P_21.7_ were characterized by MS analysis as **16** and **17**, respectively (Figure S6).

Subsequent experimentation demonstrated that LumT and LumN catalyze the oxidation of 7-hydroxymethyllumichrome (**16**) to 7-formyllumichrome (**17**) and 7-carboxylumichrome (**18**) respectively. Since LumU alone can catalyze the oxidation of lumichrome to 7-carboxylumichrome (**18**), the functions of LumT and LumN are currently unclear.

### Solubilization by Degradation of the Pyrimidine Ring

To improve the solubility of intermediates following methyl group oxidation, hydrolysis of the pyrimidine ring was proposed as the potential next step. Six putative hydrolases within the cluster (LumH, LumI, LumM, LumW, LumF and LumV) were tested individually for activity with 7-carboxylumichrome (**18**). Among these, only LumH formed a new product, which was identified as quinoxaline **19**. The LumH-catalyzed reaction is reversible, with a maximum conversion of 40% observed at equilibrium.

Treatment of compound **19** with LumI hydrolyzed the urea moiety, producing amino-quinoxaline **20**. Subsequent treatment of **20** with LumM hydrolyzed the imidamide of **20**, yielding **21** (Figure 5). The degradation of the pyrimidine ring of **18** resulted in a further 1.5-fold increase in solubility, generating an intermediate with the same solubility as riboflavin (270 μM). We also found that while lumichrome (**5**) is a substrate for LumH, the resulting product is not a substrate for LumU, demonstrating that methyl group oxidation precedes pyrimidine ring degradation (Figure S19).

**Figure 5:**
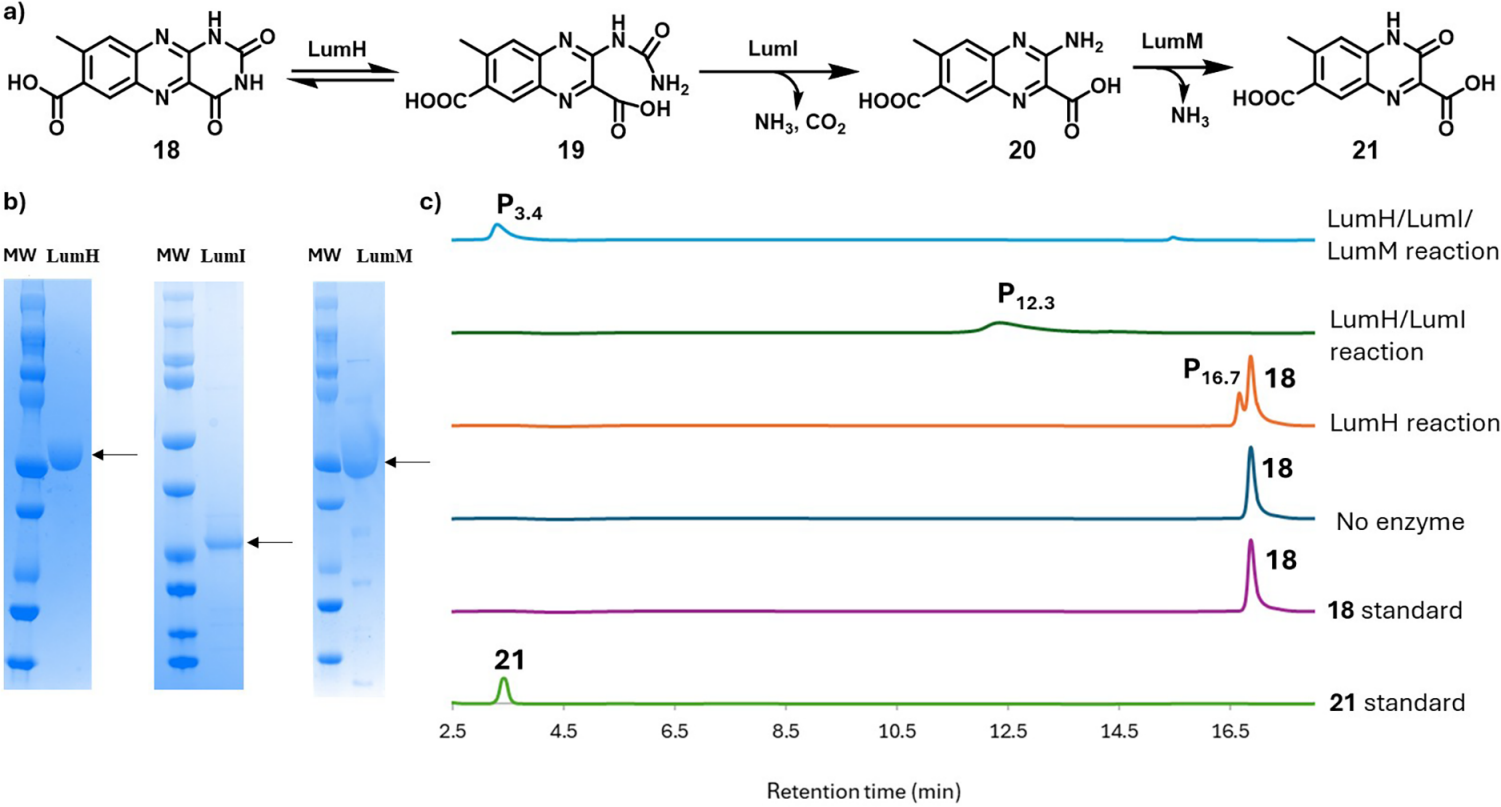
a) Reactions catalyzed by LumH, LumI, and LumM. b) SDS-PAGE analysis of purified LumH (80 kDa), LumI (24 kDa), and LumM (52 kDa). c) Chromatographic analysis of the LumH, LumI, and LumM-catalyzed reactions of 7-carboxylumichrome **18**. Product P_16.7_ was identified as **19** by LC-MS analysis. Products P_12.3_ and P_3.4_ were similarly identified as **20** and **21**, respectively, by LC-MS analysis and further characterized by NMR (Figures S8–S18, S20, and S21).

### LumK/LumL Are a Thiamin-Dependent Heterocycle Decarboxylase

LumK and LumL are both annotated as thiamin pyrophosphate binding proteins. Since the structure of quinoxaline carboxylic acid **21** is reminiscent of an α-keto acid, and the TPP-dependent decarboxylation of keto-acids is well-precedented,^36^ we considered the possibility that the LumK/LumL-catalyzed decarboxylation of **21** might be the next step in the pathway. To overcome LumK insolubility, LumK and LumL were coexpressed to give the active enzyme as a 1:1 complex. This enzyme catalyzed the decarboxylation of **21** to give **22**. (Figure 6). To the best of our knowledge, TPP-dependent heterocycle decarboxylation reactions have not been previously reported. A mechanistic proposal for this reaction is outlined in Figure 6d.

**Figure 6:**
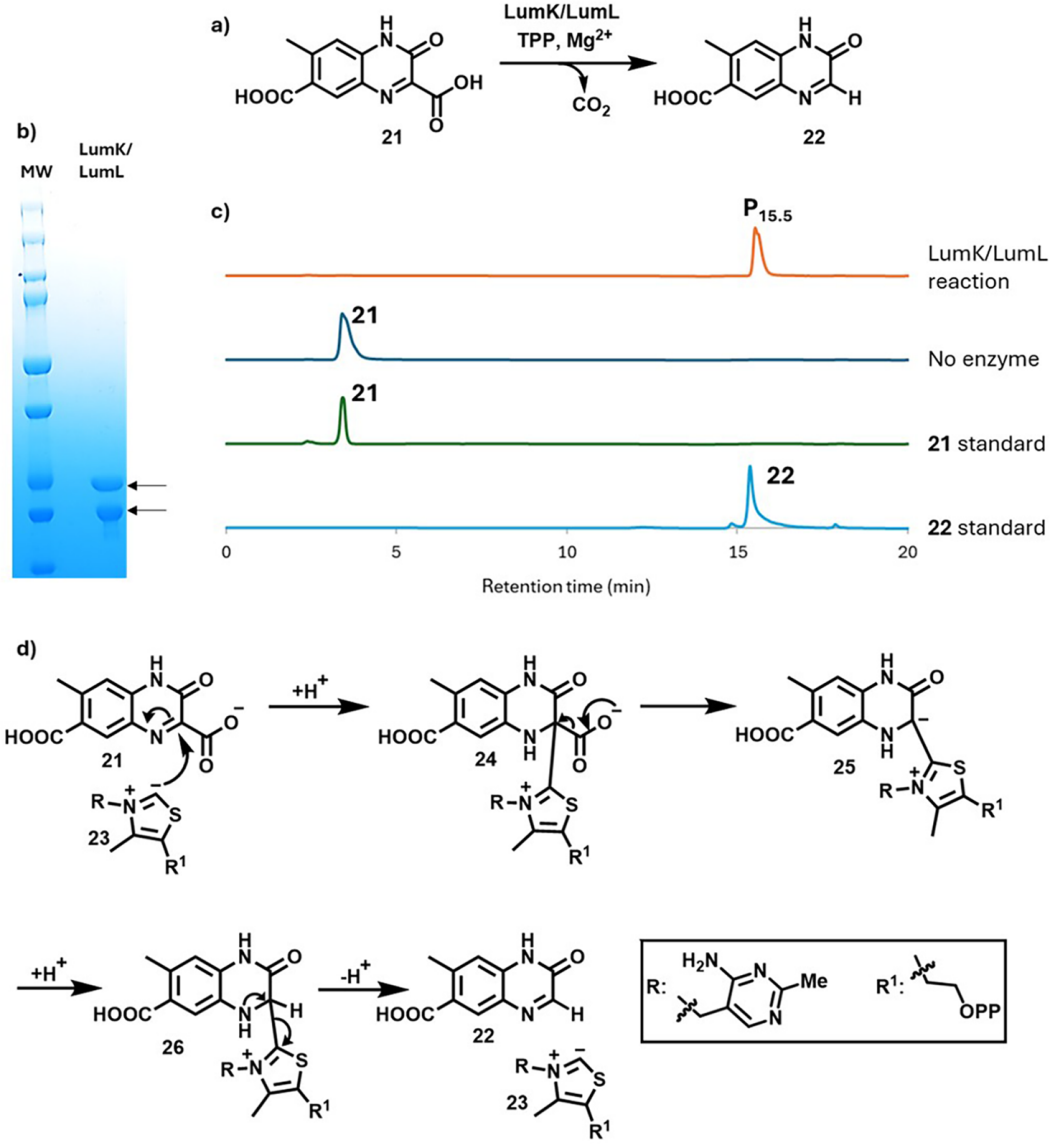
a) Reaction catalyzed by LumK/LumL. b) SDS-PAGE analysis of purified LumK (20 kDa) and LumL (23 kDa). c) Chromatographic analysis (254 nm) of the LumK/LumL-catalyzed reaction. Product P_15.5_ was identified as **22** by comigration with a synthetic standard (Figures S22–S24 and S26a) and by NMR and LC-MS (Figures S25 and S26). d) Proposed mechanism for the LumK/LumL-catalyzed reaction.

### Xanthine Oxidase (LumJ) Catalyzes the Hydroxylation of **22**

Quinoxalines are frequently found in natural products and herbicides,^37^ but their catabolism has not yet been fully elucidated. It has been reported that *Pseudomonas putida* can convert quinoxaline (**27**) to amide **28** and diol **29** (Figure 7a), but the genes involved have not been identified.^38^ Analysis of the remaining uncharacterized genes in the lumichrome catabolic operon demonstrated probable xanthine oxidase and Rieske dioxygenase encoding genes, consistent with the chemistry needed for the formation of **28** and **29**.

**Figure 7:**
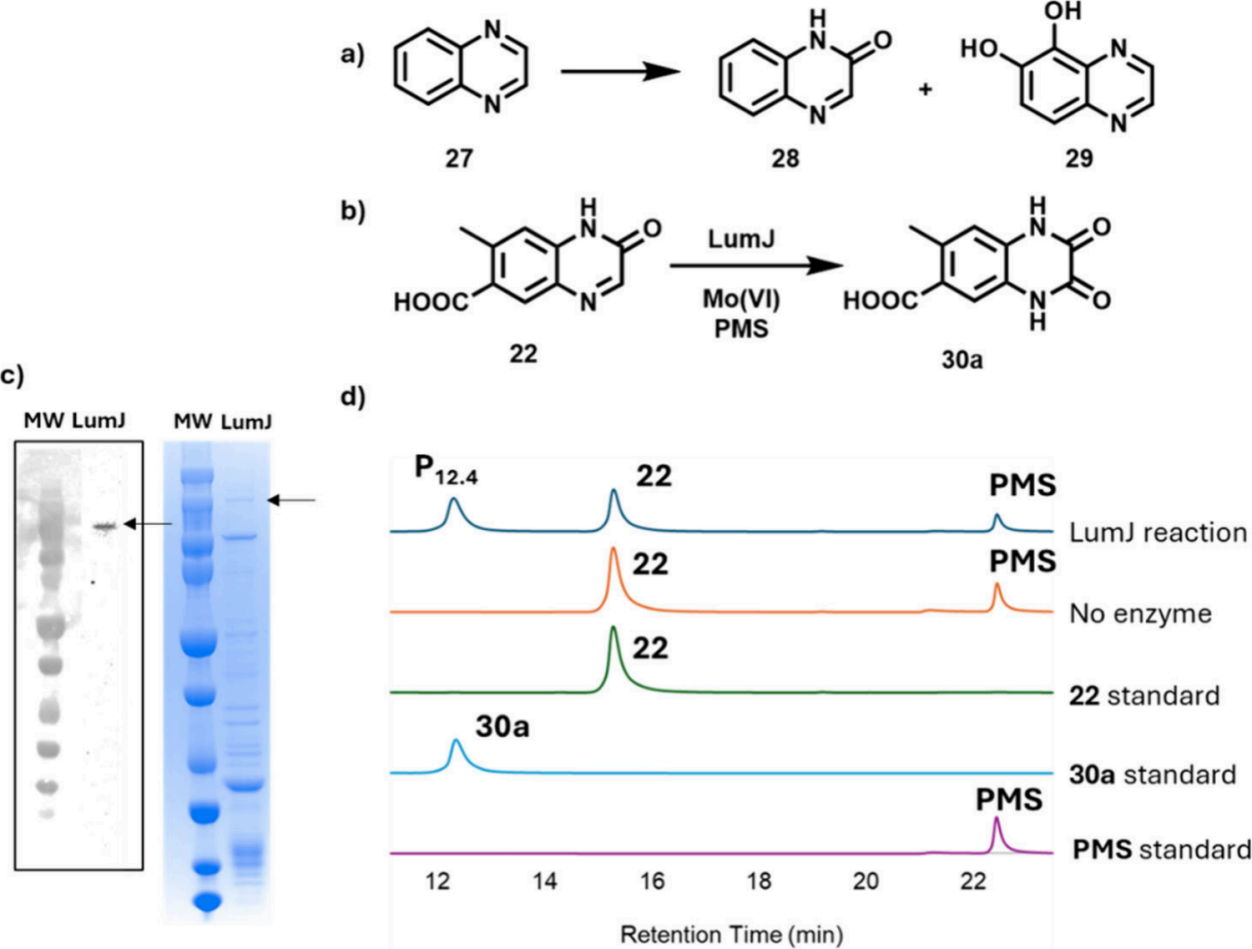
a) Quinoxaline catabolites formed by *Pseudomonas putida*. b) The LumJ-catalyzed reaction of quinoxaline **22**. c) Western blot showing LumJ overexpression (140 kDa) and corresponding SDS-PAGE analysis. d) Chromatographic analysis (340 nm) of the LumJ-catalyzed reaction. Product (P_12.4_) was identified as **30a** by comigration with a synthetic standard (Figures S27–S30) and by LC-MS and NMR analysis (Figures S31 and S32). PMS = phenazine methosulfate.

Xanthine oxidases are complex enzymes and are known to hydroxylate a variety of nitrogen-containing heterocycles.^39,40^ These enzymes typically require an N-terminal domain with two [2Fe-2S] clusters, an FAD-binding domain, and a C-terminal domain that binds the molybdopterin cofactor. Sequence analysis of LumJ indicated that it contained these predicted domains. The protein was overexpressed at low levels, purified, and incubated with compound **22**. HPLC analysis of the reaction mixture demonstrated the formation of a new product, P_12.4_, which was identified as quinoxaline dione **30a** by comigration with a synthetic standard (Figures S27–S30) and by LC-MS and NMR analysis (Figures S31 and S32).

### Rieske Dioxygenase (LumA/LumB/LumC/LumD) Catalyzes the Dihydroxylation and Ring Opening of **30a**

Sequence analysis of LumC/LumD suggested that these proteins function as the α and β subunits of a Rieske dioxygenase. These nonheme dioxygenases participate in a wide range of biological oxidative reactions and are known to catalyze the dihydroxylation of aromatic rings.^41–43^ Sequence analysis suggested that LumA and LumB encoded the ferredoxin and ferredoxin-NADP^+^ reductase^44–48^ required to mediate electron transfer from an exogenous reducing agent to the active site. All four subunits were overexpressed individually and added to the reconstitution reaction mixture. We first tested compound **22** as a substrate for LumA/LumB/LumC/LumD, but no new product was detected. We then tested compound **30a** (Figure 8). HPLC analysis demonstrated the formation of a new product (P_13_) with mass and NMR spectra consistent with structures **33a** or **34a** (Figures S48–S50). Synthetic samples of **33a** (Figures S38–S47) and **33b** (Figures S33–S36) comigrated with the enzymatic products generated from **30a** and **30b** respectively (Figures 8c and S51–S54).

**Figure 8:**
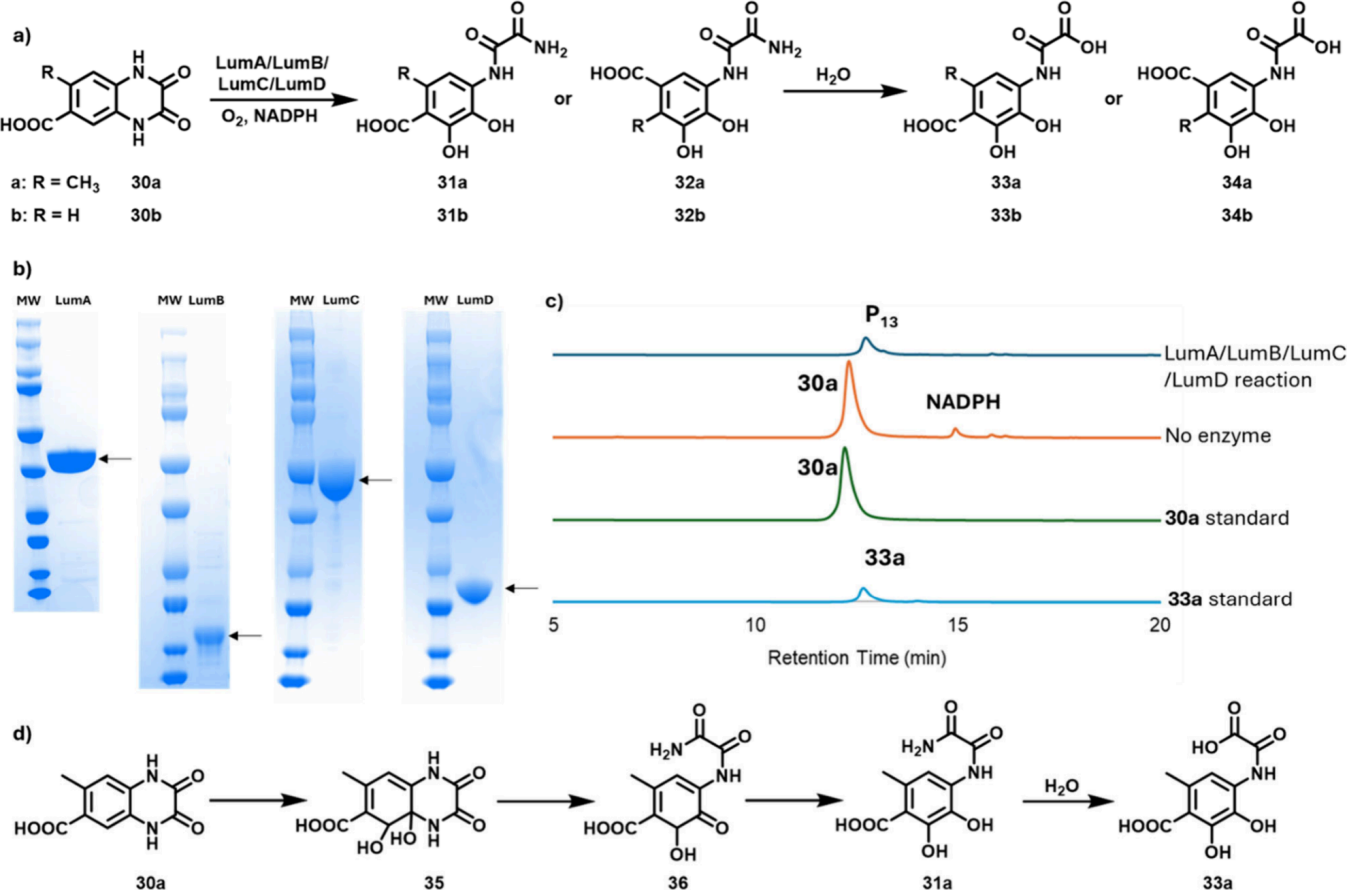
a) The LumA/LumB/LumC/LumD-catalyzed the conversion of **30a** to P_13_. R = CH_3_ for **30a**–**34a**, and R = H for **30b**–**34b**. b) SDS-PAGE analysis of purified LumA (38 kDa), LumB (14 kDa), LumC (49 kDa), and LumD (23 kDa). c) Chromatographic analysis (340 nm) (Figure S49) of the LumA/LumB/LumC/LumD-catalyzed reaction. Product (P_13_) was identified as **33a** by retention time relative to a synthetic standard (Figures S38–S47) and by LC-MS and NMR analysis (Figures S48 and S50). d) Mechanistic proposal for the Rieske dioxygenase LumA/LumB/LumC/LumD-catalyzed oxidation of **30a** to **33a**.

The regiochemistry of the reaction product was confirmed by NMR analysis of the product generated from **30b** where the ortho coupling constants of the aromatic protons unequivocally identified the reaction product as **33b** (Figures S53 and S55). A mechanism for the formation of **33a** is shown in Figure 8d.

The facile hydrolysis of amides **31a** and **31b** was unanticipated. Synthesized **31b** in reaction buffer showed similar amide lability, demonstrating that this hydrolysis is nonenzymatic (Figure S37).

### LumE is an Extradiol Catechol Dioxygenase

Sequence analysis of LumE demonstrated low similarity (30%) with catechol dioxygenase, a nonheme Fe-dependent dioxygenase that cleaves 1,2-dihydroxy-substituted benzene rings.^49–53^ Since the Rieske dioxygenase product is a catechol, we considered the possibility that LumE might catalyze the oxidative ring opening of **33a**. Treatment of **33a** with LumE resulted in the formation of a new product (P_17.5_) identified as pyrone **37** (Figure 9). A mechanistic proposal for the formation of **37** is shown in Figure 9d. The yield of the pyrone was sensitive to the substituents on the aromatic ring (Figures S61–S65).

**Figure 9:**
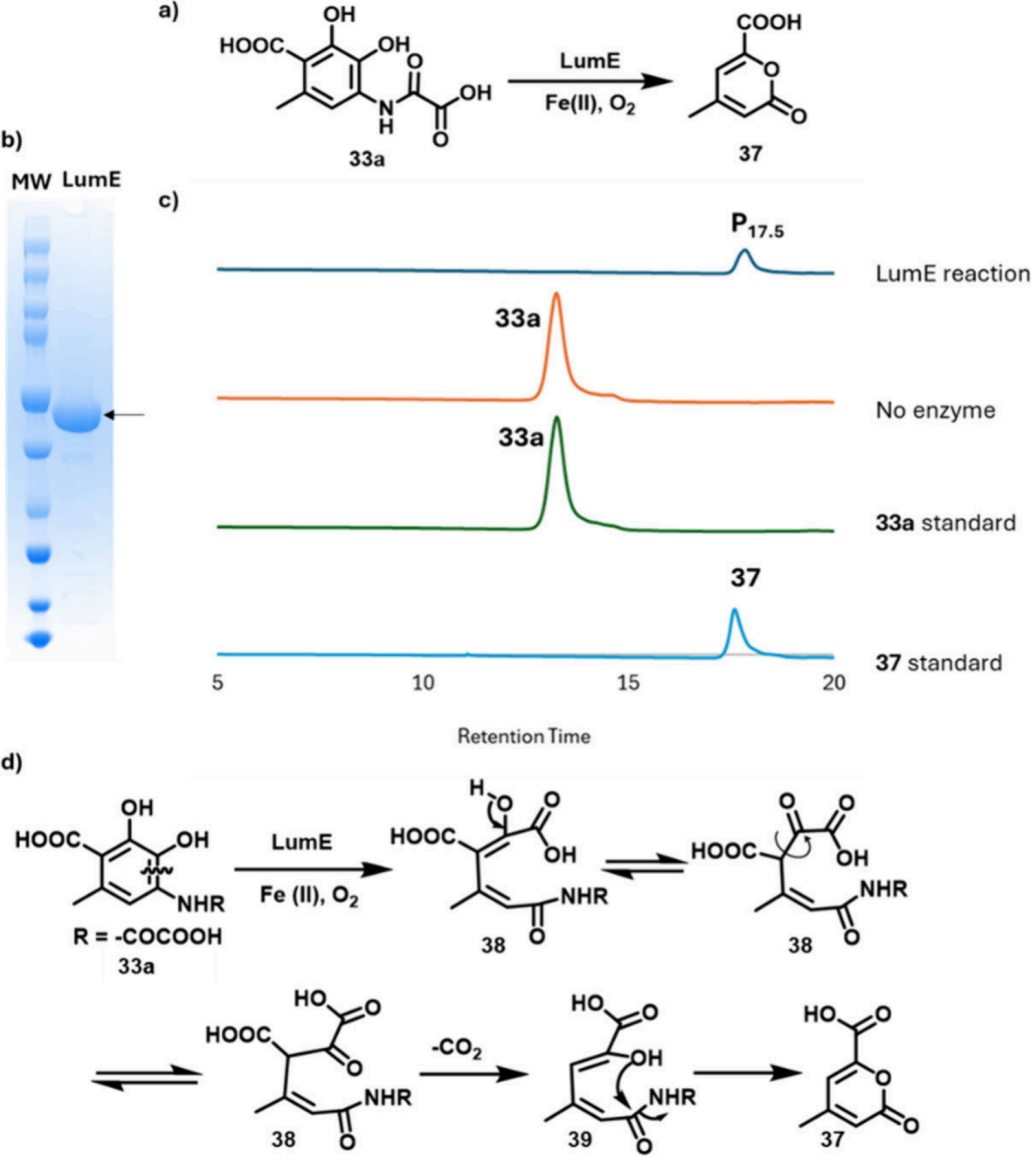
a) The LumE-catalyzed reaction. b) SDS-PAGE analysis of purified LumE (46 kDa). c) Chromatographic analysis (280 nm) of the LumE-catalyzed reaction. Product (P_17.5_) was identified as **37** by comigration with a synthetic standard (Figures S56–S58) and by LC-MS and NMR analysis (Figures S59 and S60). d) Proposed mechanism for the LumE-catalyzed oxidation of catechol **33a** to pyrone **37**.

### Conversion of Pyrone **37** to Pyruvate (**9**) and Acetoacetate (**8**)

Catechol dioxygenase-catalyzed reactions have been extensively studied and the conversion of the enzymatic products to simpler metabolites has been characterized in numerous systems.^54–61^ Based on this, we proposed that hydrolytic ring opening of pyrone **37** followed by conjugate addition of water and a retroaldol reaction would give pyruvate (**9**) and acetoacetate (**8**) as the final products of lumichrome catabolism (Figure 10a, Figure 11a). While LumX was a likely candidate for the aldolase, the lactonase and hydratase involved could not be reliably identified by sequence analysis. We therefore assayed all of the remaining putative hydrolases (LumY, LumW, LumF, and LumV) for the proposed lactonase and hydratase activities.

**Figure 10:**
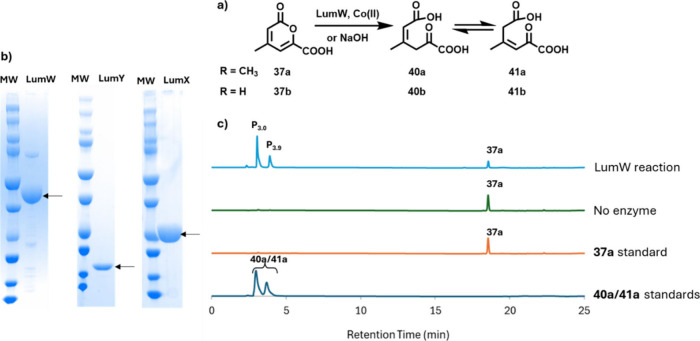
a) Reaction catalyzed by LumW. b) SDS-PAGE analysis of purified LumW (40 kDa), LumY (16 kDa), and LumX (26 kDa). c) Chromatographic analysis (254 nm) of the LumW-catalyzed reaction. The LumW products (P_3.0_ and P_3.9_) were identified by comigration with synthetic standards (Figures S66, S67, and S69) and confirmed by LC-MS and NMR analysis (Figures 11 and S68). Studies with **37b** are described in the next section (Figures 11, S70, and S71).

**Figure 11:**
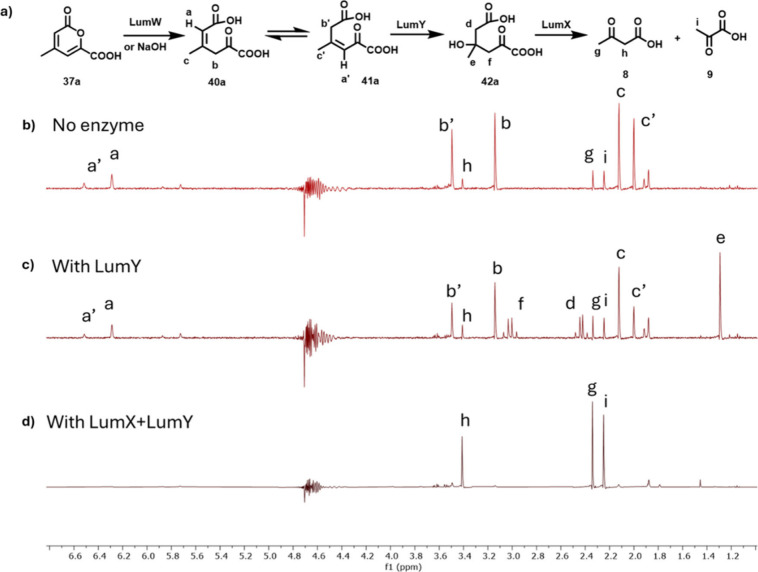
a) The LumY and LumX-catalyzed reactions. b) NMR of the pyrone hydrolysis reaction mixture showing the formation of **40a** and **41a** and a small quantity of pyruvate (**9**) and acetoacetate (**8**). c) NMR of the pyrone hydrolysis reaction mixture after treatment with LumY showing the formation of **42a** (40% conversion). d) NMR of the pyrone hydrolysis reaction mixture after treatment with LumY and LumX showing the consumption of **40a**, **41a**, and **42a** and the formation of pyruvate (**9**) and acetoacetate (**8**).

LumW is a cobalt­(II)-dependent hydrolase and catalyzes the reversible ring-opening of pyrone **37** to give a mixture of **40** and **41** (Figure 10). While rare, cobalt­(II)-dependent hydrolases have been previously identified.^62–68^ LumY was annotated as a cupin domain-containing enzyme. These proteins catalyze a diverse set of reactions.^69^ The treatment of the LumW reaction mixture with LumY demonstrated consumption of the LumW products (Figure 11). The LumY reaction product **42a**, did not absorb at 254 nm and was identified by ^1^H NMR analysis (Figure 11). A better-quality spectrum of intermediate **42b**, was obtained by running the LumW/LumY reaction using **37b** in which the absence of the methyl group retarded the rate of the retroaldol reaction (Figures S70 and S71). The identification of the function of LumX completed the characterization of the lumichrome catabolic pathway in which lumichrome is converted to acetoacetate and pyruvate, both intermediates for the citric acid cycle.

## Discussion

Although the biosynthesis of cofactors has been extensively studied, their catabolism remains largely unexplored.^70^ To date, only the catabolic pathways of pyridoxal phosphate (vitamin B_6_),^71^ NAD^+^,^72–76^ and riboflavin (current paper) have been well-characterized. This paucity of studies on vitamin B catabolism is a significant gap in our understanding of vitamin metabolism, leaving unresolved the potential impact of cofactor catabolism in the digestive tract on vitamin requirements in human and animal nutrition.

We identified two soil bacterial strains that, together, convert riboflavin to pyruvate and acetoacetate through enrichment culturing^77^ with riboflavin and lumichrome as carbon sources. *M. maritypicum* catalyzes the conversion of riboflavin to ribose and lumichrome, while *N. simplex* further degrades lumichrome into acetoacetate and pyruvate. The relevant *M. maritypicum* genes were identified by screening a cosmid library,^24^ whereas the *N. simplex* genes were identified from the genome sequence of a closely related *N. simplex* strain (ATCC 6946)^29^ by searching for gene clusters corresponding to plausible lumichrome catabolic pathways.

Here, we report the first complete *in vitro* reconstitution of the riboflavin (**1**) catabolic pathway (Figure 12). In *M. maritypicum*, the ribityl chain is oxidatively cleaved from the isoalloxazine heterocycle by riboflavin monooxygenase (RcaE)^24^ to form lumichrome (**5**),^24,26,27^ which precipitates unaltered from the culture medium. Riboflavin monooxygenase catalyzes a novel flavoenzyme-mediated transformation in which superoxide radical abstracts a hydrogen atom from C1′ of the ribose rather than recombining with the flavin to form flavin hydroperoxide as generally observed.^78^ Lumichrome catabolism in *N. simplex* begins with a solubilizing reaction in which LumU, a single subunit P450,^32^ catalyzes the oxidation of the 7-methyl group to produce 7-carboxylumichrome (**18**). This reaction contrasts with the well-characterized oxidation of the C-8 methyl group, which is initiated by methyl group deprotonation.^79,80^ This oxidation increases the solubility of lumichrome by a factor of 10. Next, the pyrimidine ring is hydrolytically degraded by LumH, LumI, and LumM to form **21**. A TPP-dependent quinoxaline decarboxylase (LumK, LumL) catalyzes the formation of **22**. This decarboxylation of a heterocyclic carboxylic acid is a new motif in TPP chemistry. In addition, two-subunit TPP-dependent decarboxylases are rare and have been previously identified only on the biosynthesis of rhizoticin^81^ and Coenzyme M.^82^ Quinoxaline **22** is then further degraded through three different oxidation reactions. First, quinoxaline oxidase (LumJ), a xanthine oxidase-like enzyme, catalyzes the formation of **30a**. This product then undergoes a ring-opening reaction catalyzed by quinoxaline dioxygenase (LumA, LumB, LumC, and LumD), a Rieske dioxygenase-like enzyme, and finally, the resulting highly functionalized benzene ring **33a** undergoes an oxidative ring-opening reaction to yield **37a** in a reaction catalyzed by catechol dioxygenase (LumE). Pyrone hydrolase (LumW) catalyzes the ring-opening of **37a**. The addition of water to **40a** and **41a**, catalyzed by dienedioate hydratase (LumY), produces **42**, which is then converted to pyruvate (**9**) and acetoacetate (**8**), both substrates for the citric acid cycle, by a retro aldolase (LumX).

**Figure 12:**
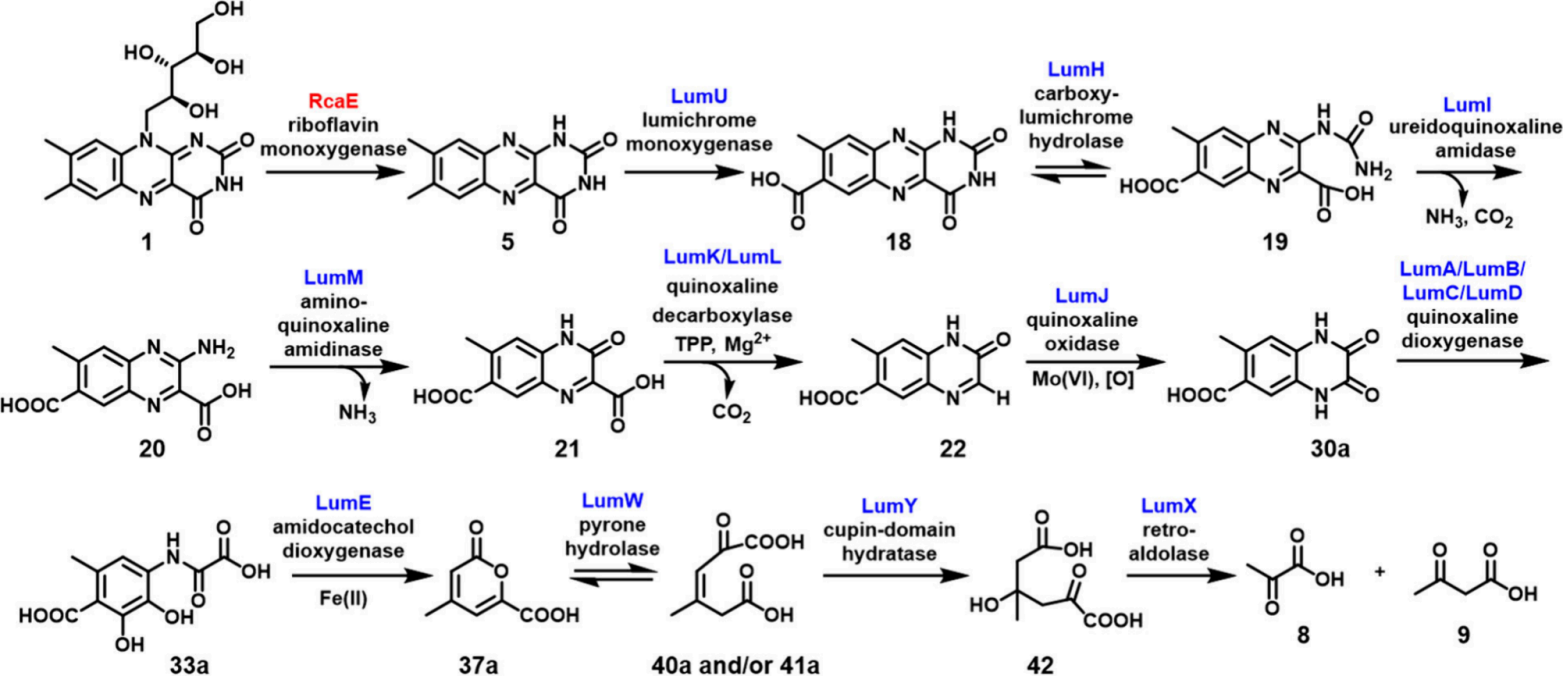
Proposed riboflavin catabolic pathway. Red denotes enzymes in *M. maritypicum*, while blue denotes enzymes in *N. simplex*.

In addition to the genes depicted in Figure 12, the lumichrome catabolic cluster includes eight other genes of unassigned function. These include four putative transport-related genes (*lumOPQR*), which may facilitate the uptake or export of pathway intermediates, a putative metallohydrolase (*lumF*), and a putative fumarylacetoacetate hydrolase (*lumV*). Two genes of unknown function (*lumG* and *lumS*) were not conserved in other *Nocardioides* strains containing the catabolic operon. In addition, the functions of the two oxidases that also catalyze the conversion of hydroxymethyl lumichrome (**16**) to 7-carboxylumichrome **18** (LumN, LumT) remain unclear, as lumichrome monooxygenase (LumU) also catalyzes these transformations. The two unassigned enzyme-encoding genes have been overexpressed (LumF and LumV). However, their inclusion in all the defined enzymatic assays described above did not reveal any essential role for these enzymes in converting lumichrome to pyruvate and acetoacetate.

*N. simplex* ATCC 6946 ^29^ was grown in defined minimal media using lumichrome as the sole carbon and nitrogen source. From metabolomic analysis using LC-MS, intermediates **19**, **20**, **30a**, and **37a** (Figure 12) were identified in the culture (Figure S72) providing further evidence for our proposed pathway.

Previous studies found evidence for three different microbial riboflavin catabolic pathways (Figure 13). None of the enzymes were purified, and all experiments were conducted using either whole cells or crude cell extracts. Pathway 1, in which the N-glycosyl bond of riboflavin is cleaved to give lumichrome (**5**) and ribitol (**43**),^83^ is similar to the *M. maritypicum* pathway.^24^ Path 2 results from the fragmentation of the C2–C3 bond of ribose and was not found in our screening.^84^ Path 3 was found by Stadtman in *Pseudomonas RF*.^85^ In this pathway, the C-ring of riboflavin is first degraded to give **2**. Removal of the ribose (**6**) then gives quinoxaline **3**, which is degraded to give pyrone **4**. This pathway is similar to the *N. simplex* pathway, except the methyl group oxidation is missing, the structure of the pyrone **4** is different from the pyrone **37a**, the ribose is removed after quinoxaline dione formation, and the formation of urea and oxalamide products. These pathway differences suggest that variants of the *N. simplex* pathway shown in Figure 12 may exist.

**Figure 13:**
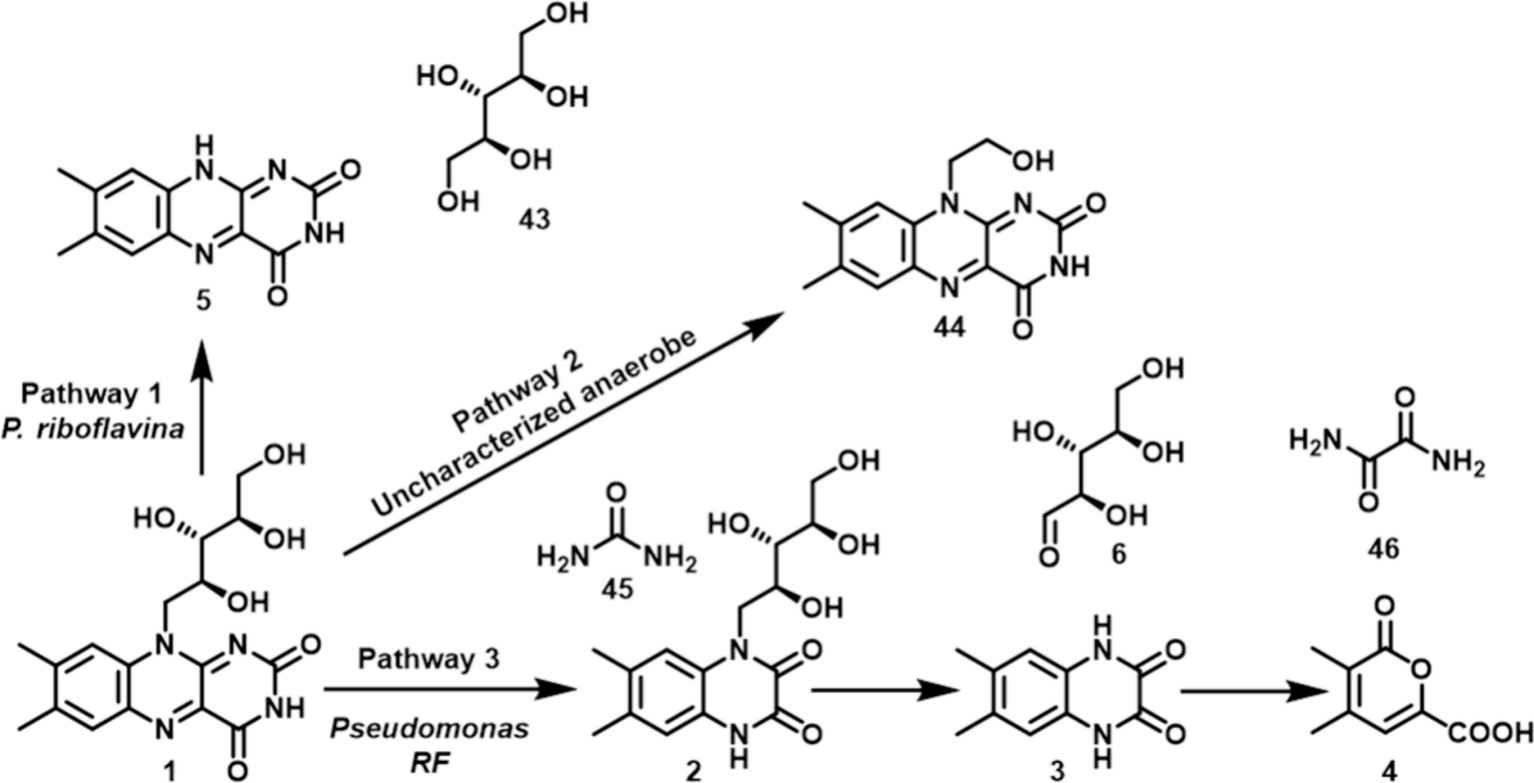
Outline of three previously discovered microbial riboflavin catabolic pathways.

Quinoxaline heterocycles are widely distributed in the biosphere.^86^ Several drugs, such as quinacillin,^87^ sulfaquinoxaline,^88^ clofazimine,^89^ and echinomycin,^90^ contain quinoxaline heterocycles. Numerous quinoxalines are used in farming as pesticides, herbicides, fungicides, and insecticides.^37,91^ For example, quinalphos is a widely used insecticide in Indian agriculture,^92,93^ with 1,200 t used in just 2011.^94^ Quinalphos is highly toxic to humans,^95^ bees,^96^ and aquatic life.^97^ The riboflavin catabolic pathway presents the first detailed study of quinoxaline catabolism, suggesting that xanthine oxidase, Rieske dioxygenase, and catechol dioxygenase-mediated chemistry may play a central role in the degradation of environmental quinoxalines.

Previous studies have demonstrated that lumichrome is produced by bacteria in the rhizosphere, where it functions as a signaling molecule that regulates various aspects of plant development, including root, stem, and leaf growth, as well as biomass accumulation.^98,99^ In addition, lumichrome functions as a quorum-sensing mimic by activating the LasR receptor in *Pseudomonas aeruginosa*.^100^ Lumichrome has also been detected in human feces,^101–104^ and lumichrome-producing bacteria have been isolated from the gut microbiome,^105^ although none of these strains have yet been identified. These findings suggest that lumichrome may also function as a quorum-sensing mimic, potentially involved in regulating gut microbiome homeostasis.

Genomic analysis (Figure S73) revealed that the two known riboflavin catabolic gene clusters are found exclusively in soil bacteria. However, no strain has been identified in which the riboflavin monooxygenase gene (RcaE) is colocalized with the genes required for lumichrome catabolism. This observation raises the possibility that the primary biological function of the initial phase of riboflavin catabolism is the biosynthesis of lumichrome as a signaling molecule, while the second phase may constitute a pathway for signal degradation. The identification of the riboflavin catabolic genes and intermediates reported in this paper provides the basic science for the investigation of these and other nonenzymatic functions of riboflavin.

## Supplementary Material



## Notes

Detailed procedures for the overexpression and purification of proteins, synthesis of all substrates and products, enzymatic reaction conditions, and HPLC, NMR, and ESI-MS analysis of enzymatic reactions (PDF)
