# Supplementary material for: Vitamin B2 Catabolism: Nature’s Route from Riboflavin to Acetoacetate and Pyruvate
Source: ACS Cent Sci. 2025 Nov 20;11(12):2353–65. doi: 10.1021/acscentsci.5c01234 (PMC12746152; doi:10.1021/acscentsci.5c01234)
Supplement: Supplementary file 1 [file oc5c01234_si_001.pdf]

Supporting Information

Vitamin B_2 Catabolism: Nature's Route from Riboflavin to Acetoacetate and Pyruvate

Sreyashi Sinha, Xiaohong Jian, Sanjoy Adak, Saad Naseem, Jessica L. Steiner, Dmytro Fedoseyenko, Aarthy Thiagarayaselvam, and Tadhg P. Begley*

Corresponding author email: begley@chem.tamu.edu

Department of Chemistry, Texas A&M University, College Station, Texas 77843, United States.

Table of Contents

Materials....................S2

Synthesis of 7-carboxylumichrome (18)....................S3

Characterization of LumU....................S5

Synthesis of 6-methyl-3-oxo-3,4-dihydroquinoxaline-2,7-dicarboxylic acid (21) and 3-amino-6-methylquinoxaline-2,7-dicarboxylic acid (20)....................S9

Characterization of LumH, LumI, and LumM....................S14

Synthesis of 7-methyl-2-oxo-1,2-dihydroquinoxaline-6-carboxylic acid (22)....................S20

Characterization of LumK and LumL....................S22

Synthesis of 7-methyl-2,3-dioxo-1,2,3,4-tetrahydroquinoxaline-6-carboxylic acid 30a....................S26

Characterization of LumJ....................S28

Synthesis of 4-(carboxyformamido)-2,3-dihydroxybenzoic acid (33b)....................S31

Synthesis of 4-(carboxyformamido)-2,3-dihydroxy-6-methylbenzoic acid (33a)....................S34

Characterization of LumA, LumB, LumC, and LumD....................S43

Synthesis of 4-methyl-2-oxo-2H-pyran-6-carboxylic acid (37a)....................S52

Characterization of LumE....................S53

Synthesis of (Z)-3-methyl-5-oxohex-2-enedioic acid (40) and (Z)-4-methyl-2-oxohex-3-enedioic acid (41)....................S60

Characterization of LumW....................S61

Characterization of LumX and LumY....................S63

Culture metabolite analysis for lumichrome catabolic intermediates....................S66

References....................S68

Materials

All chemicals were obtained from Milipore-Sigma unless specified. LB broth (Lennox formulation) was from IBI Scientific. Kanamycin was purchased from Teknova and IPTG sourced from Gold Biotechnology Inc. HPLC and LC-MS solvents were purchased from EMD and VWR and used without further purification. His trap columns Ni-affinity columns (5 mL) were bought from GE Healthcare. Econo-Pac 10DG and bio-spin 6 desalting columns were from Bio-Rad laboratories. Bacterial cultures were grown and overexpressed using baffled ultra-yield flasks from Thomson Instrument Company. 2,3-dioxo-1,2,3,4-tetrahydroquinoxaline-6-carboxylic acid (**30b**, Figure 8a) was purchased from Ambeed. 2-oxo-2H-pyran-6-carboxylic acid (**37b**, Figure S61) was obtained from Sigma-Aldrich. NMR tubes (3 mm and 5 mm diameter) used were from Wilmad Labglass. NMR solvents, D_2O, Methanol-d_4 and DMSO-d_6 were from Cambridge Isotope Laboratories Inc.

An Agilent 1200 or 1260 HPLC equipped with a quaternary pump and a diode array UV-Vis detector was used. Analysis was performed using ZORBAX Eclipse XDB-C18 column (15 cm x 4.6 mm, 5 µm particles, Agilent Technologies) or Pursuit XRs column (100 Å C18, 4.6 x 250 mm, 5 µm, Agilent technologies). Data was processed using ChemStation ver. B.04.01 SP1 (Agilent technologies).

For LumJ overexpression, PCR amplifications were performed using a C1000 automatic thermocycler (Bio-Rad) and Integrated DNA Technologies synthesized oligonucleotide primers. Phusion DNA polymerase, Gibson Assembly kit, restriction enzymes, and T4 DNA ligase were obtained from New England BioLabs. The cloning vector was purchased from Novagen. pMAL-c2E-TEV vector was provided by Dr. James Sacchettini (Texas A&M University). Plasmid mini-prep and PCR purification kits were acquired from Qiagen. DNA fragments were purified from agarose gels with the QIAquick Gel extraction kit from Qiagen. DNA sequencing was performed by Eton biosciences. *E.coli* TP1000 cells were obtained from Dr. Russ Hille, UC Riverside. *E. coli* DH5α was used as recipients for transformations during plasmid construction as well as for plasmid propagation and storage. Phenazine methosulfate (PMS), the oxidizing agent for the LumJ reaction, was obtained from Sigma-Aldrich.

Synthesis of 7-carboxylumichrome (18)

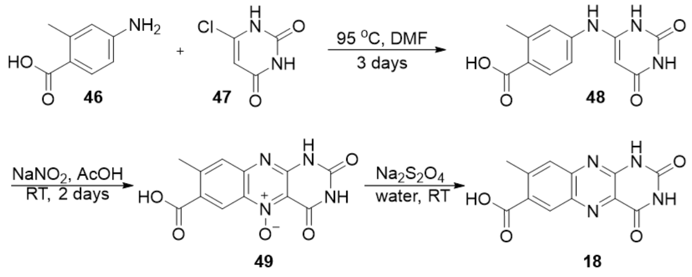

Figure S1. Synthetic scheme for 7-carboxylumichrome (**18**).

4-((2,6-dioxo-1,2,3,6-tetrahydropyrimidin-4-yl)amino)-2-methylbenzoic acid (48)

4-amino-2-methylbenzoic acid, (**46**) (3.15 g, 20.85 mmol, 3 eq) and chloro-uracil (**47**) (1.01 g, 6.95 mmol, 1 eq) were dissolved in 10 mL DMF and stirred at 95 °C for three days. This was cooled in an ice bath and the precipitated product (**48**) was filtered out, washed with water, methanol and dried *in vacuo*. The precipitate was pure and was used without further purification. Yield: 30% ^{1}H NMR (400 MHz, DMSO-d_6) δ 10.56 (s, 1H), 10.28 (s, 1H), 8.62 (s, 1H), 7.87 (d, 1H), 7.09 (d, 2H), 4.95 (s, 1H) 2.52 (s, 3H).

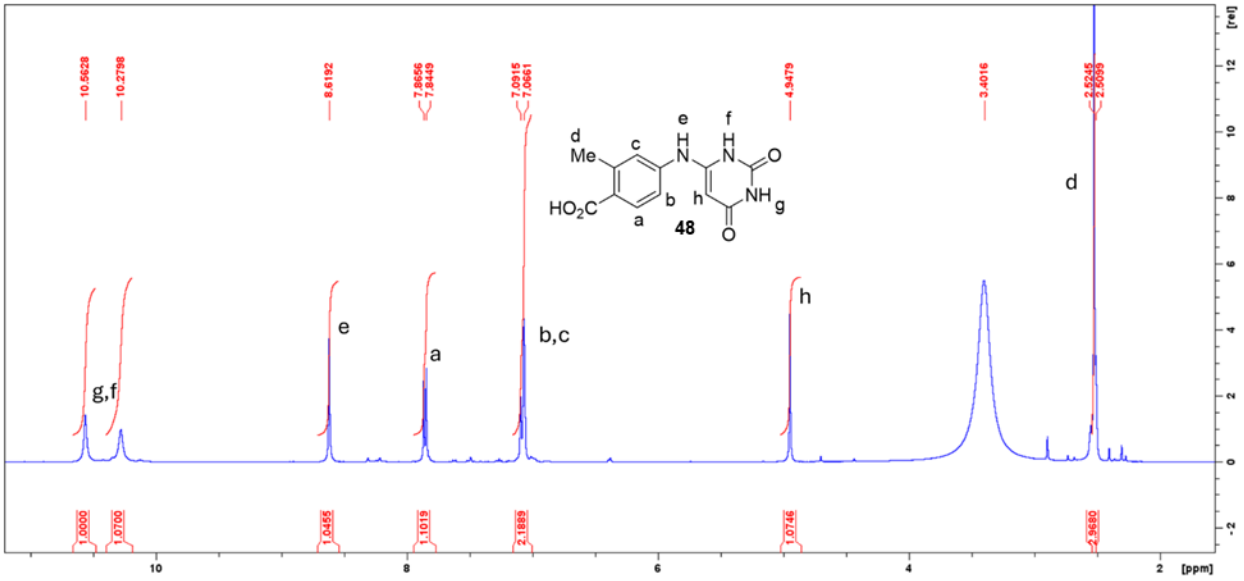

Figure S2. ^{1}H NMR of intermediate **48**.

7-carboxy-8-methyl-2,4-dioxo-1,2,3,4-tetrahydrobenzo[g]pteridine 5-oxide (49)

Compound **48** (0.5 mg, 1.92 mmol, 1 eq) was crushed and dissolved in 10 mL cold acetic acid and stirred at room temperature in the dark with $NaNO_2$ (660 mg, 9.7 mmol, 5 eq) for two days. Suspension was filtered. Solid yellow product was washed with 3 mL of acetic acid and then with 3 mL of methanol and dried at 65 °C. Oxidation on air exposure led to the formation of **49**. This was used for the next reaction without further purification. ^{1}H NMR (400 MHz, DMSO-d_6) δ 11.94 (s, 1H), 11.49 (s, 1H), 8.69 (s, 1H), 7.68 (s, 1H), 2.67 (s, 3H).

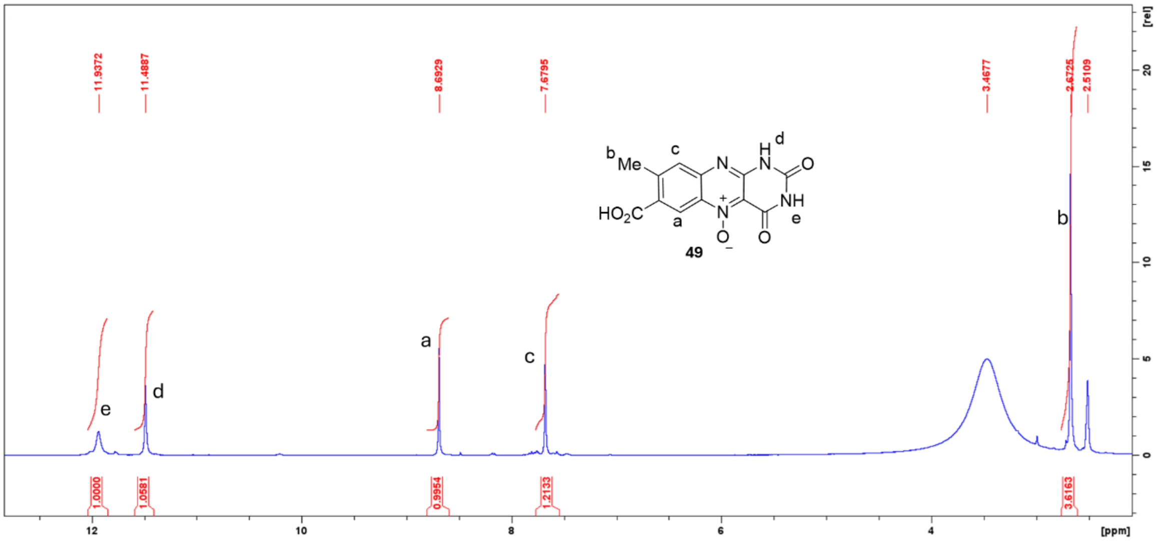

Figure S3. ^{1}H NMR of intermediate **49**.

7-carboxylumichrome (18)

Compound **49** (0.50 g, 1.72 mmol, 1 eq) was heated with sodium dithionite (1.198 g, 6.88 mmol, 4 eq) and stirred in a sealed tube at 110 °C 30 min. After cooling, the suspension was filtered out, washed with water and dried. Then, oxidation by air exposure provided the final product, **18**, as a yellow solid. $[M-H]^-$: 271.0474. ^{1}H NMR (400 MHz, DMSO-d_6) δ 12.02 (s, 1H), 11.76 (s, 1H), 8.52 (s, 1H), 7.80 (s, 1H), 2.74 (s, 3H).

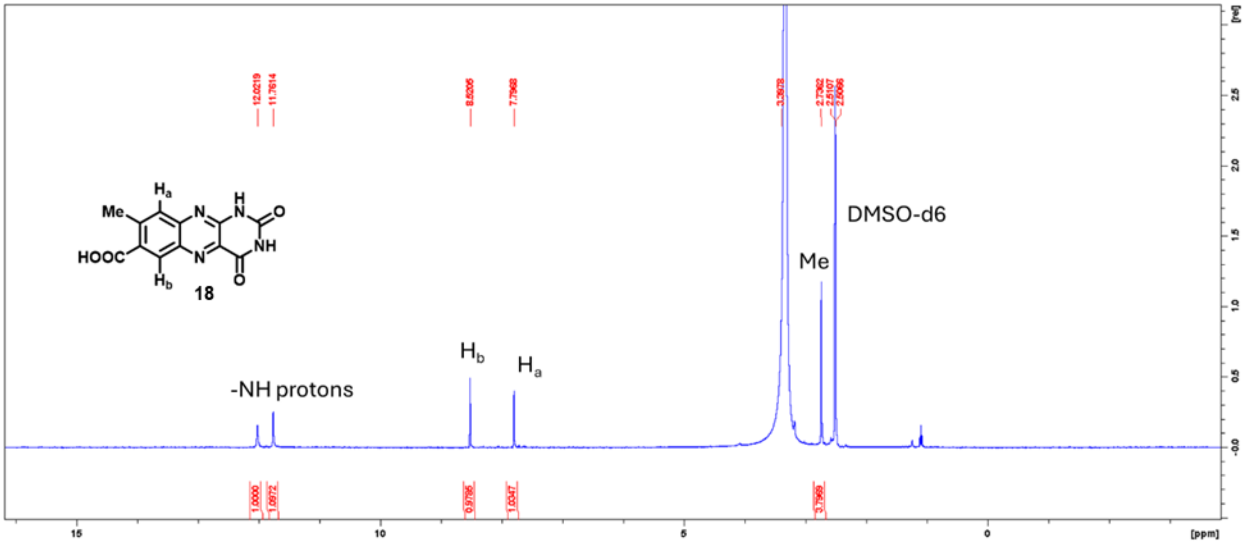

Figure S4. ^{1}H NMR of compound **18**.

Characterization of LumU

Overexpression and Purification of LumU

The gene in pTHT (a derivative of pET28b vector with TEV protease cleavage site after an N-terminal His-tag) was synthesized by GenScript. This was transformed into *E. coli* BL21(*DE3*) competent cells. Colonies obtained were grown in a 100 mL culture of LB with kanamycin (40 µg/mL) for 12 h at 37 °C (220 rpm). This culture was used to inoculate 1.5 L of LB media at 37 °C. At OD_{600} of 0.2, 100 mg iron (II) ammonium sulfate, 100 mg L-cysteine, and 250 mg aminolevulinic acid were added to 1.5 L of culture. When OD_{600} ~ 0.6 was reached, 0.5 mM of IPTG was added and the cells were grown for 18 h at 15 °C (120 rpm). Centrifugation was done to harvest the cells for 15 min at 5000 rpm and the harvested cells were stored in liquid nitrogen. Typical yields were 15 g of cells (wet weight) from 4.5 L of cell culture.

For purification, harvested cells were thawed and resuspended in 65 mL phosphate lysis buffer (100 mM KPi, 150 mM NaCl, pH 7.5). Lysozyme (30 mg) and benzonase nuclease (2 µL) were added and the suspension was stirred in an ice bath for 30 min. Cells were lysed by sonication and the mixture subjected to centrifugation at 15,000 rpm for 40 min to remove the cell debris. The supernatant was filtered using 0.22 µm filters and loaded onto a Ni-NTA His-trap column pre-equilibrated in lysis buffer. The column was then washed with 100 mL of wash buffer (100 mM KPi, 20 mM imidazole, 150 mM NaCl, pH 7.5) and 50 mL of the same buffer with increased imidazole concentration (70 mM). To elute protein, elution buffer (100 mM KPi, 250 mM imidazole, 150 mM NaCl, pH 7.5) was run through the column and fractions of the elute were collected. Fractions containing the protein were pooled and concentrated using 15 mL 10 kDa filters. Desalting was done inside the glovebox. The buffer of the concentrated protein was exchanged using Cytiva PD-10 desalting columns to 100 mM KPi, 30% glycerol, pH 7.5. The desalted enzyme was pipetted to make aliquots and flash-frozen with liquid nitrogen and stored at -80 °C. Protein concentration was determined using the absorbance at 280 nm (A_{280}) and the extinction coefficient calculated by the ProtParam tool of the ExPASy proteomics server (ε_{280} = 57300 M^{-1} cm^{-1}).

Assay conditions

0.7 mM of lumichrome was incubated with 200 µM of LumU and 4 mM nicotinamide adenine dinucleotide (NADH) in 100 mM potassium phosphate pH 7.5 buffer for 20 min at 37 °C. The mixture was passed through 10 kDa PES filters to quench the enzymatic reaction and then analyzed by HPLC and LC-MS.

HPLC conditions and method

A. Water

B. 100 mM Potassium phosphate buffer, pH 6.6

C. Methanol

(Flow rate: 1 mL/min)

0 min – 100% B, 8 min – 10% A 90% B, 10 min – 25% A 60% B 15% C, 15 min – 20% A 30% B 50% C, 20 min – 18% A 20% B 62% C, 22 min – 15% A 10% B 75% C, 25 min – 25% A 75% B, 26 min – 100% B, 34 min – 100% B.

Assay results

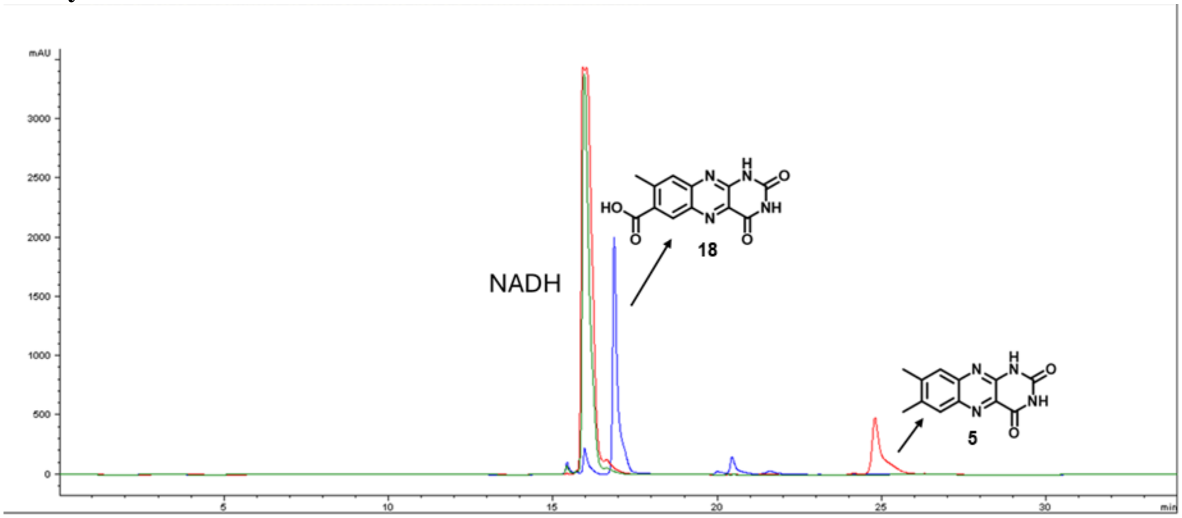

Figure S5. Full chromatogram (340 nm) showing formation of 7-carboxylumichrome (**18**) in the LumU reaction. Blue trace represents the LumU reaction, red trace shows the control without any LumU and green trace shows the no substrate control. NAD^+ is not visible at this wavelength.

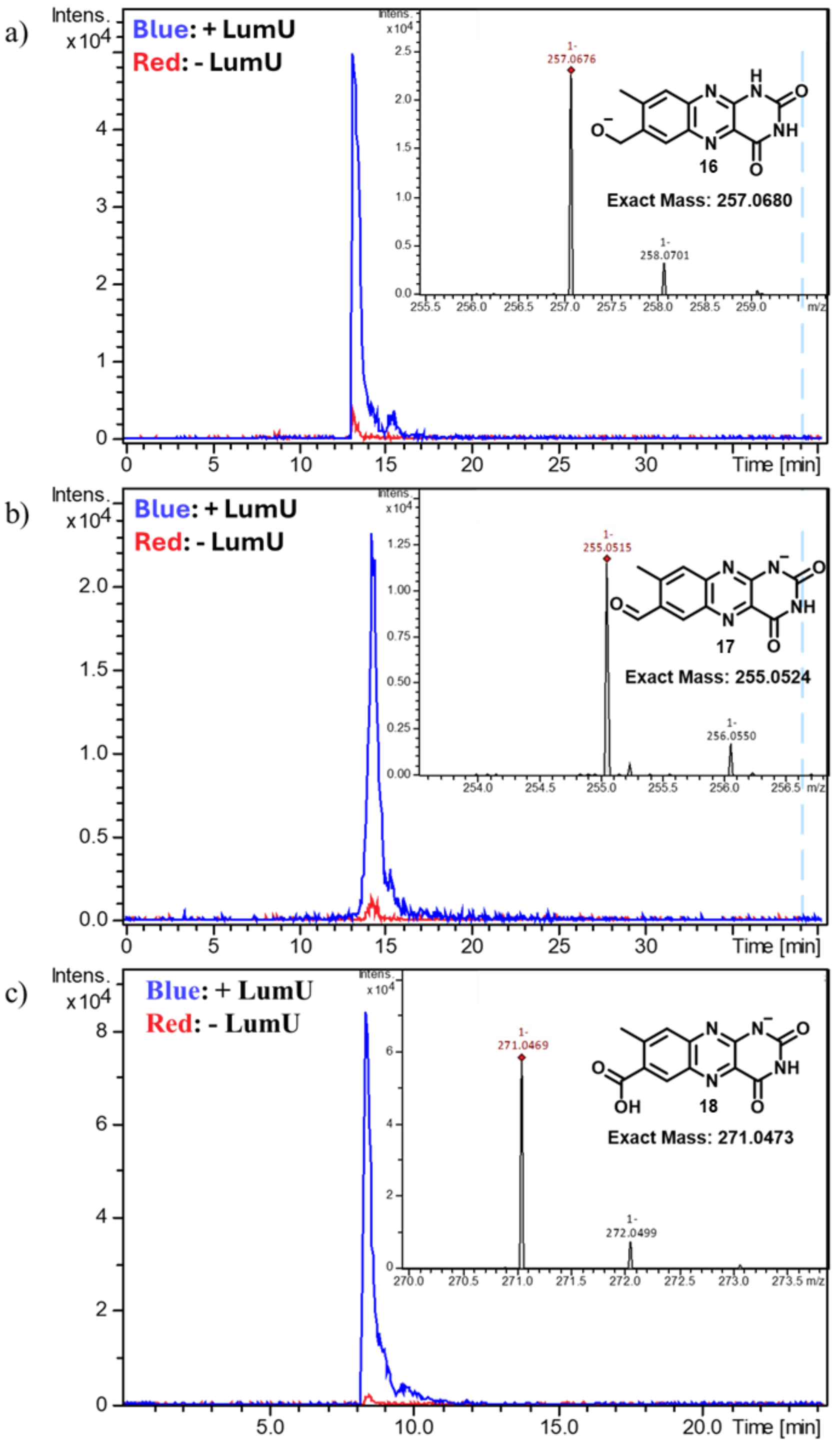

Figure S6. LC-MS ESI data showing mass of a) 7-hydroxymethyllumichrome (**16**), *m/z* 257.0680 Da, b) 7-formyllumichrome (**17**), *m/z* 255.0524 Da, c) 7-carboxylumichrome (**18**), *m/z* 271.0473 Da. Inset shows the *m/z* of each product.

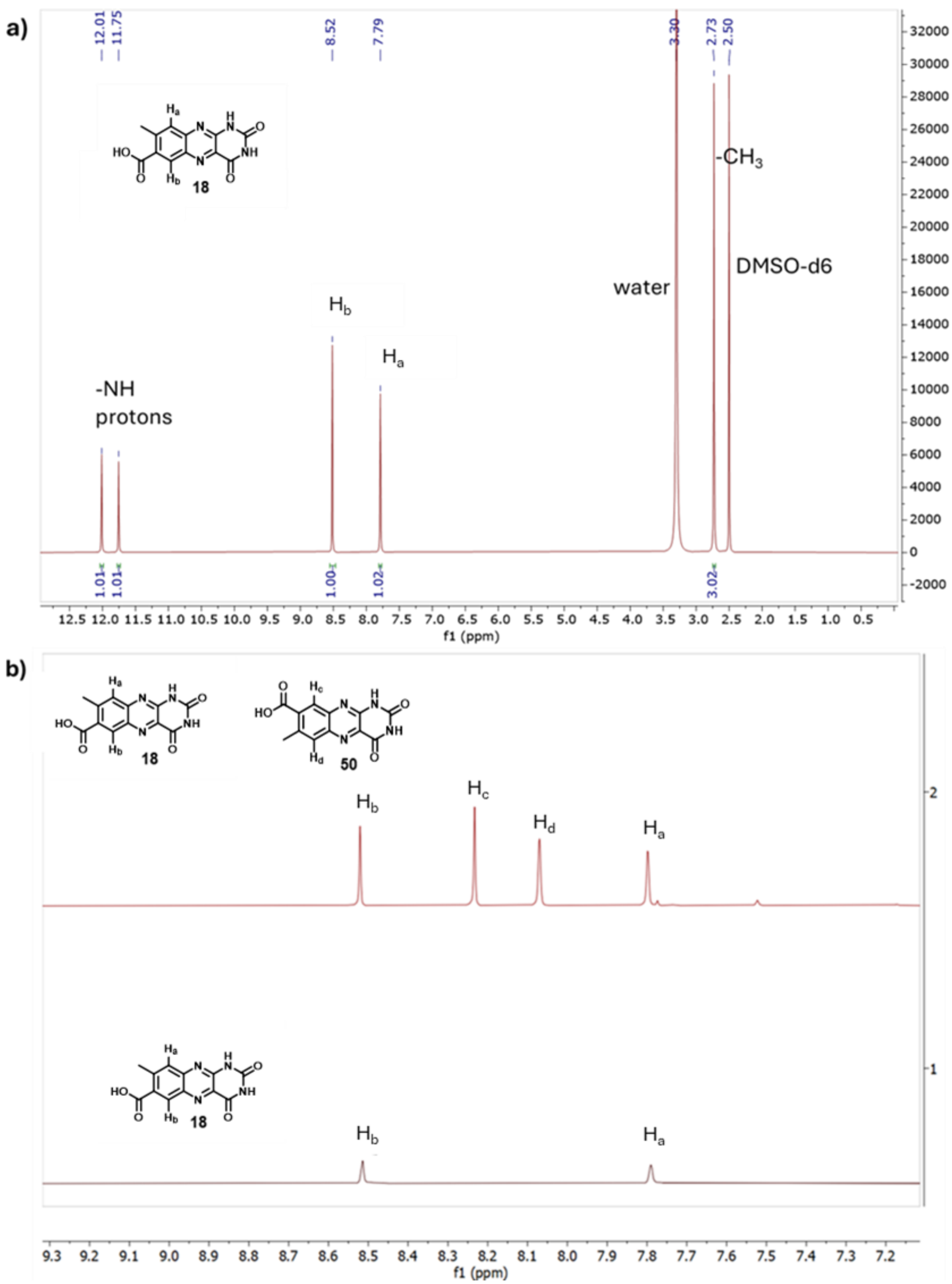

Figure S7. a) ^{1}H NMR of the product of the LumU-catalyzed reaction. Reference spectrum Figure S4. **b)** Aromatic protons compared to a synthesized mixture of 7-carboxylumichrome (**18**) and 8-carboxylumichrome (**50**).

Synthesis of 6-methyl-3-oxo-3,4-dihydroquinoxaline-2,7-dicarboxylic acid (21) and 3-amino-6-methylquinoxaline-2,7-dicarboxylic acid (20)

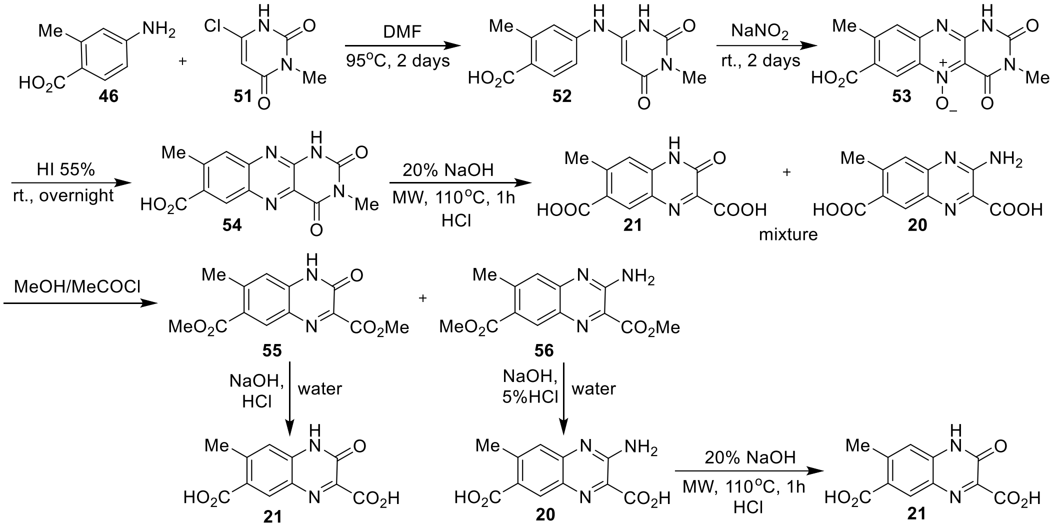

Figure S8. Synthetic scheme for 6-methyl-3-oxo-3,4-dihydroquinoxaline-2,7-dicarboxylic acid (**21**) and 3-amino-6-methylquinoxaline-2,7-dicarboxylic acid (**20**).

2-Methyl-4-(1-methyl-2,6-dioxo-1,2,3,6-tetrahydropyrimidin-4-yl) amino) benzoic acid (52)

Mixture of 6 g (0.04 mol) 4-amino-2-methylbenzoic acid (**46**) and 3.2 g (0.02 mol) 6-chloro-3-methylpyrimidine-2,4(1H,3H)-dione (**51**) in 20 mL of DMF was kept at 95 °C for 2 days. After cooling to room temperature, precipitate of 2-methyl-4-(1-methyl-2,6-dioxo-1,2,3,6-tetrahydropyrimidin-4-yl) amino) benzoic acid (**52**) was filtered out, washed with water, then methanol (5 mL of each) and dried at 65 °C overnight. Increasing reaction temperature to more than 100 °C leads to decarboxylation of starting material (**46**). The precipitated product from reaction mixture was pure and was used without further purification. ^{1}H NMR (400 MHz, DMSO-d_6) δ 12.57 (s, 1H), 10.60 (s, 1H), 8.60 (s, 1H), 7.87 (d, 1H), 7.10 (s, 1H), 7.07 (d, 1H), 5.09 (s, 1H), 3.31 (s, 3H), 2.53 (s, 3H).

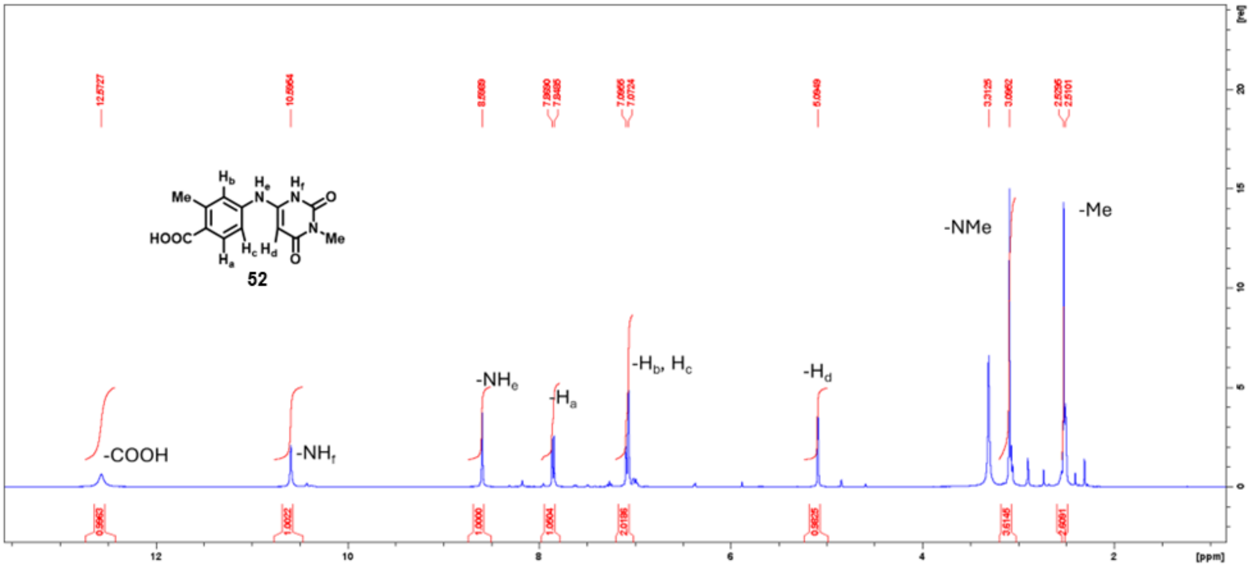

Figure S9. ^{1}H NMR of intermediate **52**.

7-Carboxy-3,8-dimethyl-2,4-dioxo-1,2,3,4-tetrahydrobenzo[g]pteridine 5-oxide (53)

785 mg (2.85 mmol) of 2-Methyl-4-(1-methyl-2,6-dioxo-1,2,3,6-tetrahydropyrimidin-4-yl) amino) benzoic acid (**52**) was crushed and placed in 5 mL of acetic acid. Suspension was sonicated for 5 min and stirred at room temperature for 2 days. Then, the suspension was diluted with 10 mL of fresh acetic acid followed by addition of 200 mg of $NaNO_2$ (2.7 mmol). After 5 h of stirring, a second portion of $NaNO_2$ (170 mg, 2.3 mmol) was added. Suspension was stirred overnight at room temperature and filtered. Solid product was washed with 3 mL of acetic acid, 3 mL of methanol and dried at 65 °C. 500 mg of 7-carboxy-3,8-dimethyl-2,4-dioxo-1,2,3,4-tetrahydrobenzo[g]pteridine 5-oxide (**53**) was obtained as a yellow solid. ^{1}H NMR (400 MHz, DMSO-d_6) δ 13.31 (s, 1H), 12.23 (s, 1H), 8.67 (s, 1H), 7.66 (s, 1H), 3.18 (s, 3H), 2.50 (s, 3H).

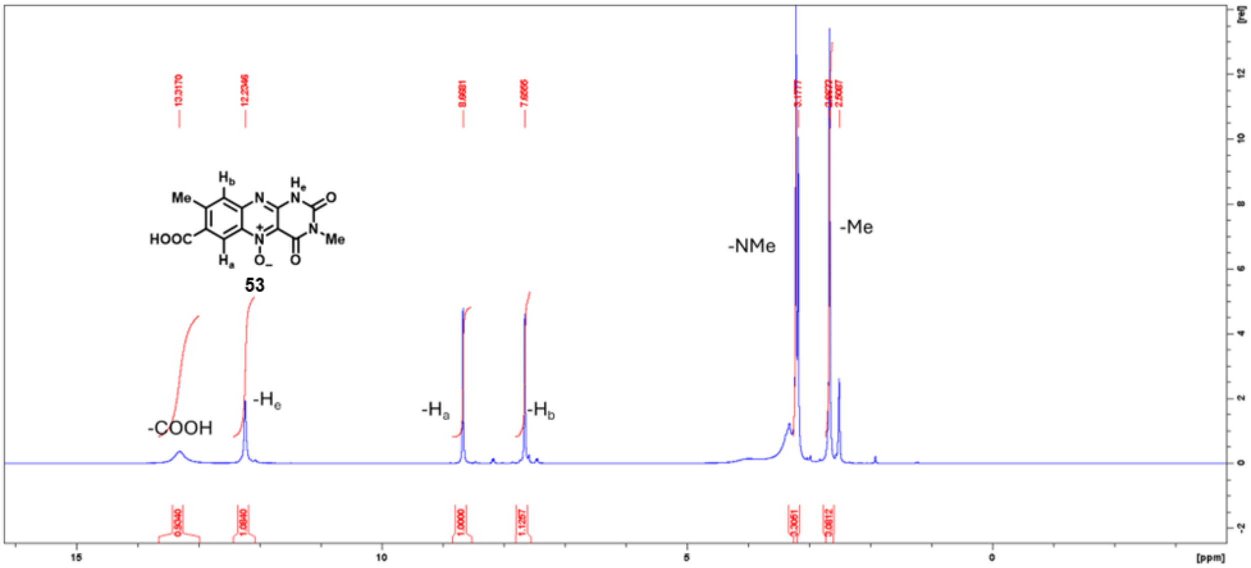

Figure S10. ^{1}H NMR of intermediate **53.**

3,8-Dimethyl-2,4-dioxo-1,2,3,4-tetrahydrobenzo[g]pteridine-7-carboxylic acid (54)

500 mg (1.7 mmol) of 7-carboxy-3,8-dimethyl-2,4-dioxo-1,2,3,4-tetrahydrobenzo[g] pteridine 5-oxide (**53**) and 5 mL of 55% HI were stirred at room temperature overnight, then filtered and the resulting dark solid washed with 2 mL of water, 5 mL of methanol and finally with 5 mL of ether. After drying at 65 °C, 400 mg of **54** was obtained. ^{1}H NMR (400 MHz, DMSO-d_6) δ 12.32 (s, 1H), 8.52 (s, 1H), 7.79 (s, 1H), 3.32 (s, 3H), 2.73 (s, 3H).

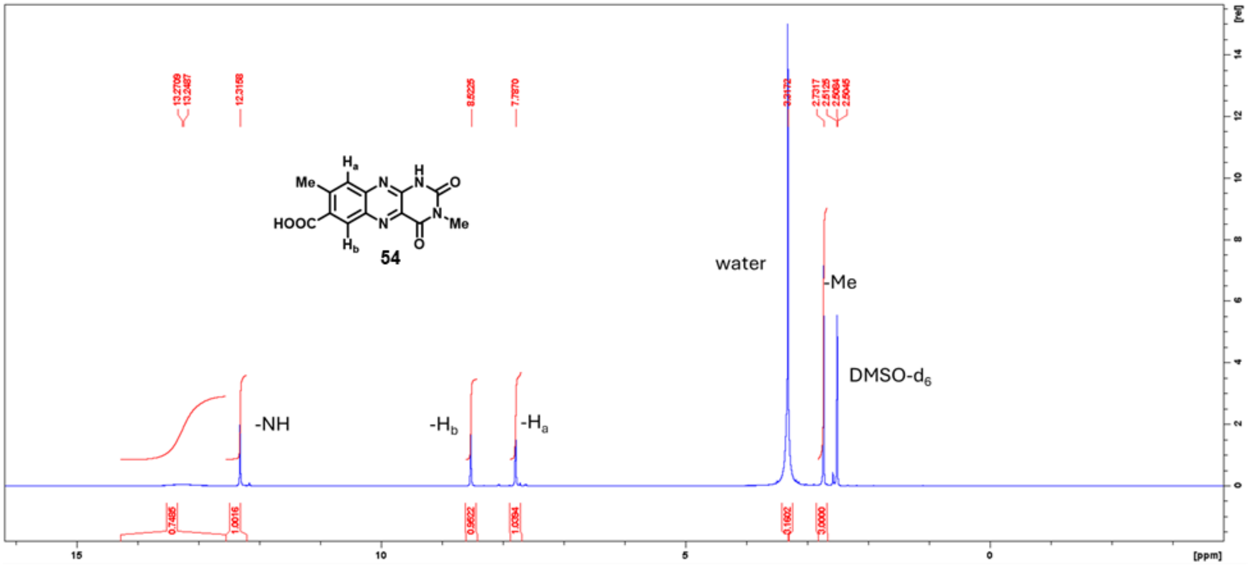

Figure S11. ^{1}H NMR of intermediate **54.**

Dimethyl 6-methyl-3-oxo-3,4-dihydroquinoxaline-2,7-dicarboxylate (55) and dimethyl 3-amino-6-methylquinoxaline-2,7-dicarboxylate (56)

130 mg of (**54**) in 2 mL 20% NaOH was heated in a microwave tube to 110 °C for 1 h, cooled to room temperature and acidified with 10% HCl upon precipitation of crude product. After filtration and drying at 65 °C, the crude material was dissolved in 3 mL of MeOH. 3 mL of acetyl chloride was added to this dropwise to avoid rapid heat evolution. After cooling, 10 mg of dimethyl 6-methyl-3-oxo-3,4-dihydroquinoxaline-2,7-dicarboxylate (**55**) formed a precipitate and was filtered out. ^{1}H NMR (400 MHz, DMSO-d_6) δ 12.97 (s, 1H), 8.20 (s, 1H), 7.17 (s, 1H), 3.91 (s, 3H), 3.85 (s, 3H), 2.60 (s, 3H).

The filtrate was evaporated, and the residue was passed through a silica gel column by washing with chloroform. Yellow color of the product was easily visible on the transparent silica gel when washing. After collection and evaporation of chloroform fractions, 15 mg of yellow solid dimethyl 3-amino-6-methylquinoxaline-2,7-dicarboxylate (**56**) was obtained. ^{1}H NMR (400 MHz, $CDCl_3$) δ 8.62 (s, 1H), 7.43 (s, 1H), 4.02 (s, 3H), 3.86 (s, 3H), 2.69 (s, 3H).

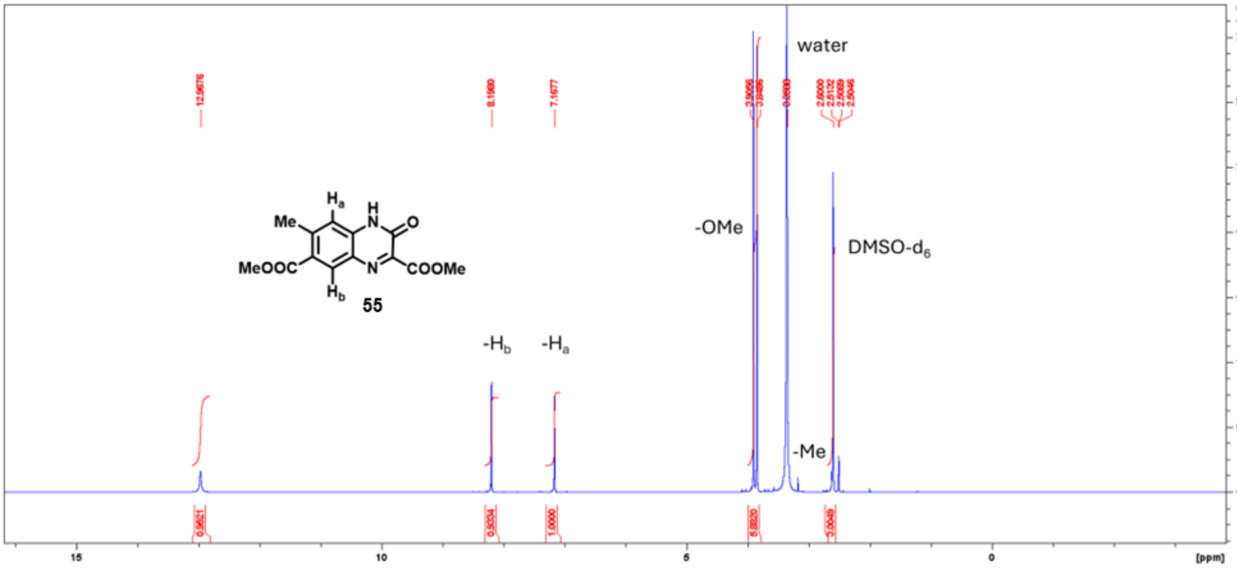

Figure S12. ^{1}H NMR of intermediate **55.**

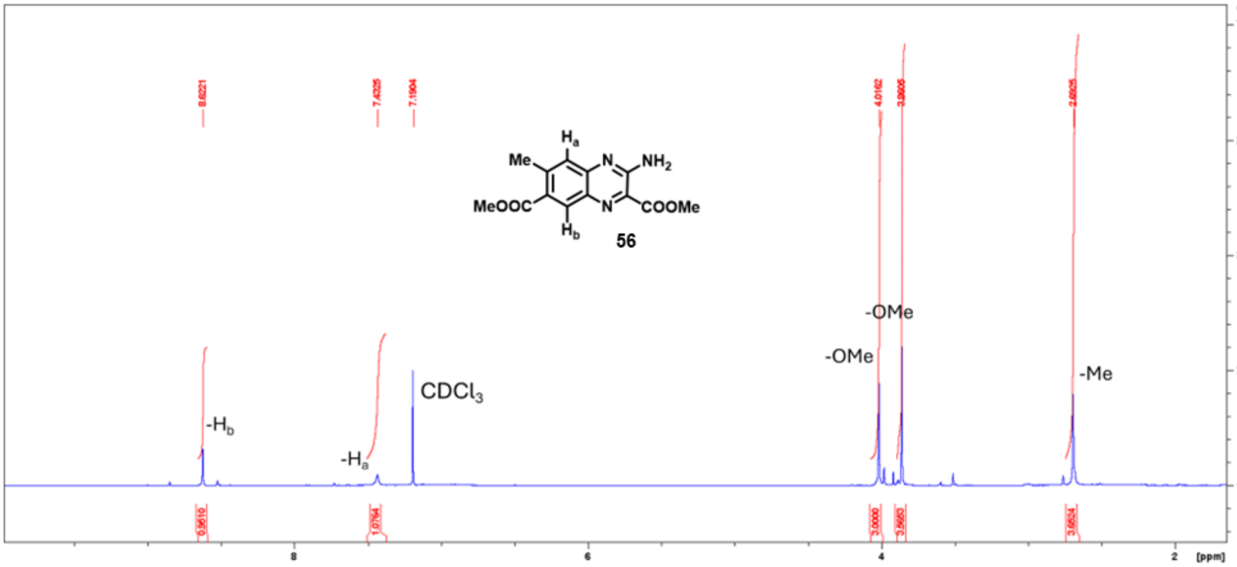

Figure S13. ^{1}H NMR of intermediate **56.**

6-methyl-3-oxo-3,4-dihydroquinoxaline-2,7-dicarboxylic acid (21)

10 mg of dimethyl 6-methyl-3-oxo-3,4-dihydroquinoxaline-2,7-dicarboxylate (**55**) was stirred with 15 mg of NaOH in 1 mL of water at room temperature overnight. The product was precipitated by the addition of 10% HCl, followed by filtration and drying. 10 mg of **21** was obtained. ^{1}H NMR (400 MHz, DMSO-d$_6$) δ 8.25 (s, 1H), 7.20 (s, 1H), 2.63 (s, 3H).

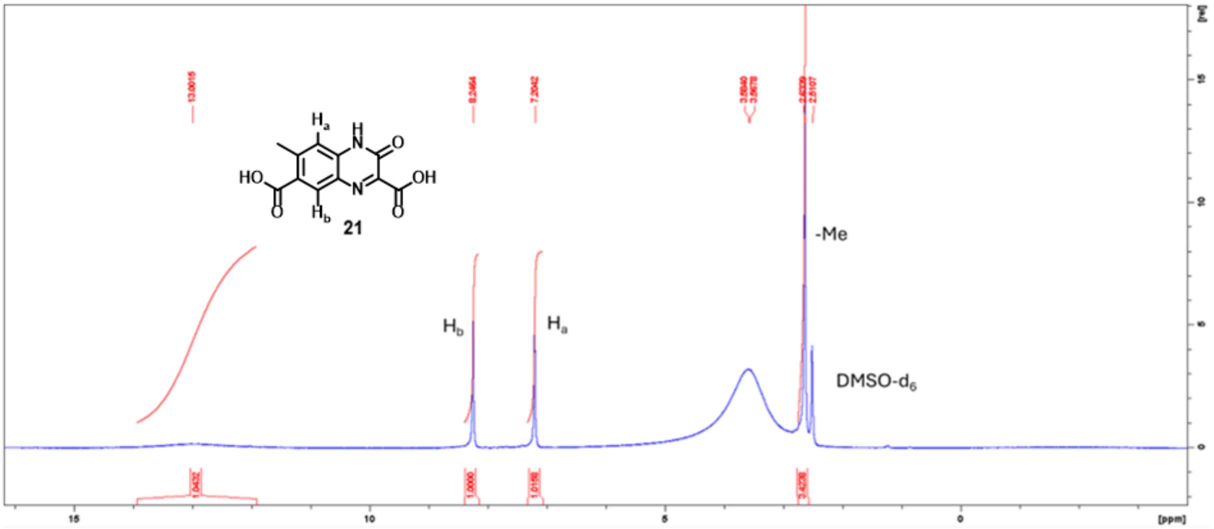

Figure S14. ^{1}H NMR of compound **21.**

3-Amino-6-methylquinoxaline-2,7-dicarboxylic acid (20)

15 mg of dimethyl 3-amino-6-methylquinoxaline-2,7-dicarboxylate **(56)** was stirred in 2 mL of water with 15 mg of NaOH overnight at room temperature. After acidification with 5% HCl, precipitate of the 3-amino-6-methylquinoxaline-2,7-dicarboxylic acid **(20)** was filtered out and

dried at room temperature. 11 mg of (**20)** was obtained. ^{1}H NMR (400 MHz, DMSO-d_6) δ 8.31 (s, 1H), 7.12 (s, 1H), 2.59 (s, 3H).

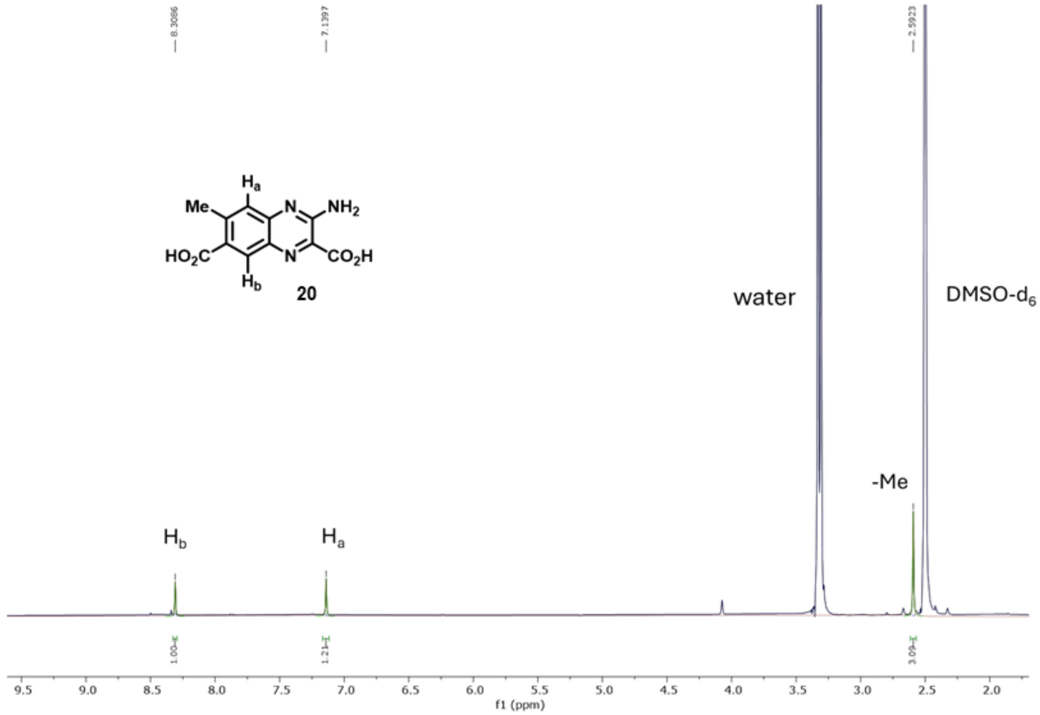

Figure S15. ^{1}H NMR of compound **20.**

3-amino-6-methylquinoxaline-2,7-dicarboxylic acid (**20**) with 20% NaOH under MW irradiation for 1 h at 110 °C provided 3-hydroxy-6-methylquinoxaline-2,7-dicarboxylic acid (**21**) quantitatively. NMR matches the product obtained from **55**.

Characterization of LumH, LumI, and LumM

Overexpression of Lum H, LumI, and LumM

LumH and LumI were overexpressed using the same protocol. The gene cloned in pTHT was synthesized by GenScript. This was transformed into *E.coli* BL21(*DE3*) competent cells. Colonies obtained were grown in a 100 mL culture of LB with kanamycin (40 µg/mL) for 12 h at 37 °C (220 rpm). This culture was used to inoculate 1.5 L of LB media at 37 °C. When OD_{600} of 0.6 was reached, 0.5 mM of IPTG was added and the cells were grown for 18 h at 15 °C (120 rpm). Cells were harvested for 15 min at 5000 rpm and then stored in liquid nitrogen. Typical yields were 15 g of cells (wet weight) from 4.5 L of cell culture.

For LumM overexpression, LumM in pTHT with an N-terminal His-tag was synthesized by GenScript. Overexpression was done utilizing Gro-EL Gro-ES chaperone proteins to improve LumM solubility. pGro-7 plasmid containing the chaperone proteins was transformed into *E.coli* BL21(*DE3*) competent cells. These were made chemically competent and LumM in pTHT was transformed into pGro-7 *E.coli* BL21(*DE3*) cells. Colonies obtained were grown in a 100 mL

culture of LB with kanamycin (40 μg/mL) and chloramphenicol (34 μg/mL) for 12 h at 37 °C (220 rpm). This culture was used to inoculate 1.5 L of LB media at 37 °C. At about OD_{600} of 0.2, 3 g of arabinose was added to 1.5 L of culture to induce overexpression of the chaperone proteins. When OD_{600} of 0.6 was reached, 0.5 mM of IPTG was added and the cells were grown for 18 h at 15 °C (120 rpm). Then the cells were harvested by centrifugation for 15 min at 5000 rpm and the harvested cells were stored in liquid nitrogen. Typical yields were 15 g of cells (wet weight) from 9 L of cell culture.

LumH, LumI, and LumM were purified using the same protocol. Harvested cells were thawed and resuspended in 65 mL phosphate lysis buffer (100 mM KPi, 150 mM NaCl, pH 7.5). Lysozyme (30 mg) and benzonase nuclease (2 μL) were added to this and the suspension was stirred in an ice bath for 30 min. Cells were lysed by sonication and the mixture subjected to centrifugation at 15,000 rpm for 40 min to remove the cell debris. The supernatant was filtered using 0.22 μm filters and loaded onto a Ni-NTA His-trap column pre-equilibrated in lysis buffer. The column was then washed with 100 mL of wash buffer (100 mM KPi, 20 mM imidazole, 150 mM NaCl, pH 7.5) and 50 mL of the same buffer with increased imidazole concentration (70 mM). To elute protein, elution buffer (100 mM KPi, 250 mM imidazole, 150 mM NaCl, pH 7.5) was run through the column and fractions of the elute were collected. The fractions were tested with Coomassie and the ones containing the protein were pooled and concentrated using 15 mL 10 kDa filters. The buffer of the concentrated protein was exchanged using Cytiva PD-10 desalting columns to 100 mM KPi, 30% glycerol, pH 7.5. The desalted enzyme was pipetted to make aliquots and flash-frozen with liquid nitrogen and stored at -80 °C. Protein concentration was determined using the absorbance at 280 nm (A_{280}) and the extinction coefficient calculated by the ProtParam tool of the ExPASy proteomics server (ε_{280} = 56380 M^{-1} cm^{-1} for LumH and ε_{280} = 13980 M^{-1} cm^{-1} for LumI and ε_{280} = 66920 M^{-1} cm^{-1} for LumM).

Assay conditions

500 μM of 7-carboxylumichrome (**18**) was incubated with 50 μM of LumH in 100 mM KPi pH 7.5 buffer for 6 h at 37 °C. The mixture was passed through 10 kDa PES filters to quench the enzymatic reaction. The reaction and the controls were analyzed by HPLC and LC-MS.

With the same substrate concentration, 50 μM of both LumH and LumI were added for the coupled LumH, LumI reaction. 50 μM of LumH, LumI, and LumM were added to 500 μM of **18** for LumH, LumI, LumM reaction.

HPLC conditions

A. Water

B. 100 mM Potassium phosphate buffer, pH 6.6

C. Methanol

HPLC method

(Flow rate: 1 mL/min)

0 min – 100% B, 8 min – 10%A 90% B, 10 min – 25% A 60% B 15% C, 15 min – 20% A 30% B 50% C, 20 min - 18% A 20% B 62%C, 22 min – 15%A 10% B 75%C, 25 min – 25%A 75% B, 26 min – 100% B, 34 min – 100% B.

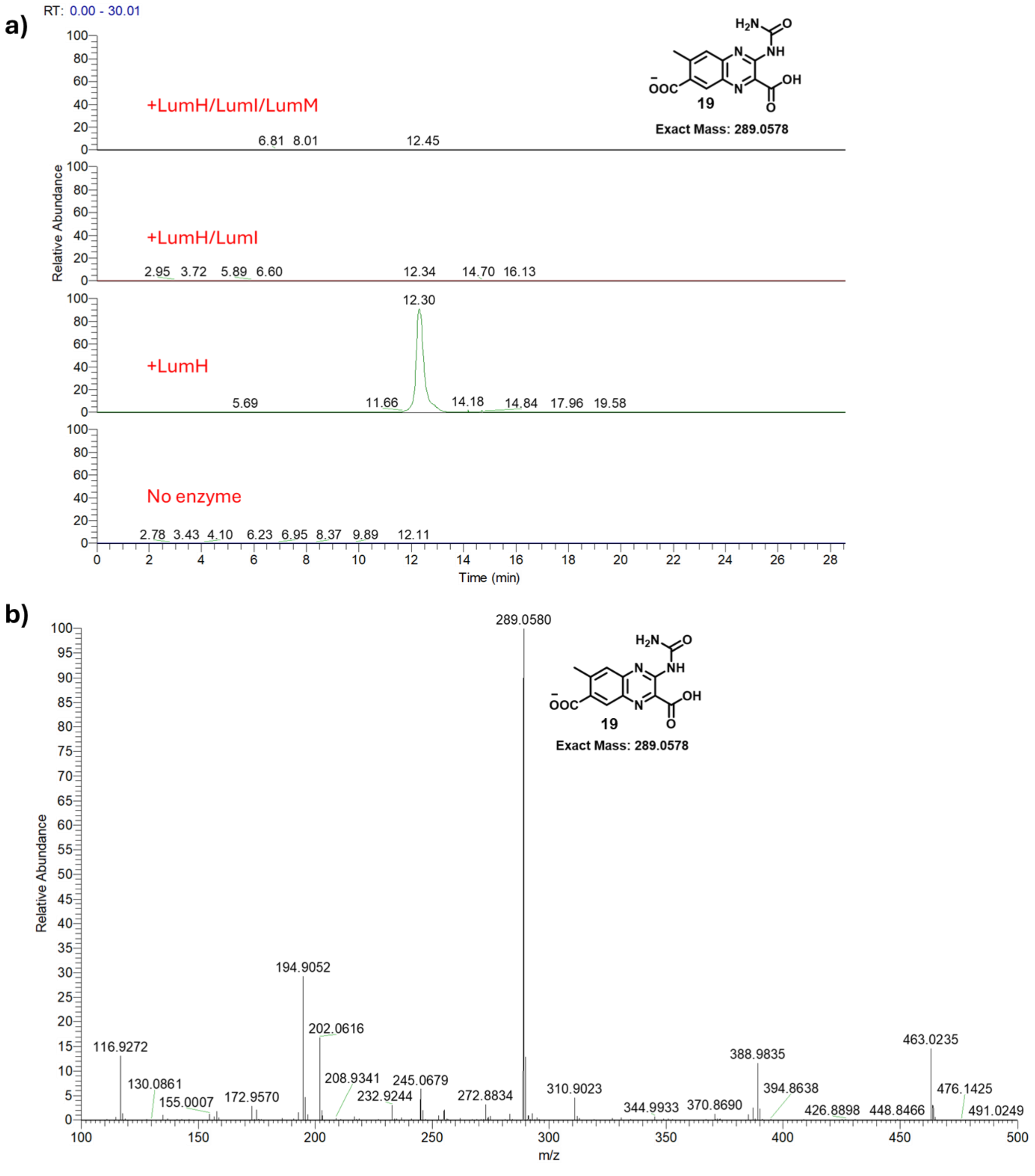

Figure S16. a) EIC of LumH product (*m/z* 289.0578 Da), **19**; b) MS of LumH reaction showing the *m/z* 289.0580 Da peak.

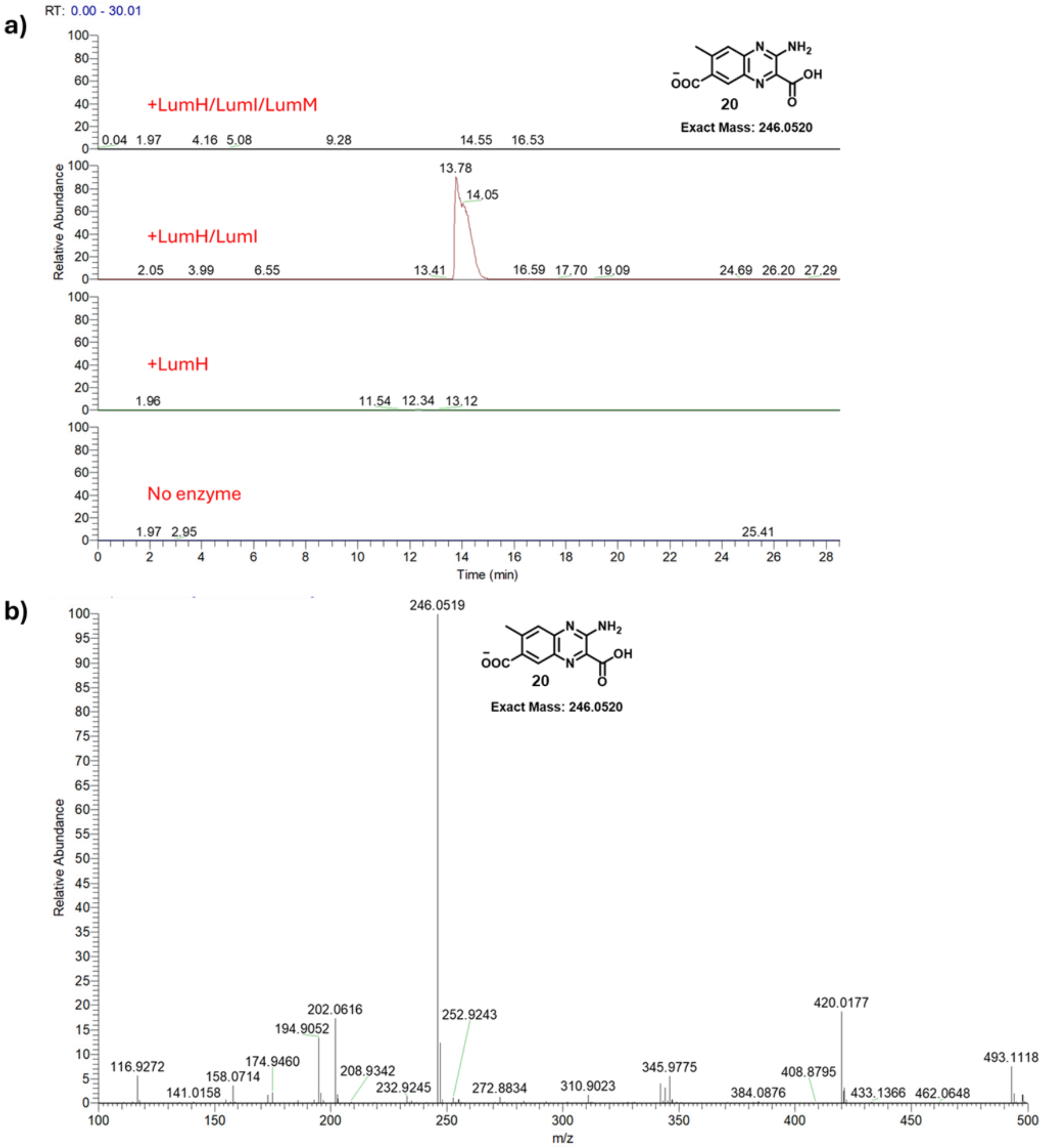

Figure S17. a) EIC of LumH/LumI product (*m/z* 246.0520 Da), **20**; b) MS of LumH/LumI reaction showing the *m/z* 246.0519 Da peak.

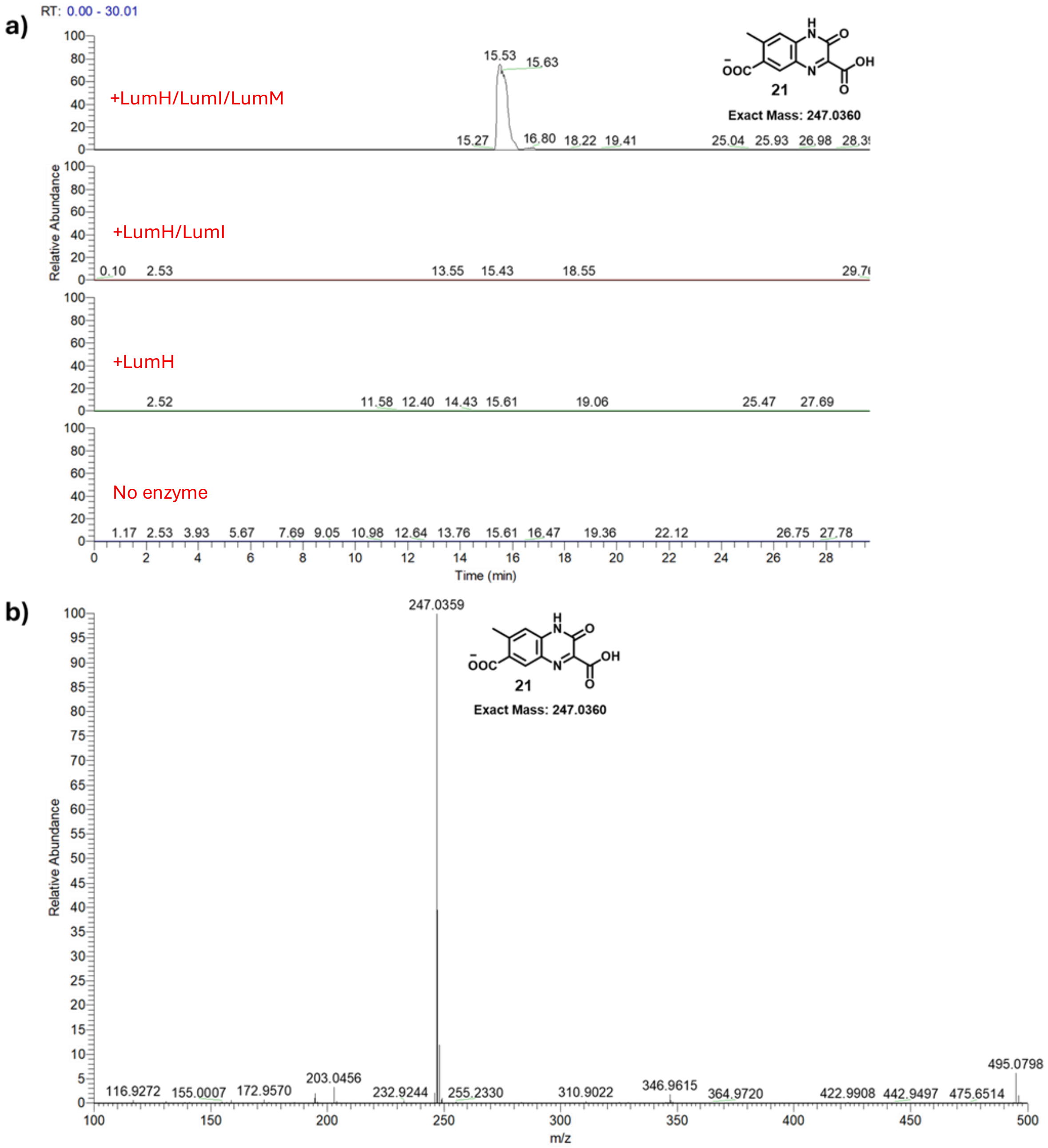

Figure S18. a) EIC of LumH/LumI/LumM product, **21** (*m/z* 247.0360 Da); b) MS of LumH/LumI/LumM reaction product showing the *m/z* 247.0359 Da peak of **21.**

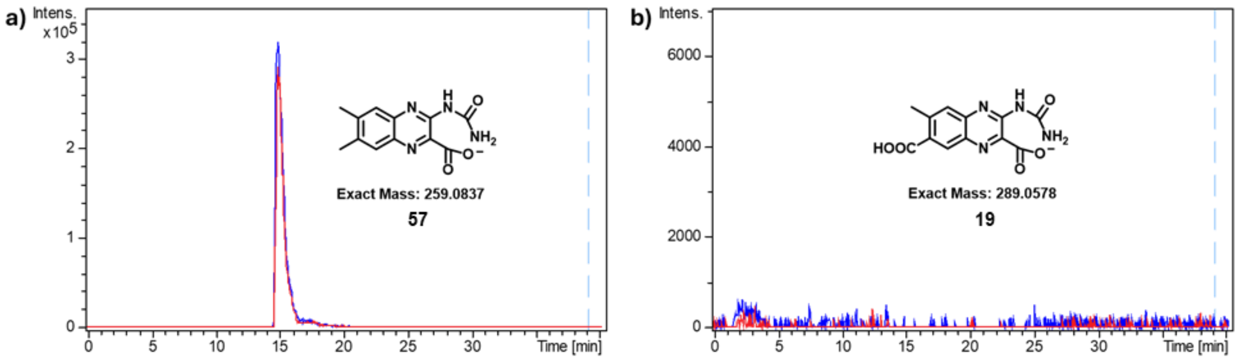

Figure S19. a) Compound **57** is not a substrate for LumU (EIC of m/z 259.0837 Da); b) Compound **19** is not formed when **57** is incubated with LumU (EIC of m/z 289.0578 Da). This suggests that the oxidation of the C7 methyl group precedes the hydrolysis of the pyrimidine ring. The blue trace represents the LumU reaction; the red trace shows the no-enzyme control.

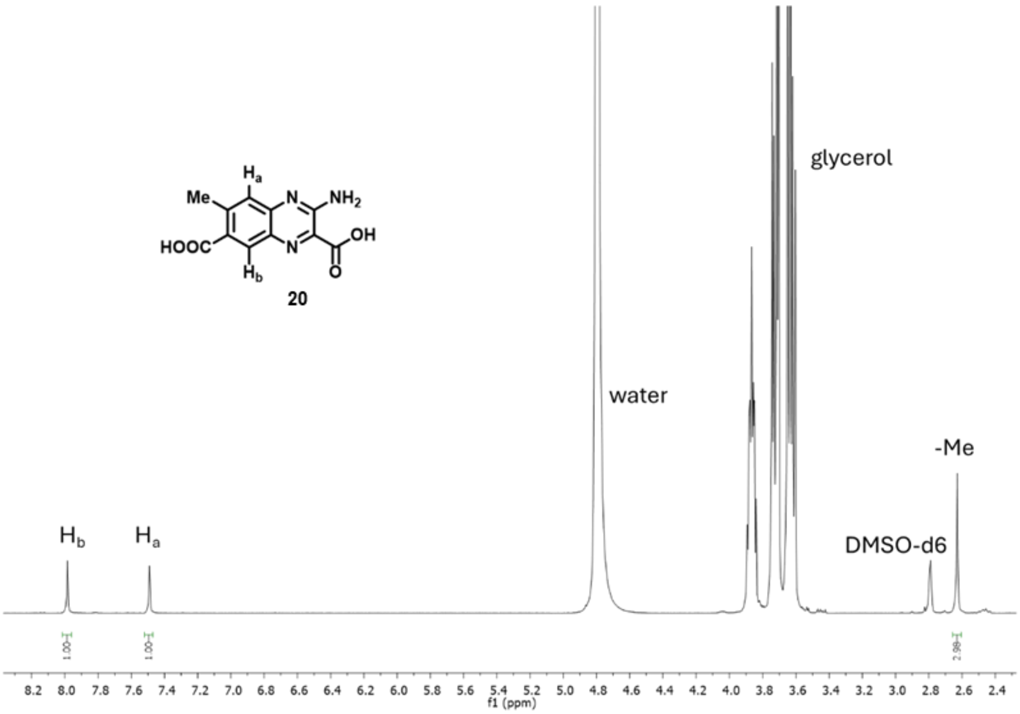

Figure S20. ^{1}H NMR of the product of the LumH and LumI-catalyzed reaction, **20** (Reference spectrum: Figure S15).

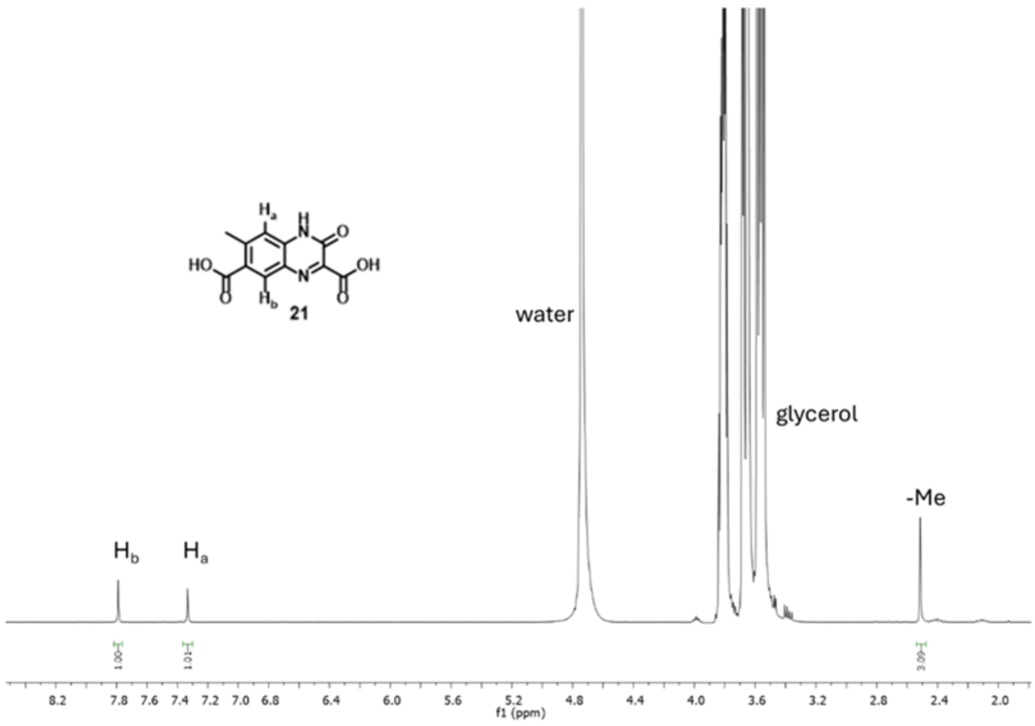

Figure S21. ^{1}H NMR of the product of the LumH, LumI, and LumM-catalyzed reaction, **21** (Reference spectrum: Figure S14).

Synthesis of 7-methyl-2-oxo-1,2-dihydroquinoxaline-6-carboxylic acid (22)

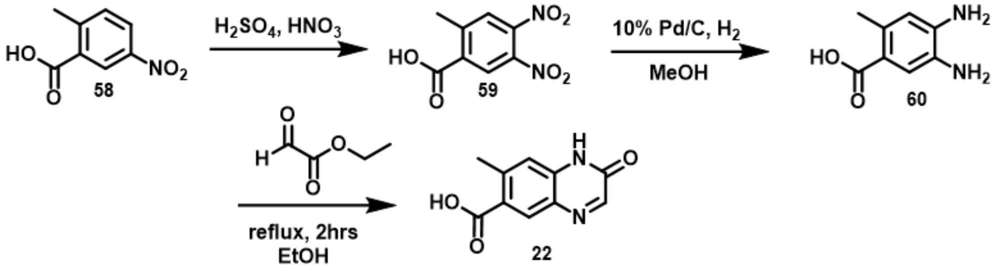

Figure S22. Synthetic scheme for LumK/LumL product **(22).**

2-Methyl-4,5-dinitrobenzoic Acid (59)

Compound **59** was prepared according to a reported synthesis.[1] To 50 mL of concentrated sulfuric acid at 0 ºC , 50 mL of concentrated nitric acid was added dropwise, and this was stirred for an additional 10 min. 2- methyl-4-nitrobenzoic acid (**58**) (9.0 g, 49.71 mmol) was added to this mixture in portions. After stirring for 16 h at room temperature, the reaction was poured into ice-cold water. The resulting solid was collected by filtration and dried to obtain compound **59**. ^{1}H NMR (400 MHz, DMSO-d_6) δ 8.47 (s, 1H), 8.17 (s, 1H), 2.66 (s, 3H).

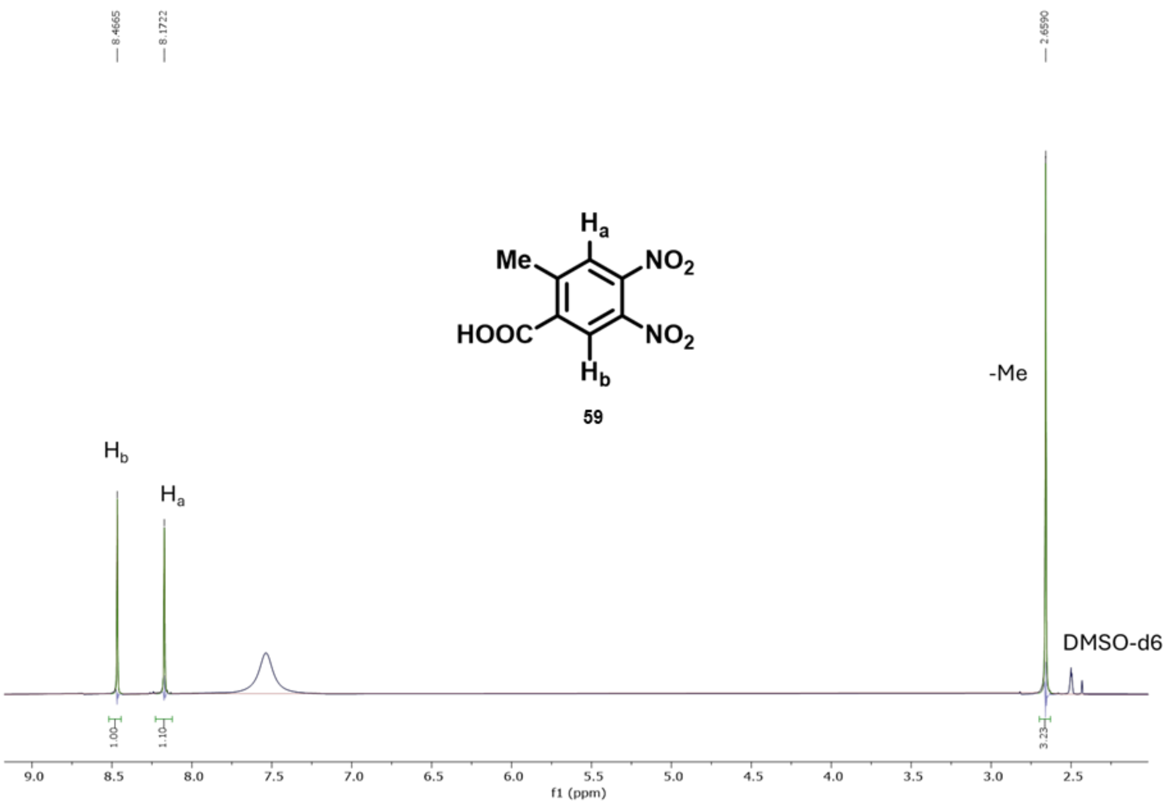

Figure S23. ^{1}H NMR of intermediate **59.**

2-Methyl-4,5-diaminobenzoic Acid (60)

2 g (10.25 mmol) of **59** was dissolved in methanol. 1 g of 10% Pd/C was added to this solution, and this was subjected to hydrogenation in a Parr apparatus overnight. At the end of the reaction, Pd/C was filtered out, and the supernatant was evaporated to obtain **60**. ^{1}H NMR (400 MHz, DMSO) δ 7.27 (s, 1H), 6.39 (s, 1H), 2.34 (s, 3H).

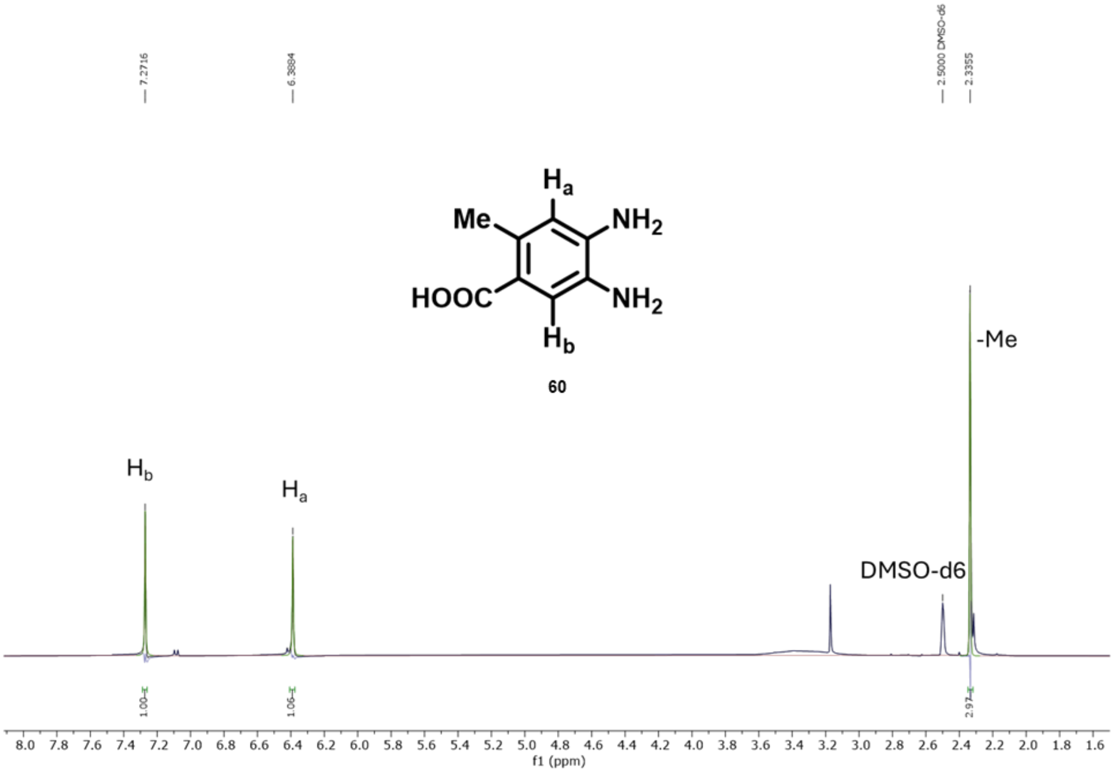

Figure S24. ^{1}H NMR of intermediate **60.**

7-methyl-2-oxo-1,2-dihydroquinoxaline-6-carboxylic acid (22)

A modified procedure from patent application WO2020065613A1 was followed.[2] 3,4-Diaminobenzoic acid (**59**) (2 g, 12 mmol, 1 eq) and ethyl glyoxylate (14 mL, 15 mmol, 1.2 eq) were refluxed in ethanol for 2 h. The precipitate was collected by vacuum filtration and dried. This mixture was HPLC purified using a C18 column. Collected fractions were lyophilized to obtain the desired product. Acetate peaks in the NMR are from residual ammonium acetate which was used as an HPLC buffer. ^{1}H NMR (400 MHz, H_2O+D_2O_salt) δ 8.27 – 8.17 (m, 1H), 7.85 (d, *J* = 1.7 Hz, 1H), 7.23 (d, *J* = 3.3 Hz, 1H), 2.55 (s, 3H), 1.97 (s, 1H).

Characterization of LumK and LumL

Overexpression and Purification of LumK and LumL

This enzyme consists of two subunits, LumK and LumL. LumL was obtained as a soluble protein, but LumK formed inclusion bodies during overexpression. After several attempts to get soluble LumK, co-expression with LumL led to soluble LumK and LumL as 1:1 mix. This mixture was used for the activity and other experiments.

LumK and LumL individually cloned in pTHT were synthesized by GenScript. LumK was subcloned into pEVOL vector. pEVOL-LumK and pTHT-LumL constructs were co-transformed in *E.coli* BL21(*DE3*). The cells were grown in LB medium at 37 °C in the presence of 50 μg mL^{-1} of kanamycin and 37 μg mL^{-1} of chloramphenicol. The media was supplemented with 5 mM of

magnesium chloride. At OD_{600} of 0.4, the cells were induced with 0.1 mM IPTG for the expression of LumL. Further, LumK expression was induced with 0.12% (wt./vol) arabinose 10 min after LumL induction, and the culture was grown at 25 °C for another 20 h at 180 rpm. The cells were then harvested by centrifugation (15 min, 4000 rpm) at 4 °C and stored at -80 °C.

The culture pellet was re-suspended in 50 mM phosphate buffer pH 8, containing 300 mM NaCl, 20 mM imidazole, and 1.2 mM TCEP. The cells were incubated on ice for 30 min in the presence of lysozyme and were lysed by sonication on ice. The lysate was centrifuged for 1 h at 20,000 g at 4 °C. The protein containing supernatant was then removed, filtered through a 0.45 µm filter, and loaded onto a HisTrap Ni-NTA column. The column was washed with 50 mM phosphate buffer pH 8, containing 300 mM NaCl, 20 mM imidazole, and 1.2 mM TCEP for 2 column volumes (CV), followed by a wash with the same buffer containing 40 mM imidazole for 3 CV. The elution of the proteins was carried out with buffer containing 250 mM imidazole. The elution fraction was concentrated using 10 kDa (MWCO) centrifugal filters, and the buffer exchange of the purified proteins was accomplished using a PD-10 column. The LumK and LumL protein mixture was eluted in 100 mM Tris-HCl, 20% glycerol, 1.2 mM TCEP, and pH 7.5.

Assay Conditions

0.5 mM of the substrate was incubated with 50 µM of the (1:1) LumKL mix and 200 µM thiamin pyrophosphate in 100 mM KPi pH 7.5 buffer for 1 h at 37 °C. The mixture was passed through 10 kDa PES filters to quench the enzymatic reaction. Decarboxylated product (**22**) mass was detected by LC-MS analysis, and the product structure was confirmed by HPLC coelution with a synthetic standard.

HPLC conditions

A. Water

B. 100 mM Potassium phosphate buffer, pH 6.6

C. Methanol

HPLC method:

(Flow rate: 1 mL/min)

0 min – 100% B, 8 min – 10%A 90%B, 10 min – 25% A 60% B 15% C, 15 min – 20% A 30% B 50% C, 20 min - 18% A 20% B 62%C, 22 min – 15%A 10% B 75%C, 25 min – 25%A 75% B, 26 min – 100% B, 34 min – 100% B.

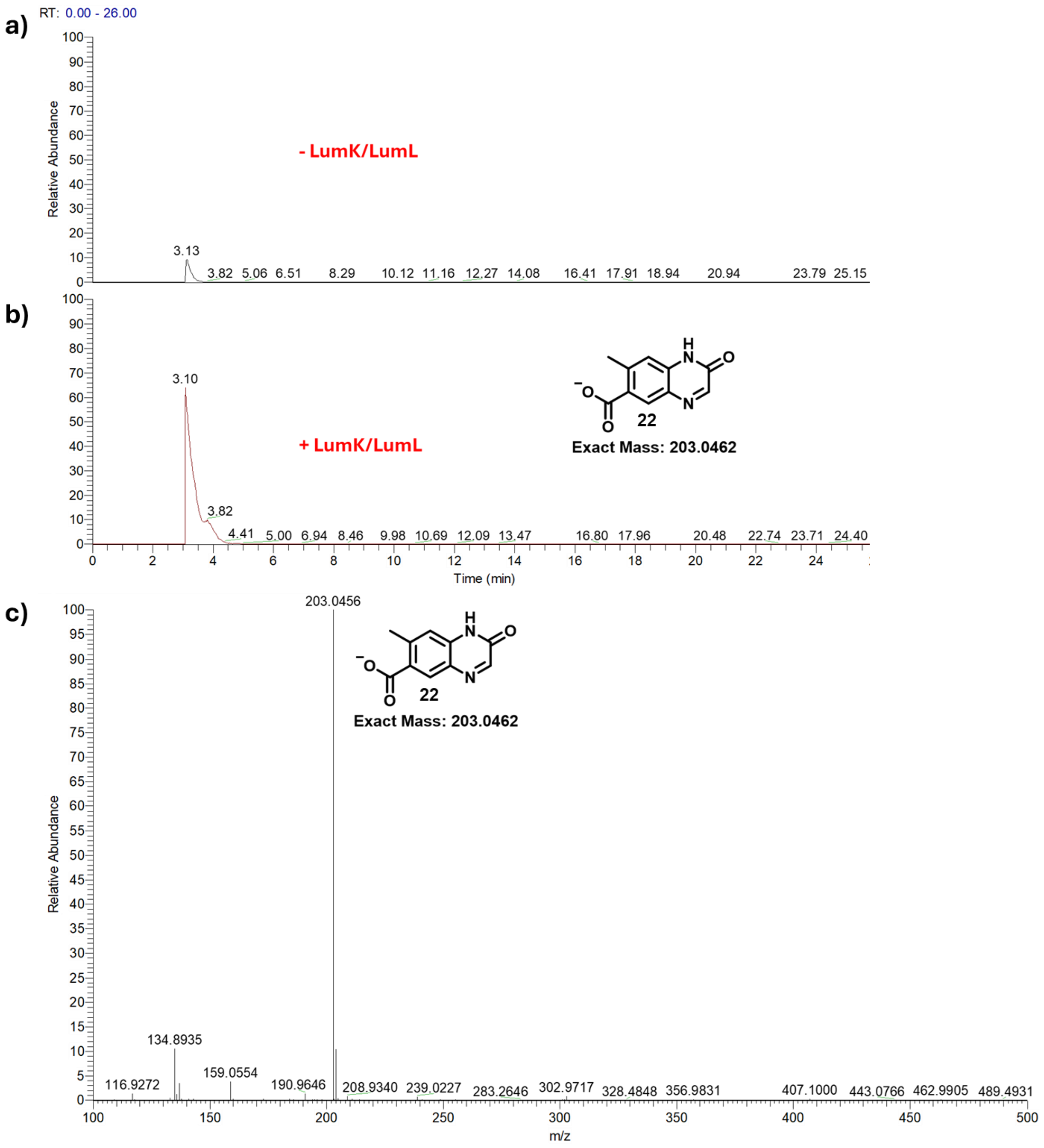

Figure S25. LC-MS of LumK/LumL reaction. EIC of LumK /LumL product, **22** (*m/z* 203.0462 Da) a) in presence of LumK/LumL; b) in absence of LumK/LumL; c) MS of LumK/LumL reaction showing the *m/z* 203.0456 Da peak.

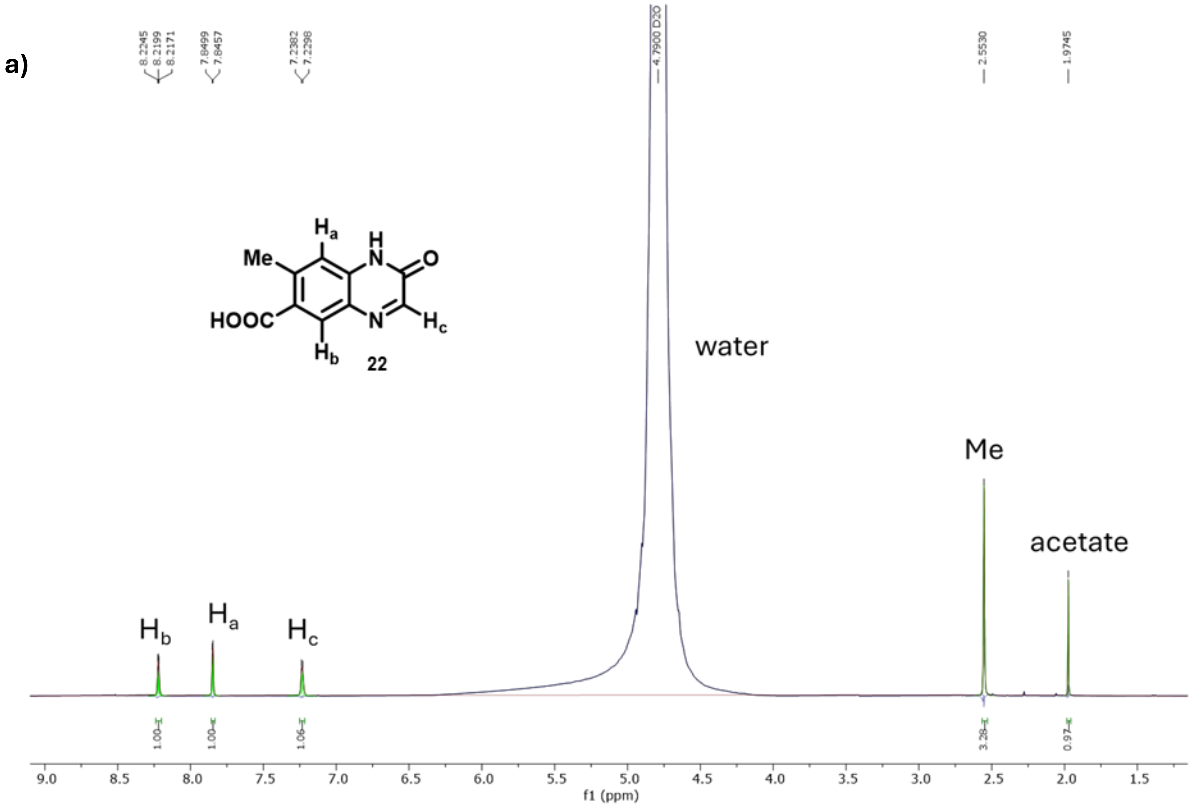

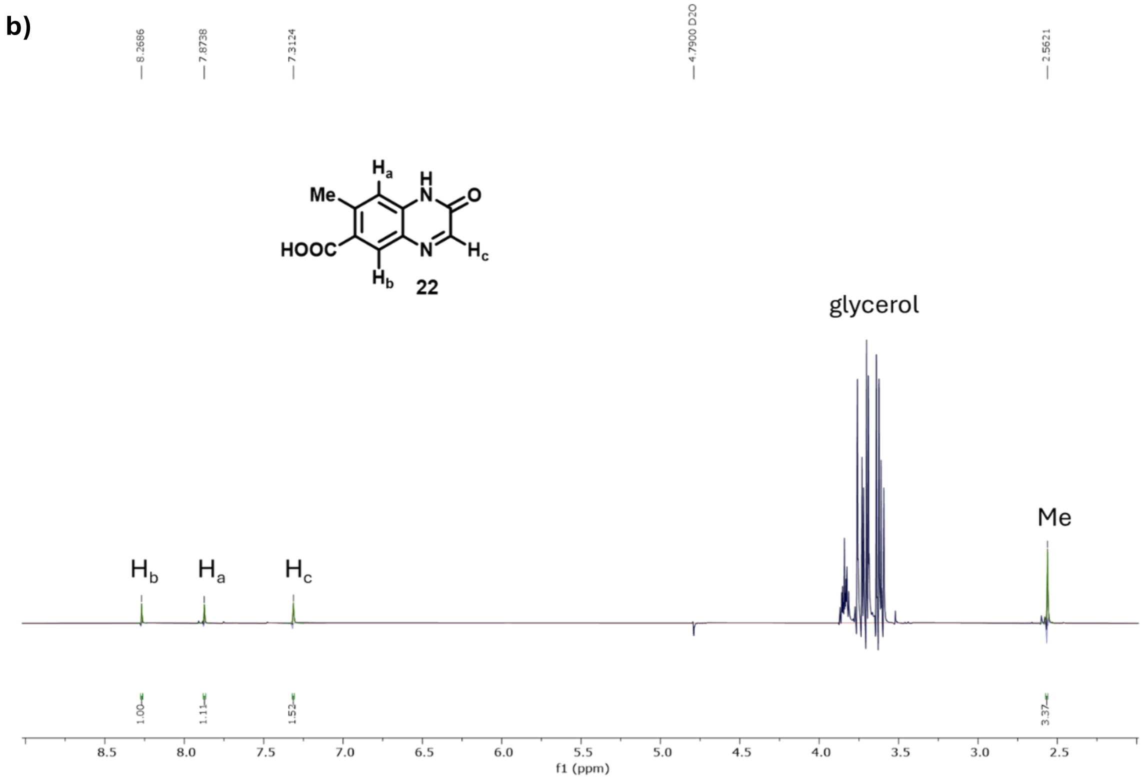

Figure S26. a) ^{1}H NMR of compound **22**; b) ^{1}H NMR of LumK/LumL collected product **22**.

Synthesis of 7-methyl-2,3-dioxo-1,2,3,4-tetrahydroquinoxaline-6-carboxylic acid 30a

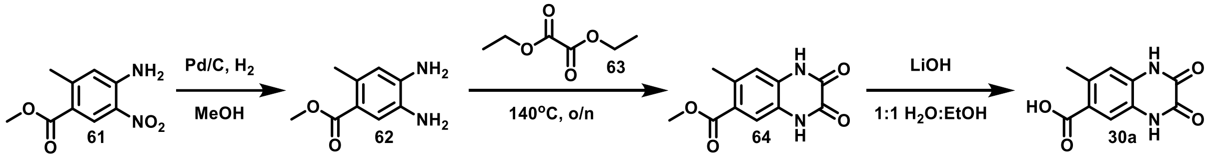

Figure S27. Synthetic scheme for LumJ product (**30a**).

Methyl 4,5-diamino-2-methylbenzoate (62)

500 mg of methyl 4-amino-2-methyl-5-nitrobenzoate (**61**), 250 mg of 10% Pd/C, and 10 mL anhydrous methanol were added to an argon-filled round bottom flask. The flask was flushed with a balloon of hydrogen gas and then the balloon was connected and left overnight. The next day, the reaction mixture was filtered using 0.45 μm filter and concentrated *in vacuo* to give a pure reduced product **62**. ^{1}H NMR (400 MHz, MeOD) δ 7.35 (s, 1H), 6.49 (s, 1H), 3.77 (s, 3H), 2.40 (s, 3H).

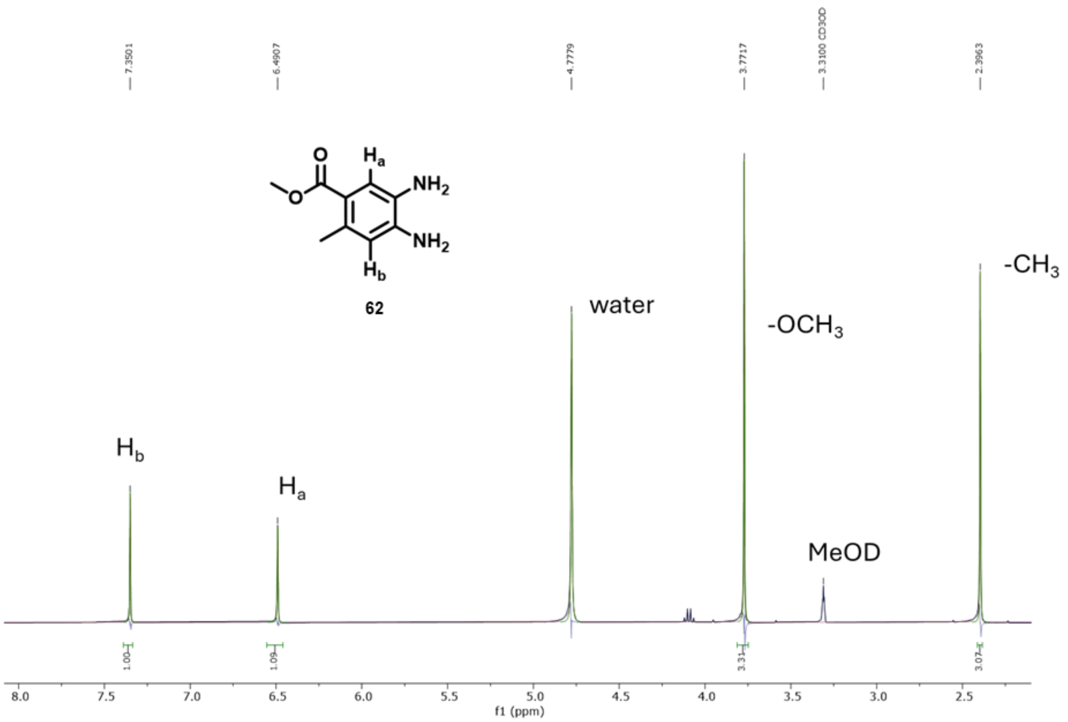

Figure S28. ^{1}H NMR of intermediate **62.**

Methyl 7-methyl-2,3-dioxo-1,2,3,4-tetrahydroquinoxaline-6 carboxylate (64)

Methyl 4,5-diamino-2-methylbenzoate (**62**) was stirred with diethyl oxalate (**63**) at 140 °C for 16 h. After cooling, the solid was collected by filtration, washed with diethyl ether and dried to obtain the product (**64**).^{1}H NMR (400 MHz, DMSO-d6) δ 7.68 (s, 1H), 6.99 (s, 1H), 3.81 (s, 3H), 2.48 (s, 3H).

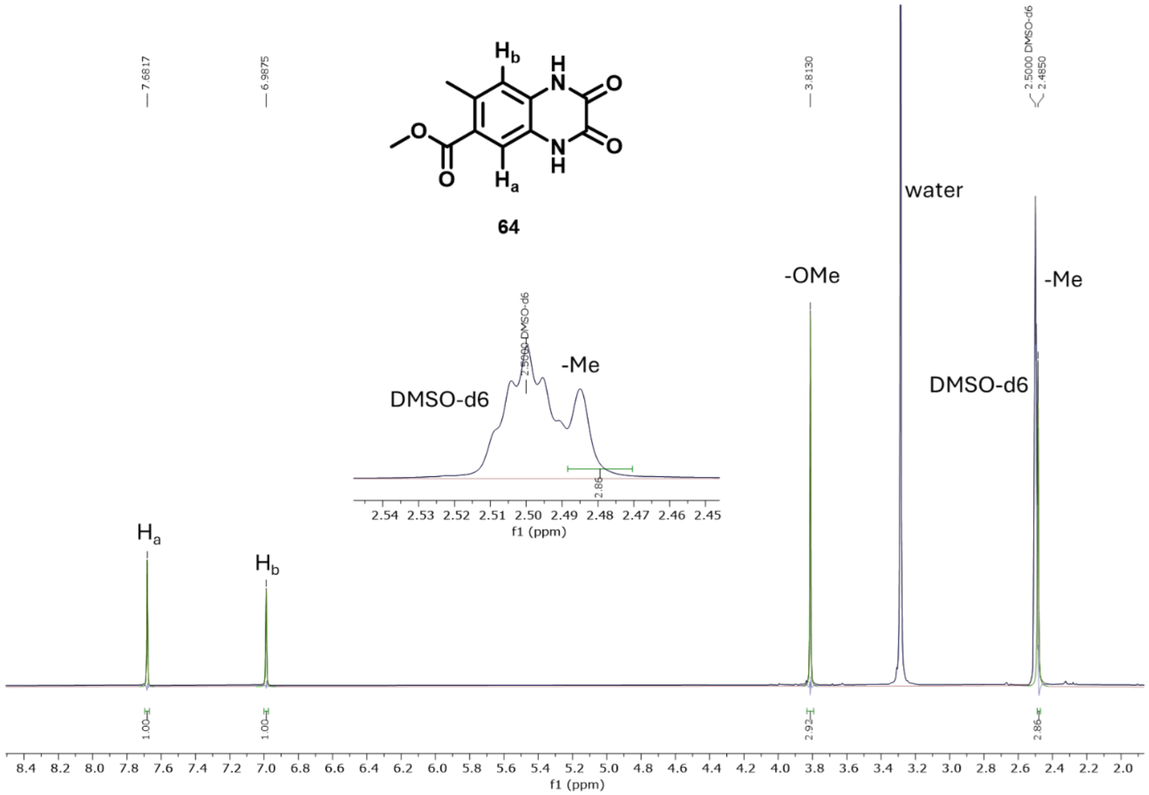

Figure S29. ^{1}H NMR of intermediate **64**. Inset shows region around DMSO-d_6.

7-methyl-2,3-dioxo-1,2,3,4-tetrahydroquinoxaline-6-carboxylic acid 30a

Compound **63** (50 mg, 0.21 mmol) was dissolved in 15 mL of 1:1 H_2O: EtOH, followed by addition of LiOH (42 mg, 1.05 mmol). This mixture was refluxed for 8 h. The solvent was concentrated in vacuo to obtain a light brown solid. ^{1}H NMR (400 MHz, D_2O_salt) δ 7.31 (s, 1H), 7.11 (s, 1H), 2.44 (s, 3H).

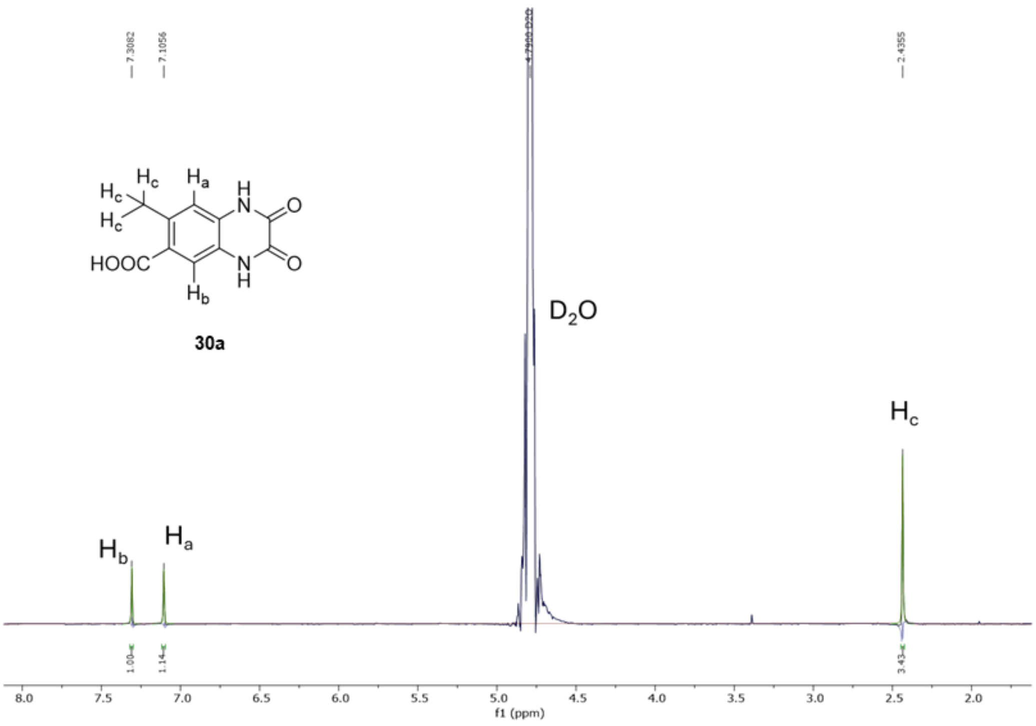

Figure S30. ^{1}H NMR of compound **30a.**

Characterization of LumJ

Overexpression and Purification of LumJ

The quinoxaline oxidase gene was cloned into a pMAL vector derivative. The plasmid was transformed into chemical-competent *E.coli* TP1000 cells (courtesy of Dr. Russ Hille, UC Riverside). The transformed colony was inoculated into a starter culture for 12 h. Then a larger (1.5 L) culture was inoculated with 15 mL of starter culture. Ampicillin, sodium molybdate salt (1 mM), L-cysteine (200 mg), ferrous ammonium sulfate (200 mg), and IPTG (20 µM) were added to the cultures which were shaken at 100 rpm, 22 °C for 24 h. The cells from the 9 L LB culture were collected with centrifugation and stored in liquid nitrogen until further use.

The protein purification was done inside an anaerobic chamber because the molybdopterin cofactor is oxygen-sensitive. Harvested cells were thawed and resuspended in 65 mL phosphate lysis buffer (100 mM KPi, 150 mM NaCl, pH 7.5). Lysozyme (30 mg) and benzonase nuclease (2 µL) were added to this, and the suspension was stirred in an ice bath for 30 min. Cells were lysed by sonication, and the mixture was subjected to centrifugation at 15,000 rpm for 40 min to remove the cell debris. The supernatant was loaded without filtering onto a Ni-NTA His-trap column pre-equilibrated in lysis buffer. The column was then washed with 100 mL of wash buffer (100 mM KPi, 20 mM imidazole, 150 mM NaCl, pH 7.5) and 50 mL of the same buffer with increased imidazole concentration (70 mM). To elute protein, elution buffer (100 mM KPi, 250 mM imidazole, 150 mM NaCl, pH 7.5) was run through the column, and fractions of the elute were

collected. These were checked for the protein by Western Blotting with anti-His-tag antibody (Genscript), and the protein-containing fractions were combined and made into aliquots that were stored in liquid nitrogen.

Assay conditions

A mixture of 0.5 mM substrate **22**, 2 mg/mL protein and 0.5mM phenazine methosulfate (PMS) in Tris buffer (pH 7.5) was incubated for 6 h at room temperature in an anaerobic chamber. To quench the reaction, it was passed through 10 kDa PES filters. The reaction was analyzed by HPLC, LC-MS, and NMR.

HPLC conditions

A. Water

B. 100 mM Potassium phosphate buffer, pH 6.6

C. Methanol

HPLC method

(Flow rate: 1 mL/min)

0 min – 100% B, 5 min – 10%A 90%B, 12 min – 48% A 40% B 12% C, 14 min – 50% A 30% B 20% C, 18 min - 30% A 10% B 60%C, 20 min – 100% B, 25 min – 100% B.

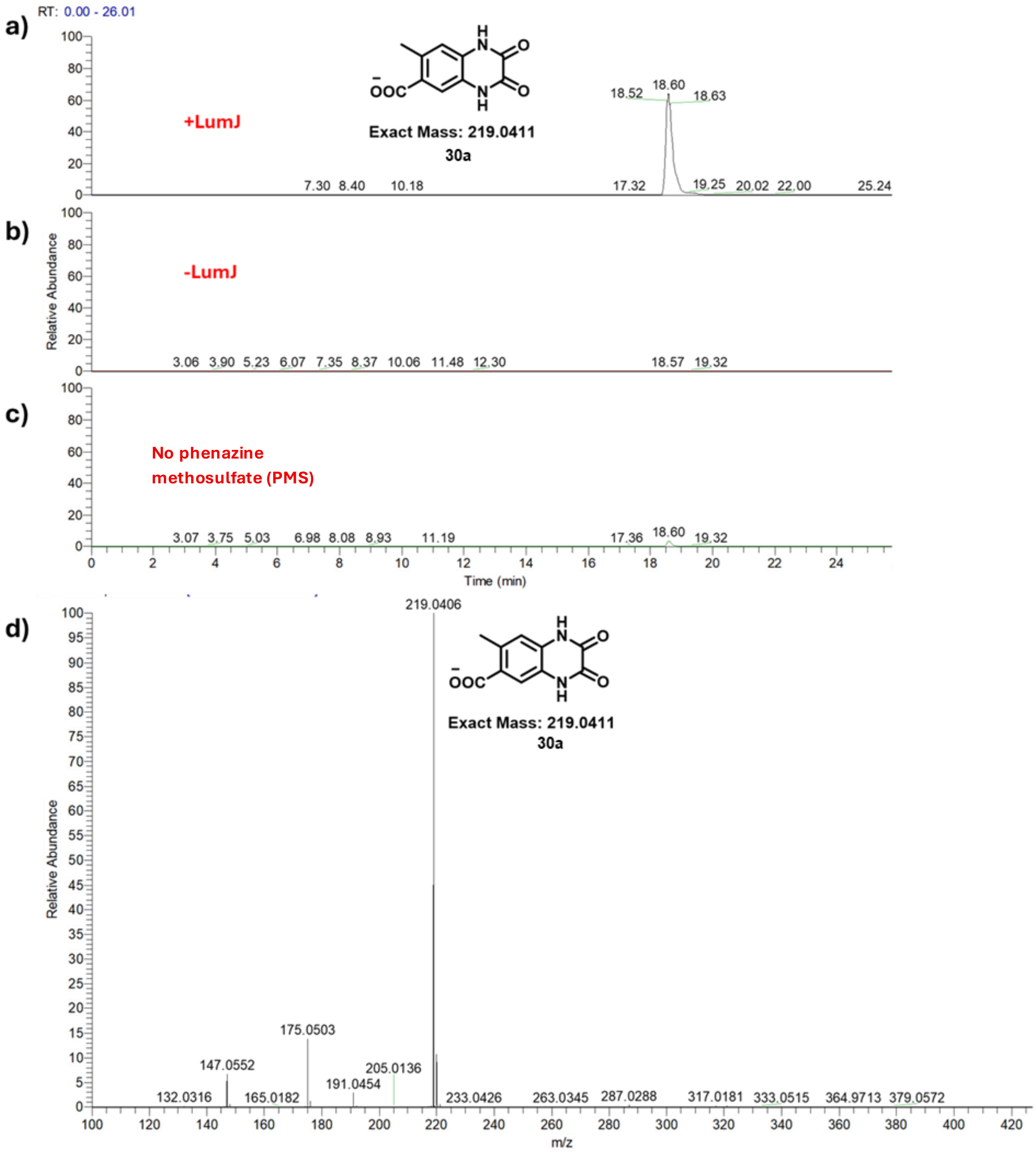

Figure S31. LC-MS data for LumJ product (**30a**) formation. a) EIC of LumJ product (*m/z* 219.0411 Da) in presence of LumJ and oxidizing agent; b) EIC of LumJ product (*m/z* 219.0411 Da) in presence of oxidizing agent but not LumJ; c) EIC of LumJ product (*m/z* 219.0411 Da) in presence of LumJ but absence of phenazine methosulfate (oxidizing agent); d) MS of LumJ reaction showing the *m/z* 219.0406 Da peak.

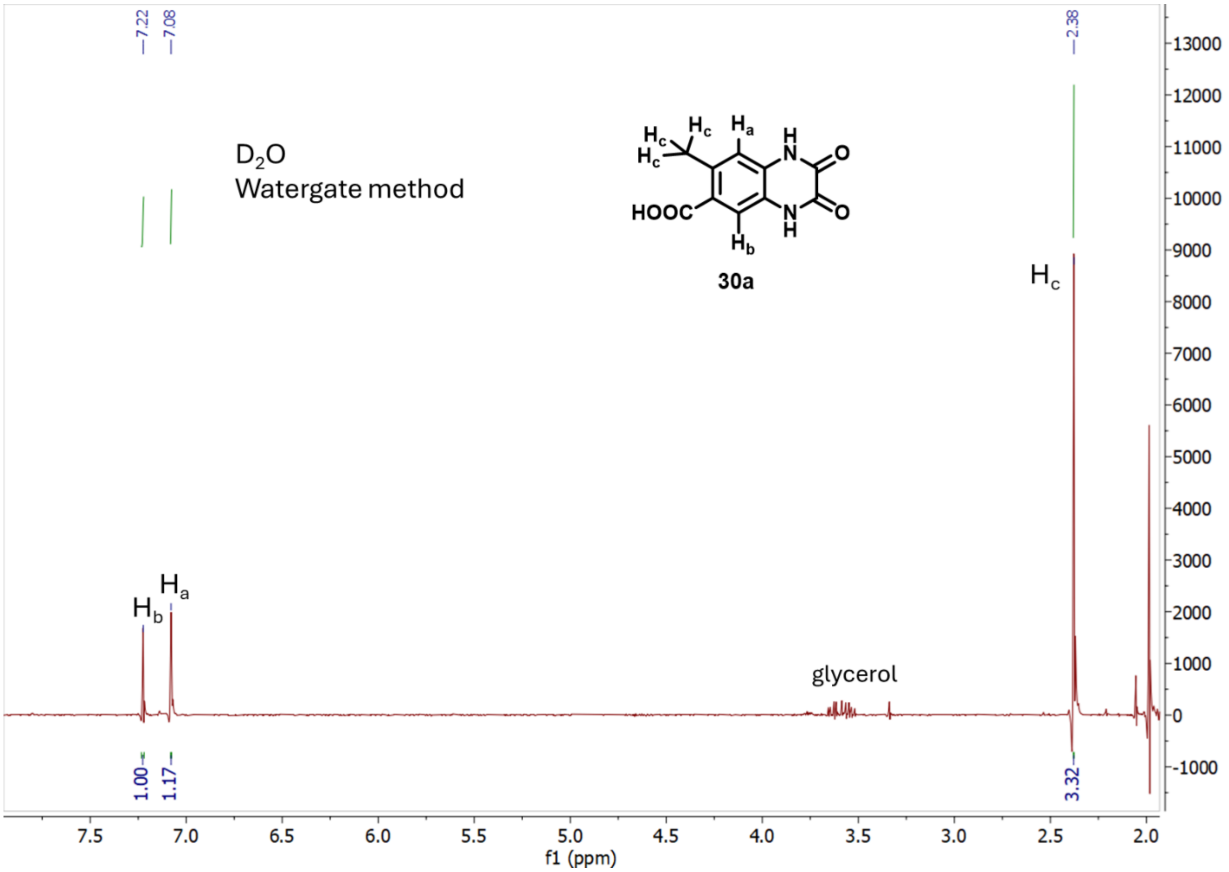

Figure S32. ^{1}H NMR of the product of the LumJ-catalyzed reaction, **30a** (Reference spectrum: Figure S30).

Synthesis of 4-(carboxyformamido)-2,3-dihydroxybenzoic acid (33b)

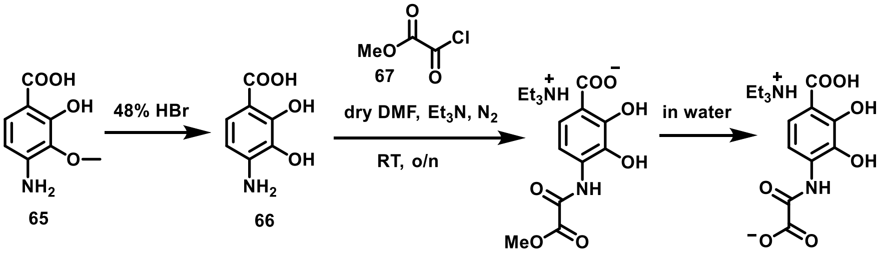

Figure S33. Synthetic scheme for LumA, LumB, LumC, and LumD product **33b**.

4-amino-2,3-dihydroxybenzoic acid (66)

100 mg of 4-amino-2-hydroxy-3-methoxybenzoic acid (**65**) was dissolved in 10 mL of 48% aq. HBr and heated at 110 °C overnight. The reaction mixture was concentrated *in vacuo* and resulted in a dark brown solid. $[M-H]^-$: 168.0292. ^{1}H NMR (400 MHz, DMSO-d$_6$) δ 7.45 (1H, d, J=8.60 Hz) 6.79 (1H, d, J=8.60 Hz).

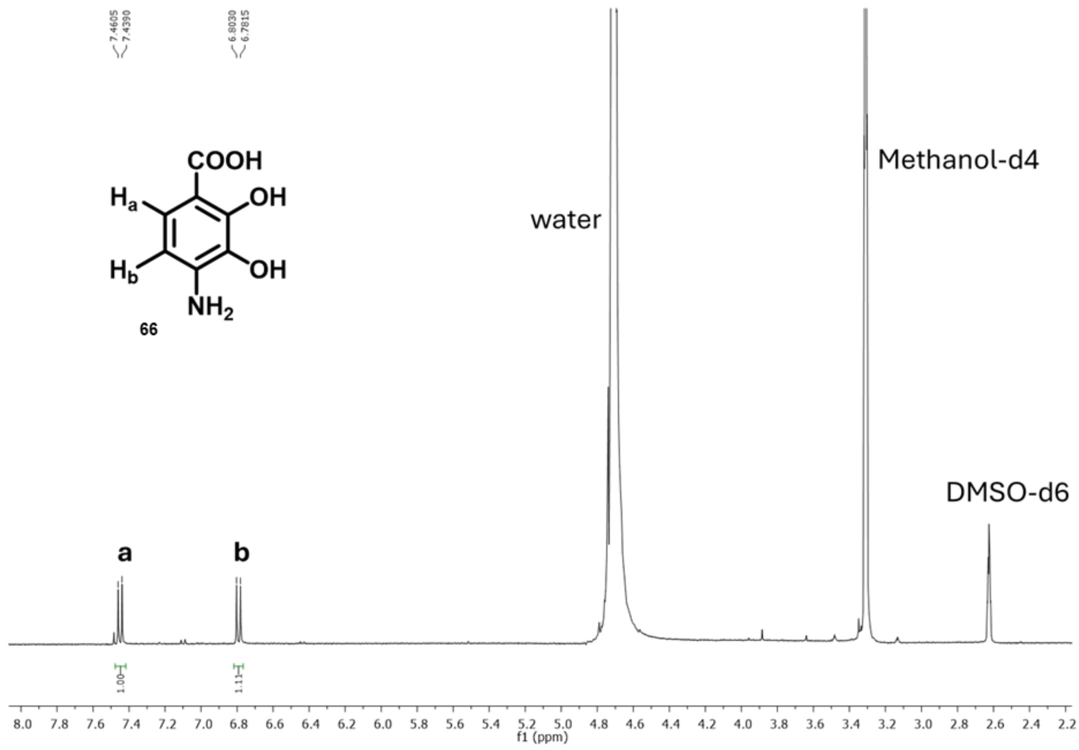

Figure S34. ^{1}H NMR of intermediate **66**.

4-(carboxyformamido)-2,3-dihydroxybenzoic acid (68)

4-amino-2,3-dihydroxybenzoic acid (**66**) (100 mg, 0.59 mmol, 1 eq) was purged in nitrogen and dissolved in dry DMF. Nitrogen was bubbled through the solvent. Methyl chlorooxoacetate (**67**) (66 µL, 0.79 mmol, 1.2 eq) and triethylamine (100 µL, 0.79 mmol, 1.2 eq) were added to the flask while maintaining the inert atmosphere, and then this mixture was stirred at room temperature overnight. Dry DMF was concentrated *in vacuo* to give a sticky precipitate that was washed and sonicated several times with ether and dichloromethane to form a brown powder **(68)**. The triethylamine salt of the product is formed in this reaction as indicated by its NMR. Dissolution of the product in water or buffer hydrolyzes the methyl ester of the N-oxalyl chain, forming the corresponding carboxylic acid **(33b)**.[M-H]$^{-}$: 240.0146. ^{1}H NMR (400 MHz, DMSO-d_6) δ 7.67 (1H, d, J= 8.76 Hz), 7.26 (1H, d, J= 8.76 Hz), 3.10 (2H, q), 1.19 (3H, t).^{13}C NMR (400 MHz, DMSO-d_6) δ: 172.59, 162.70, 160.53, 151.24, 134.25, 131.02, 121.03, 111.16, 108.57, 45.99, 8.96.

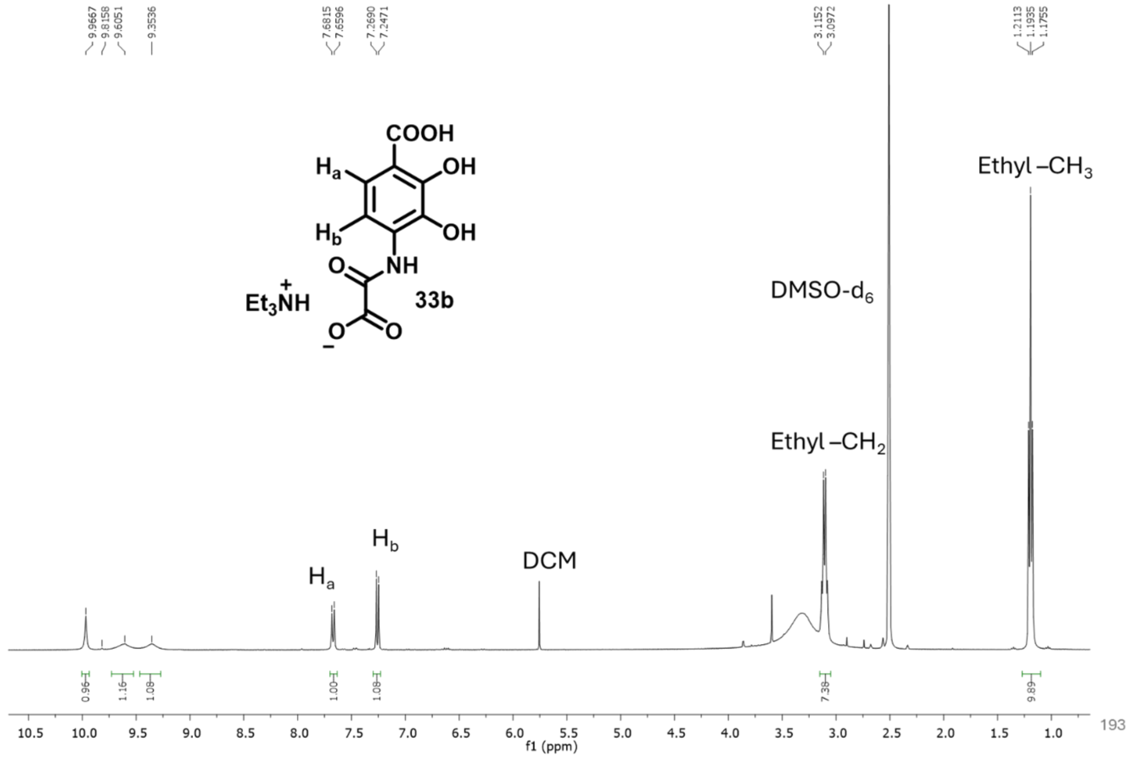

Figure S35. ^{1}H NMR of compound **33b.**

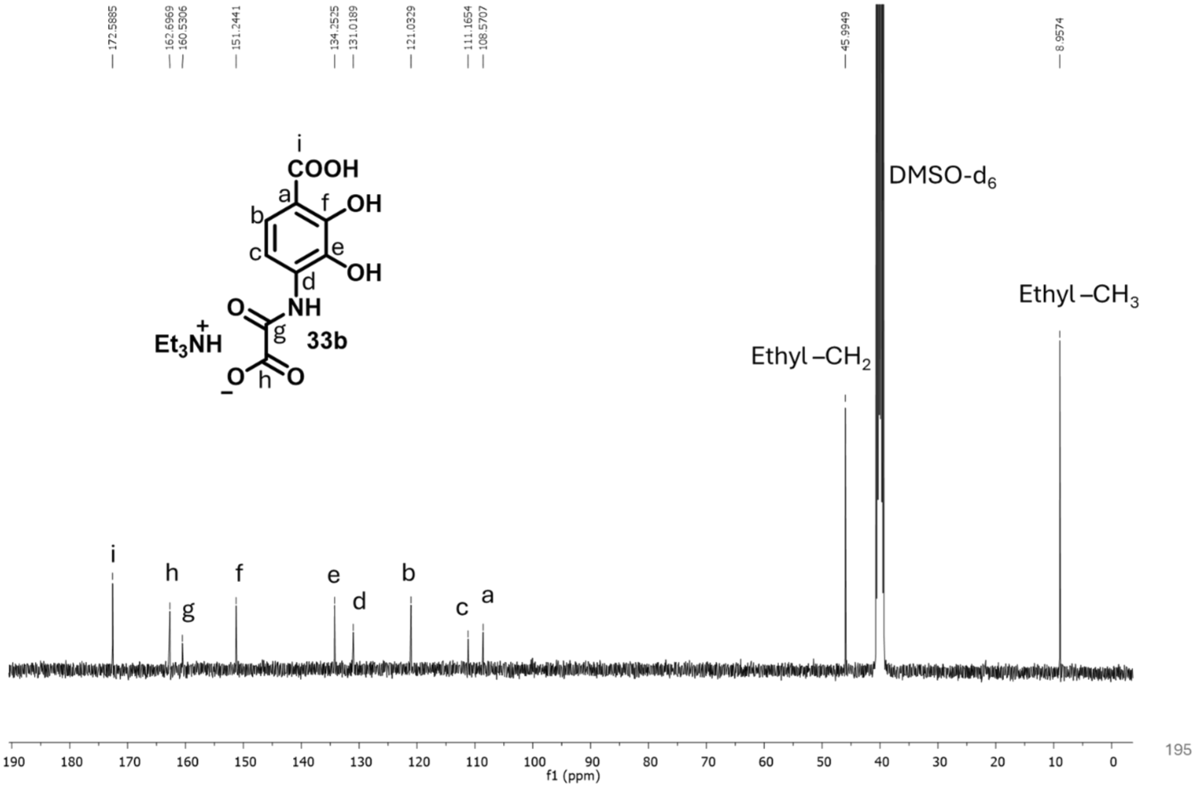

Figure S36. ^{13}C NMR of compound **33b.**

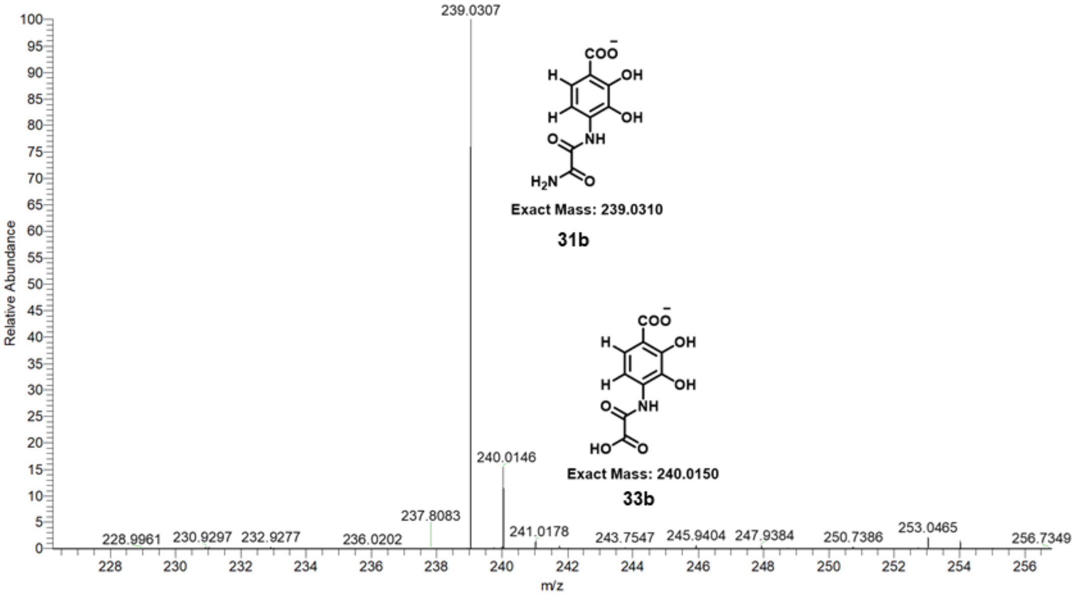

Figure S37. LC-MS data for **31b**. The terminal amide of **31b** is nonenzymatically hydrolyzed to the corresponding carboxylic acid **33b** when in 100 mM potassium phosphate (pH 7.5) for 1 hour.

Synthesis of 4-(carboxyformamido)-2,3-dihydroxy-6-methylbenzoic acid (33a)

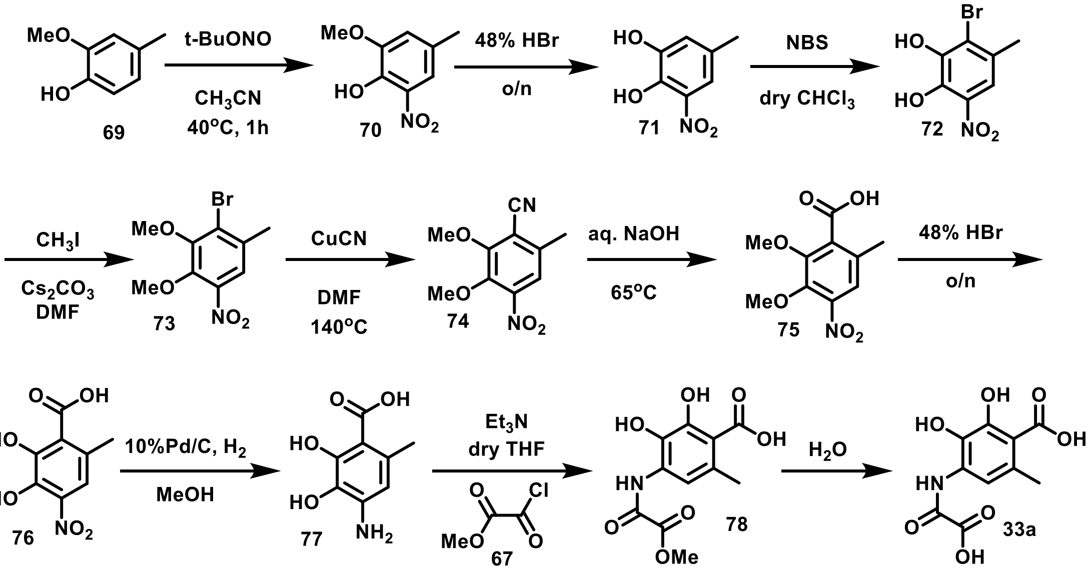

Figure S38. Synthetic scheme for the LumA, LumB, LumC, and LumD product **(33a)**.

2-methoxy-4-methyl-6-nitrophenol (70)

This method is based on a reported procedure for radical nitration using t-butyl nitrite[3]. Creosol (**69**) (5 mL, 36.2 mmol) was dissolved in acetonitrile with tert-butyl nitrite (7.5 g, 72.4 mmol) and stirred at 40 °C with the reaction being exposed to air. The starting material was consumed within 1 h. The solvent was concentrated, and the crude was further purified by loading the residue onto a bed of silica in a Buchner funnel and washing it with 5-10% ethyl acetate-hexane. The washes containing the product were concentrated to yield an orange solid (60% yield). ^{1}H NMR (400 MHz, $CDCl_3$) δ 10.58 (1H, s), 7.47 (1H, d, J=0.6 Hz), 6.94 (1H, d, J= 1.2 Hz), 3.92 (3H, s), 2.33 (3H, s).

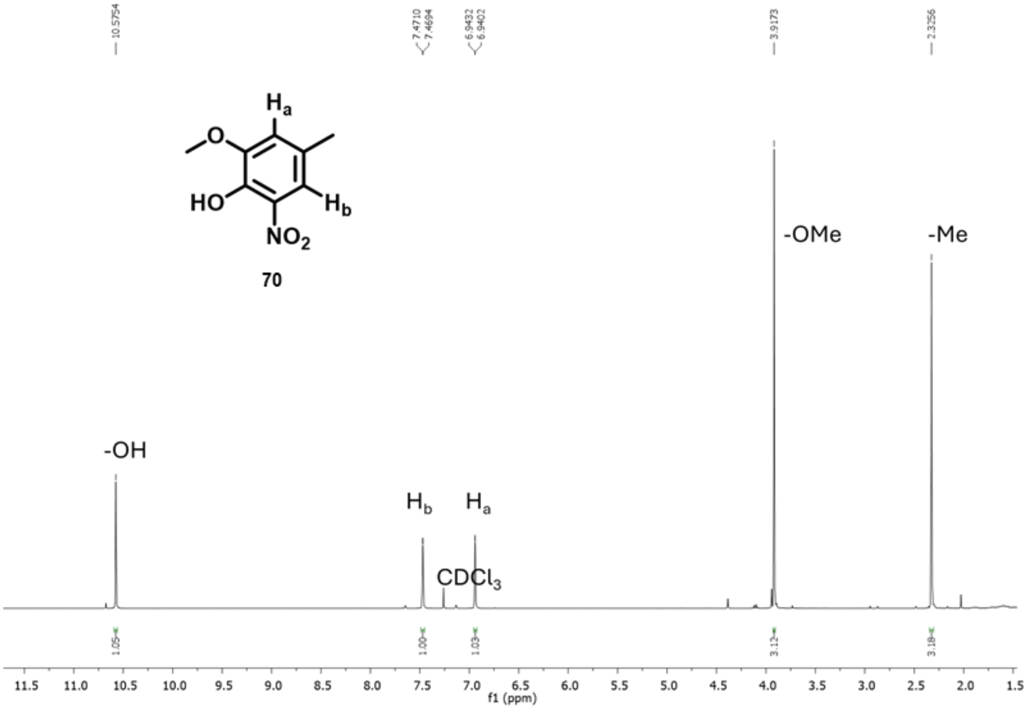

Figure S39. ^{1}H NMR of intermediate **70**.

5-methyl-3-nitrobenzene-1,2-diol (71)

2-methoxy-4-methyl-6-nitrophenol (**70**) (3.8 g, 20.7 mmol) was added to 48% HBr (forms a suspension) and the reaction was refluxed overnight. The mixture was then extracted with ethylactetate and water. The ethylacetate layer was concentrated *in vacuo* to obtain a brown liquid. To obtain the pure product, the crude mixture was loaded onto a silica bed in a glass Buchner funnel. This was washed repeatedly with 10-20% ethylacetate-hexane and the collections were monitored by TLC. Washes containing the product were concentrated to yield pure demethylated product as an orange solid (75% yield). ^{1}H NMR (400 MHz, $CDCl_3$) δ 10.44 (s, 1H), 7.44 (s, 1H), 7.07 (d, J = 1.5 Hz, 1H), 2.31 (s, 3H).

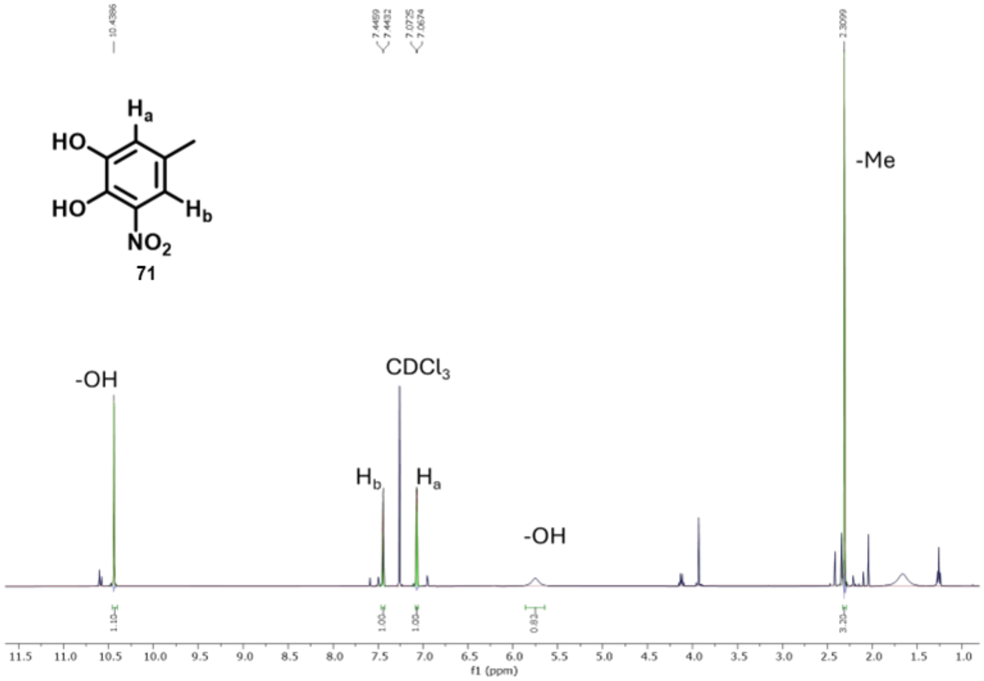

Figure S40. ^{1}H NMR of intermediate **71.**

3-bromo-4-methyl-6-nitrobenzene-1,2-diol (72)

5-methyl-3-nitrobenzene-1,2-diol (**71**) (1.6 g, 9.6 mmol) and NBS (2.1 g, 11.6 mmol) were dissolved in dry chloroform. This was stirred for 24 h at room temperature while being protected from light. This was followed by a workup with water and chloroform. Chloroform layer was dried with sodium sulfate and concentrated *in vacuo*. This crude mixture was used for the next step without further purification (60% yield).

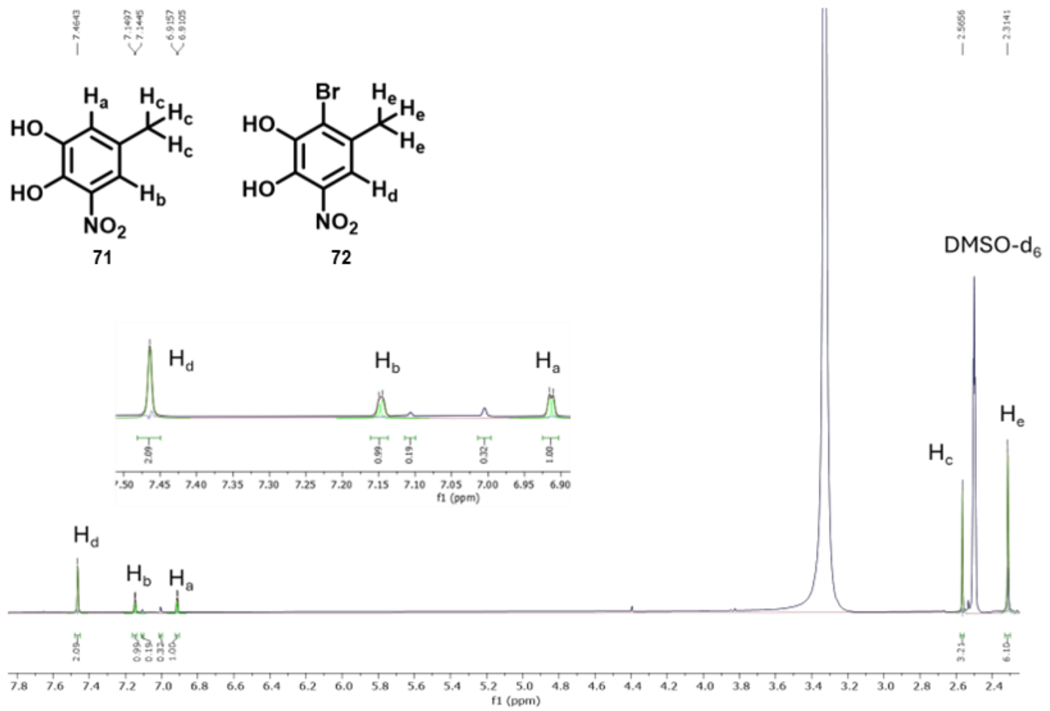

Figure S41. ^{1}H NMR of intermediate **72**. Inset shows the aromatic region between 6.90 and 7.50 ppm.

2-bromo-3,4-dimethoxy-1-methyl-5-nitrobenzene (73)

The product mixture from the previous step was mixed with 7 eq of Cs_2CO_3 in dry DMF. Iodomethane (4 eq) was added under inert conditions followed by heating at 70 °C overnight. To remove cesium carbonate, the reaction mixture was washed with ethylacetate through a plug of celite. DMF was concentrated *in vacuo* and extraction was done using water and ethylacetate. Ethylacetate layer was dried with sodium sulfate and concentrated to obtain the crude which was purified by column chromatography (1:50 EtOAc-hexane). ^{1}H NMR (400 MHz, $CDCl_3$) δ 7.48 (s, 1H), 3.99 (s, 3H), 3.92 (s, 14H), 2.43 (s, 3H).

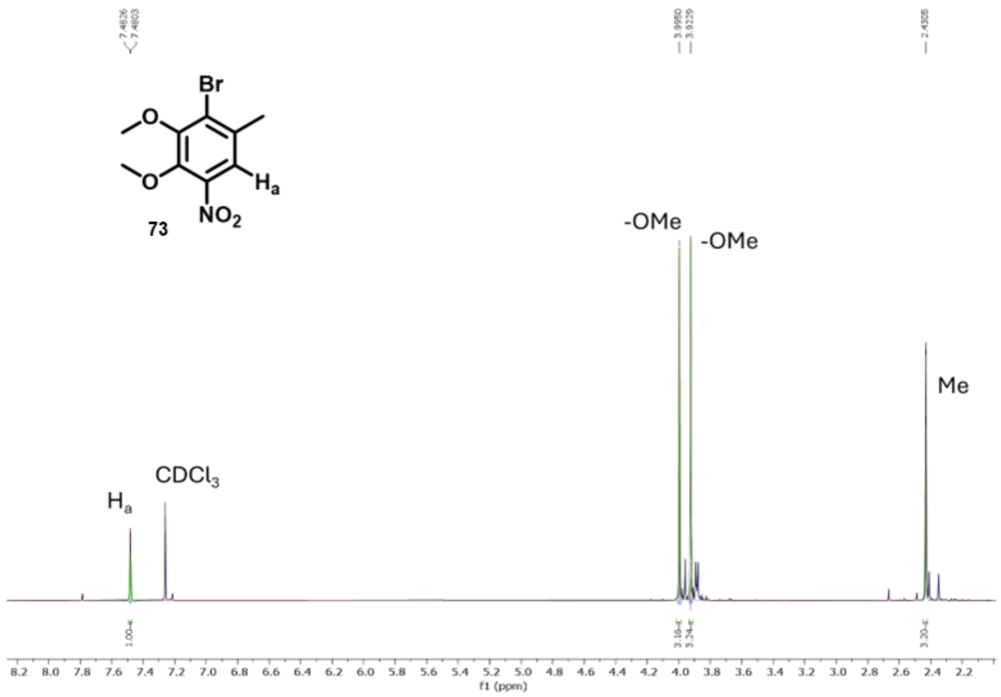

Figure S42. ^{1}H NMR of intermediate **73.**

2,3-dimethoxy-6-methyl-4-nitrobenzonitrile (74)

2-bromo-3,4-, dimethoxy-1-methyl-5-nitrobenzene (**73**) (900 mg, 3.3 mmol) and Cu(I)CN (352 mg, 3.9 mmol) was mixed in dry DMF under inert conditions. The mixture was heated to 140°C and stirred for 16-18 h. The solvent was concentrated *in vacuo*. A few drops of 6 M HCl were added during the workup with water and ethylacetate. Ethylacetate layer was dried with sodium sulfate and evaporated to obtain the crude. Silica column chromatography was performed to isolate the product as yellow crystals (1:20 EtOAc-hexane, 40% yield). ^{1}H NMR (400 MHz, $CDCl_3$) δ 7.35 (s, 1H), 4.10 (s, 3H), 3.98 (s, 3H), 2.53 (s, 3H).

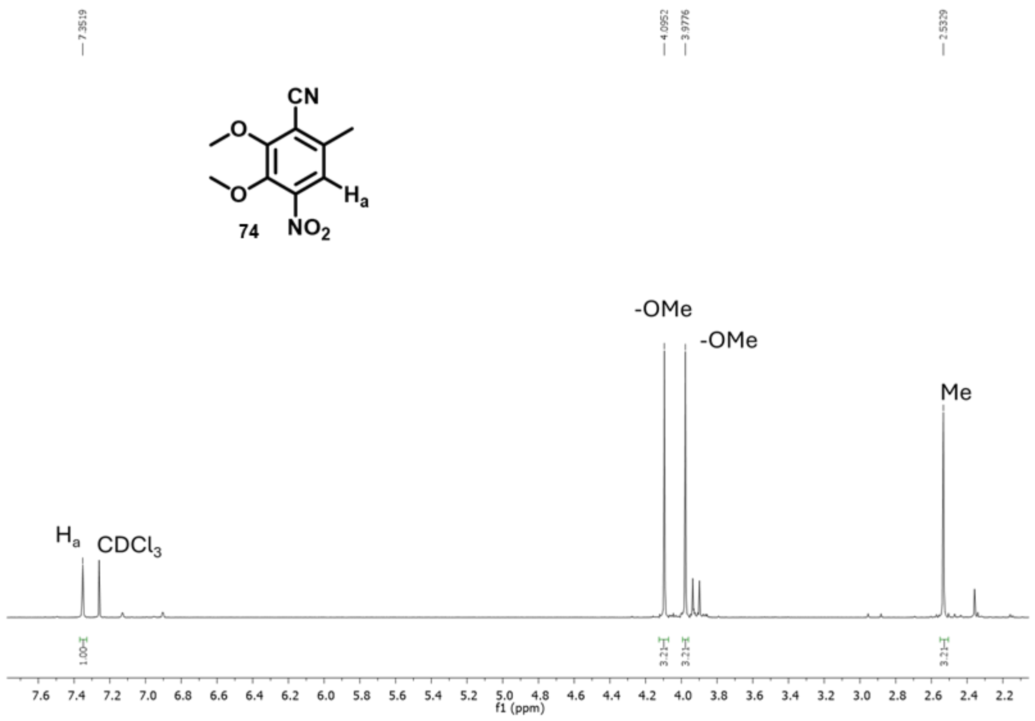

Figure S43. ^{1}H NMR of intermediate **74**.

2,3-dimethoxy-6-methyl-4-nitrobenzoic acid (75)

270 mg of 2,3-dimethoxy-6-methyl-4-nitrobenzonitrile (**71**) was dissolved in 50 mL of 0.5 N NaOH and stirred overnight at 65 °C. The reaction was diluted with water and the product was extracted using ethylacetate. The ethylacetate layer was dried using sodium sulfate and concentrated *in vacuo* to obtain 2,3-dimethoxy-6-methyl-4-nitrobenzoic acid (**75**) as a yellow solid (70% yield).

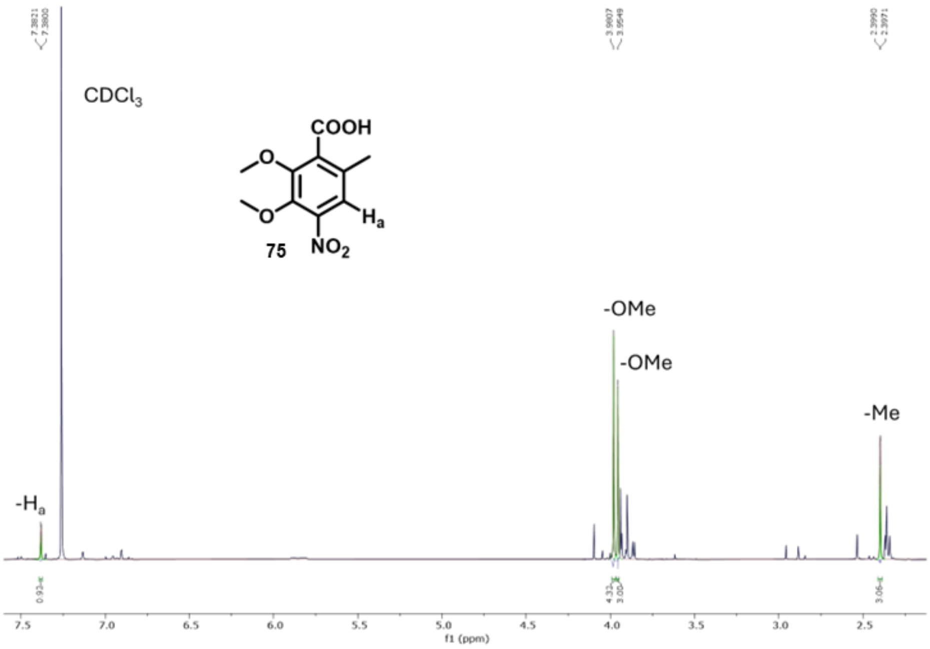

Figure S44. ^{1}H NMR of intermediate **75.**

2,3-dihydroxy-6-methyl-4-nitrobenzoic acid (76)

The reaction was set-up and work-up was done according to the procedure outlined above for the demethylation of 5-methyl-3-nitrobenzene-1,2-diol. ^{1}H NMR (400 MHz, MeOD) δ 7.45 (s, 1H), 2.30 (s, 3H).

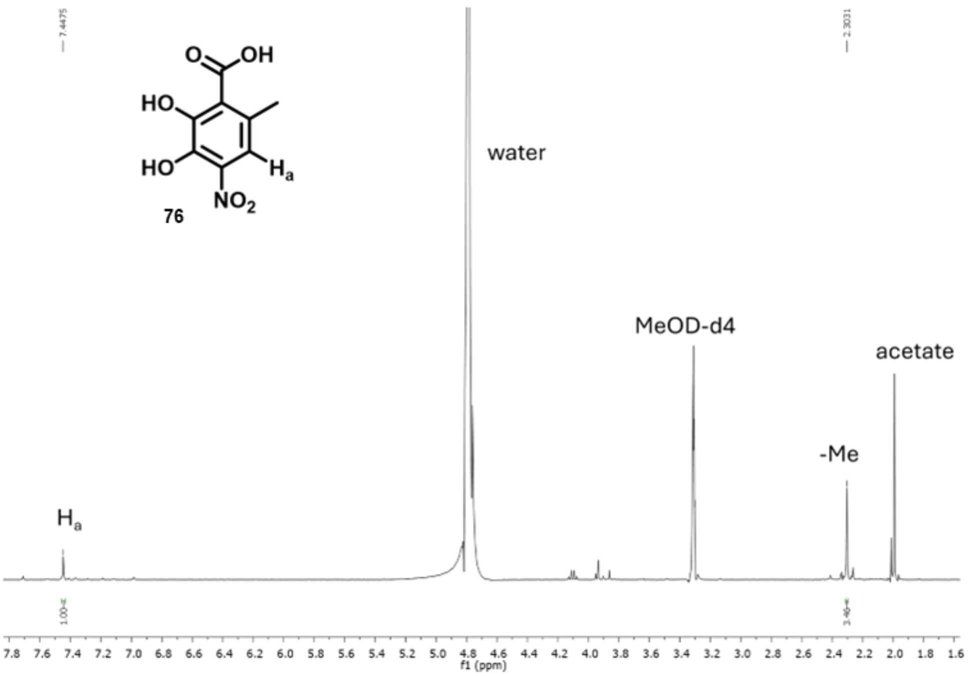

Figure S45. ^{1}H NMR of intermediate **76.**

4-amino-2,3-dihydroxy-6-methylbenzoic acid (77)

100 mg of 2,3-dimethoxy-6-methyl-4-nitrobenzoic acid (**76**) and 50 mg of 10% Pd/C were dissolved in ethylacetate. This was purged with hydrogen three times and set up for overnight reduction in a Parr reactor vessel connected to a hydrogen cylinder. The reaction was transferred to a 50 mL centrifuge tube and subjected to centrifugation. The Pd/C settled at the bottom of the tube, and the supernatant was removed and concentrated. The Pd/C residue was washed several times with ethylacetate and methanol until no further spots were visible by TLC. This was purified by silica column chromatography (1:10 MeOH:$CHCl_3$) to isolate the pure reduction product (30% yield). $[M-H]^-$: 182.0450. ^{1}H NMR (400 MHz, MeOD) δ 6.14 (s, 1H), 2.37 (s, 3H).

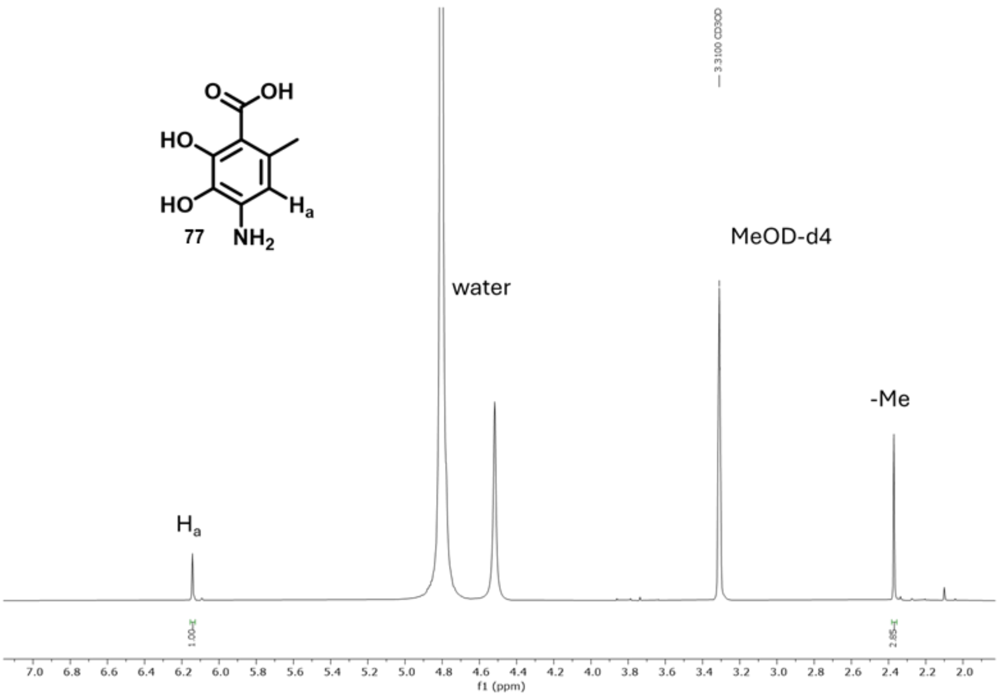

Figure S46. ^{1}H NMR of intermediate 77.

4-(carboxyformamido)-2,3-dihydroxy-6-methylbenzoic acid (33a)

4-amino-2,3-dihydroxy-6-methylbenzoic acid (**77**) (20 mg, 0.1 mmol) was mixed with methyl chloro-oxoacetate (**67**) (18.4 mg, 0.15 mmol) and triethylamine (56 µL, 0.4 mmol) in dry DMF under inert atmosphere. This was stirred overnight at 37 °C. Then DMF was concentrated *in vacuo*. The residue was washed with dichloromethane and hexanes to obtain a light brown solid. The presence of 2,3-dihydroxy-4-(2-methoxy-2-oxoacetamido)-6-methylbenzoic acid (**78**) in the mixture was confirmed by LC-MS. The methyl ester gets hydrolyzed to the carboxylic acid when dissolved in water. This mixture was purified by HPLC to isolate the pure carboxylic acid product (**33a**). $[M-H]^-$: 254.0304. ^{1}H NMR (400 MHz, D_2O_salt) δ 7.23 (s, 1H), 2.59 (s, 3H).

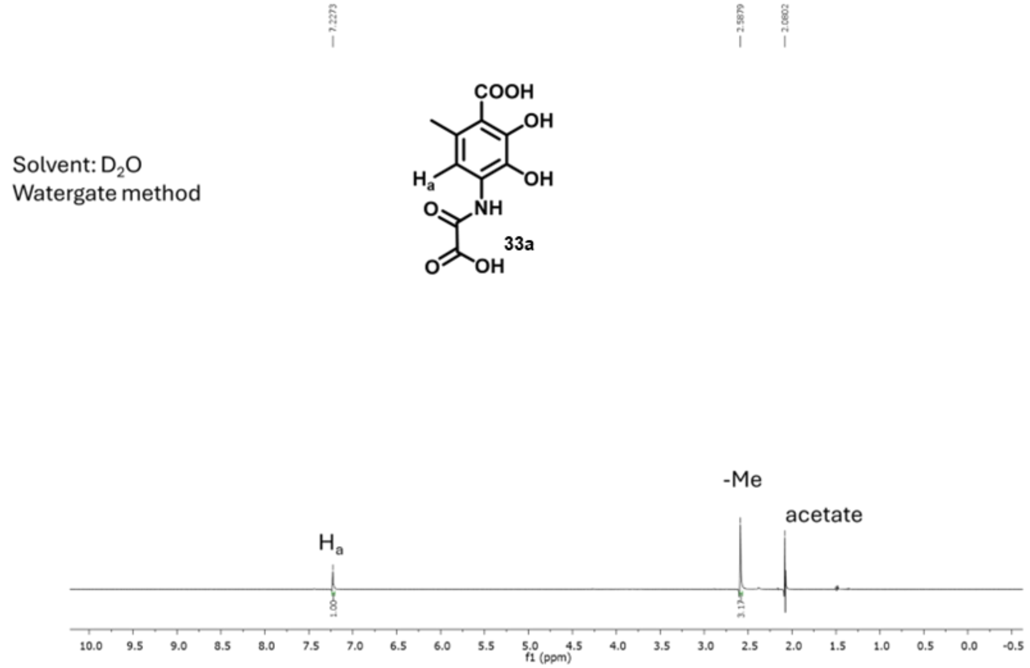

Figure S47. ^{1}H NMR of compound **33a.**

Characterization of LumA, LumB, LumC, and LumD

Overexpression and Purification of LumB, LumA, LumC and LumD

The dioxygenase comprises two subunits: LumC, the α subunit that contains the [2Fe-2S], and LumD, the β subunit. In the gene cluster, *lumC* and *lumD* occur alongside a partner ferredoxin (*lumB*) that contains [3Fe-4S] and [4Fe-4S] clusters and an NADPH-dependent, flavin-containing ferredoxin reductase (*lumA*), which facilitates electron transfer to the oxygenating subunits. All of these subunits were overexpressed in separate plasmids and mixed together for activity assays.

Overexpression and purification LumC (α-subunit):

GenScript synthesized the gene in pETDuet-1 with an N-terminal His-tag. This was transformed into *E.coli* BL21(*DE3*) competent cells. Colonies obtained were grown in a 100 mL culture of LB with ampicillin (100 µg/mL) for 12 h at 37 °C (220 rpm). This culture was used to inoculate 1.5 L of LB media at 37 °C. At about OD_{600} of 0.2, 100 mg iron(II) ammonium sulfate and 100 mg L-cysteine were added to 1.5 L of culture. When OD_{600} of 0.6 was reached, 0.5 mM of IPTG was added and the cells were grown for 18 h at 15 °C (120 rpm). This was followed by centrifugation to harvest the cells for 15 min at 5000 rpm and the cells were stored in liquid nitrogen. Typical yields were 15 g of cells (wet weight) from 4.5 L of cell culture.

For purification, harvested cells were thawed and resuspended in 65 mL phosphate lysis buffer (100 mM KPi, 150 mM NaCl, pH 7.5). Lysozyme (30 mg) and benzonase nuclease (3 µL) were added to this and the suspension was stirred in an ice bath for 30 min. Cells were lysed by sonication and the mixture subjected to centrifugation at 15,000 rpm for 40 min to remove the cell

debris. The supernatant was filtered using 0.22 μm filters and loaded onto a Ni-NTA His-trap column pre-equilibrated in lysis buffer. The column was then washed with 100 mL of wash buffer (100 mM KPi, 20 mM imidazole, 150 mM NaCl, pH 7.5) and 50 mL of the same buffer with increased imidazole concentration (70 mM). To elute protein, elution buffer (100 mM KPi, 250 mM imidazole, 150 mM NaCl, pH 7.5) was run through the column and fractions of the elute were collected. The fractions were tested with Bradford reagent and the ones containing the protein were pooled and concentrated using 15 mL Amicon Ultra 10 kDa filters. The buffer of the concentrated protein was exchanged inside an anaerobic chamber using Cytiva PD-10 desalting columns to 100 mM KPi, 30% glycerol, pH 7.5. The desalted enzyme was pipetted to make aliquots and flash-frozen with liquid nitrogen, and stored at -80 °C. Protein concentration was determined using the absorbance at 280 nm (A_{280}), and the extinction coefficient calculated by the ProtParam tool of the ExPASy proteomics server (ε_{280} = 74370 M^{-1} cm^{-1})

Overexpression and purification of LumD (β-subunit):

The gene cloned in pTHT was synthesized by GenScript. This was transformed into *E.coli* BL21(*DE3*) competent cells. Colonies obtained were grown in a 100 mL culture of LB with kanamycin (40 μg/mL) for 12 h at 37 °C (220 rpm). This culture was used to inoculate 1.5 L of LB media at 37 °C. When OD_{600} of 0.6 was reached, 0.5 mM of IPTG was added and the cells were grown for 18 h at 15 °C (180 rpm). This was followed by centrifugation to harvest the cells for 15 min at 5000 rpm and the cells were stored in liquid nitrogen. Typical yields were 12 g of cells (wet weight) from 4.5 L of cell culture.

For purification, harvested cells were thawed and resuspended in 65 mL phosphate lysis buffer (100 mM KPi, 150 mM NaCl, pH 7.5). Lysozyme (30 mg) and benzonase nuclease (2 μL) were added to this and the suspension was stirred in an ice bath for 30 min. Cells were lysed by sonication and the mixture subjected to centrifugation at 15,000 rpm for 40 min to remove the cell debris. The supernatant was filtered using 0.22 μm filters and loaded onto a Ni-NTA His-trap column pre-equilibrated in lysis buffer. The column was then washed with 100 mL of wash buffer (100 mM KPi, 20 mM imidazole, 150 mM NaCl, pH 7.5) and 50 mL of the same buffer with increased imidazole concentration (70 mM). To elute protein, elution buffer (100 mM KPi, 250 mM imidazole, 150 mM NaCl, pH 7.5) was run through the column, and fractions of the elute were collected. The fractions were tested with Bradford reagent, and the ones containing the protein were pooled and concentrated using 15 mL Amicon Ultra 10 kDa filters. The buffer of the concentrated protein was exchanged using Cytiva PD-10 desalting columns to 100 mM KPi, 30% glycerol, pH 7.5. The desalted enzyme was pipetted to make aliquots and flash-frozen with liquid nitrogen and stored at -80 °C. Protein concentration was determined using the absorbance at 280 nm (A_{280}) and the extinction coefficient calculated by the ProtParam tool of the ExPASy proteomics server (ε_{280} = 35410 M^{-1} cm^{-1}).

Overexpression and purification of LumB (Ferredoxin)

The gene cloned in pTHT was synthesized by GenScript. This was transformed into *E.coli* pSUF BL21(*DE3*) competent cells. Colonies obtained were grown in a 100 mL culture of LB with kanamycin (40 μg/mL) and chloramphenicol (34 μg/mL) for 12 h at 37 °C (220 rpm). This culture

was used to inoculate 1.5 L of LB media at 37 °C. At about OD_{600} of 0.2, 100 mg iron(II) ammonium sulfate and 100 mg L-cysteine were added to 1.5 L of culture. When OD_{600} of 0.6 was reached, 0.5 mM of IPTG was added, and the cells were grown for 18 h at 15 °C (120 rpm). This was followed by centrifugation to harvest the cells for 15 min at 5000 rpm, and the cells were stored in liquid nitrogen. Typical yields were 10 g of cells (wet weight) from 6 L of cell culture.

For purification, harvested cells were thawed and resuspended in 65 mL phosphate lysis buffer (100 mM KPi, 150 mM NaCl, pH 7.5). Lysozyme (30 mg) and benzonase nuclease (2 µL) were added to this and the suspension was stirred in an ice bath for 30 min. Cells were lysed by sonication, and the mixture was subjected to centrifugation at 15,000 rpm for 40 min to remove the cell debris. The supernatant was filtered using 0.22 µm filters and loaded onto a Ni-NTA His-trap column pre-equilibrated in lysis buffer. The column was then washed with 100 mL of wash buffer (100 mM KPi, 20 mM imidazole, 150 mM NaCl, pH 7.5) and 50 mL of the same buffer with increased imidazole concentration (70 mM). To elute protein, elution buffer (100 mM KPi, 250 mM imidazole, 150 mM NaCl, pH 7.5) was run through the column, and fractions of the elute were collected. The fractions were tested with Bradford reagent, and the ones containing the protein were pooled and concentrated using 15 mL 10k Da filters. Desalting was done inside an anaerobic chamber. Using Cytiva PD-10 desalting columns, the buffer of the concentrated protein was exchanged in an anaerobic chamber to 100 mM KPi, 30% glycerol, pH 7.5. The desalted enzyme was pipetted to make aliquots and flash-frozen with liquid nitrogen and stored at -80 °C. Protein concentration was determined using the absorbance at 280 nm (A_{280}), and the extinction coefficient was calculated by the ProtParam tool of the ExPASy proteomics server (ε_{280} = 5960 M^{-1} cm^{-1}).

Overexpression and purification of LumA (Ferredoxin Reductase)

The gene was cloned in pCDFDuet-1 with an N-terminal His-tag by GenScript. This was transformed into *E.coli* BL21(*DE3*) competent cells. Colonies obtained were grown in a 100 mL culture of LB with streptomycin (50 µg/mL) for 12 h at 37 °C (220 rpm). This culture was used to inoculate 1.5 L of LB media at 37 °C. When OD_{600} of 0.6 was reached, 0.5 mM of IPTG was added, and the cells were grown for 18 h at 15 °C (180 rpm). This was followed by centrifugation to harvest the cells for 15 min at 5000 rpm, and the cells were stored in liquid nitrogen. Typical yields were 5 g of cells (wet weight) from 4.5 L of cell culture.

For purification, harvested cells were thawed and resuspended in 65 mL phosphate lysis buffer (100 mM KPi, 150 mM NaCl, pH 7.5). Lysozyme (30 mg) and benzonase nuclease (2 µL) were added to this and the suspension was stirred in an ice bath for 30 min. Cells were lysed by sonication and the mixture subjected to centrifugation at 15,000 rpm for 40 min to remove the cell debris. The supernatant was filtered using 0.22 µm filters and loaded onto a Ni-NTA His-trap column pre-equilibrated in lysis buffer. The column was then washed with 100 mL of wash buffer (100 mM KPi, 20 mM imidazole, 150 mM NaCl, pH 7.5) and 50 mL of the same buffer with increased imidazole concentration (70 mM). To elute protein, elution buffer (100 mM KPi, 250 mM imidazole, 150 mM NaCl, pH 7.5) was run through the column and fractions of the elute were collected. The fractions were tested with Bradford reagent and the ones containing the protein were pooled and concentrated using 15 mL 10 kDa filters. The buffer of the concentrated protein was

exchanged using Cytiva PD-10 desalting columns to 100 mM KPi, 30% glycerol, pH 7.5. The desalted enzyme was pipetted to make aliquots and were flash-frozen with liquid nitrogen and stored at -80 °C. Protein concentration was determined using Bradford Coomassie assay and the extinction coefficient calculated by the ProtParam tool of the ExPASy proteomics server (ε_{280} = 24410 M^{-1} cm^{-1}).

Assay conditions

1 mM of substrate was incubated with 200 µM of LumC, 200 µM of LumD, 100 µM of LumB, 100 µM of LumA, and 2 mM NADPH in 100 mM KPi pH 7.5 buffer overnight at 37 °C. 10 mM glucose-6-phosphate and glucose-6-phosphate dehydrogenase were added to regenerate NADPH in the reaction mixture. Reaction mixture was further supplemented with 2 mM ascorbic acid and 1 mM ferrous ammonium sulfate. These were passed through 10 kDa PES filters to quench the enzymatic reaction for analysis by HPLC and LC-MS.

HPLC conditions

A. Water

B. 100 mM Potassium phosphate buffer, pH 6.6

C. Methanol

HPLC method

(Flow rate: 1 mL/min)

0 min – 100% B, 5 min – 10%A 90%B, 12 min – 48% A 40% B 12% C, 14 min – 50% A 30% B 20% C, 18 min - 30% A 10% B 60%C, 20 min – 100% B, 25 min – 100% B.

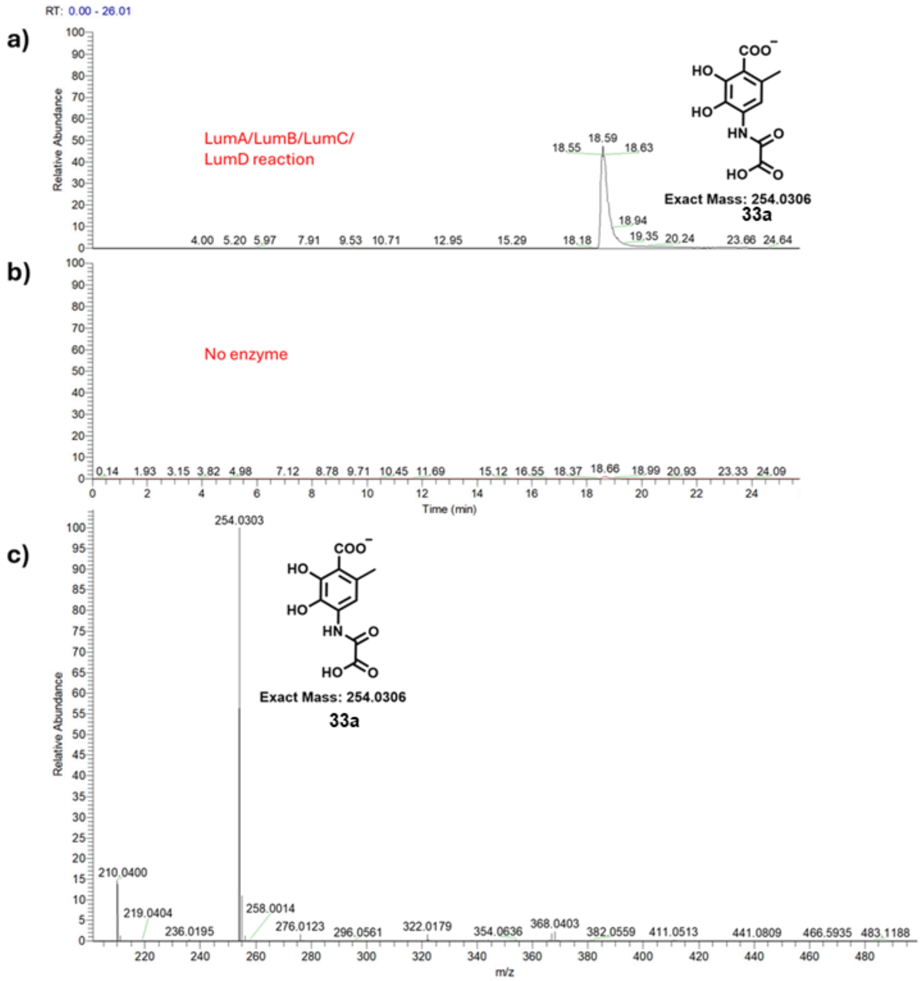

Figure S48. LC-MS data for the LumA, LumB, LumC, and LumD product **33a** with native substrate **30a**. EIC of LumA, LumB, LumC, and LumD product (*m/z* 254.0306 Da) a) in the presence of LumA, LumB, LumC, and LumD and; b) in the absence of LumA, LumB, LumC, and LumD; c) MS of LumA, LumB, LumC, and LumD reaction product showing the *m/z* 254.0303 Da peak.

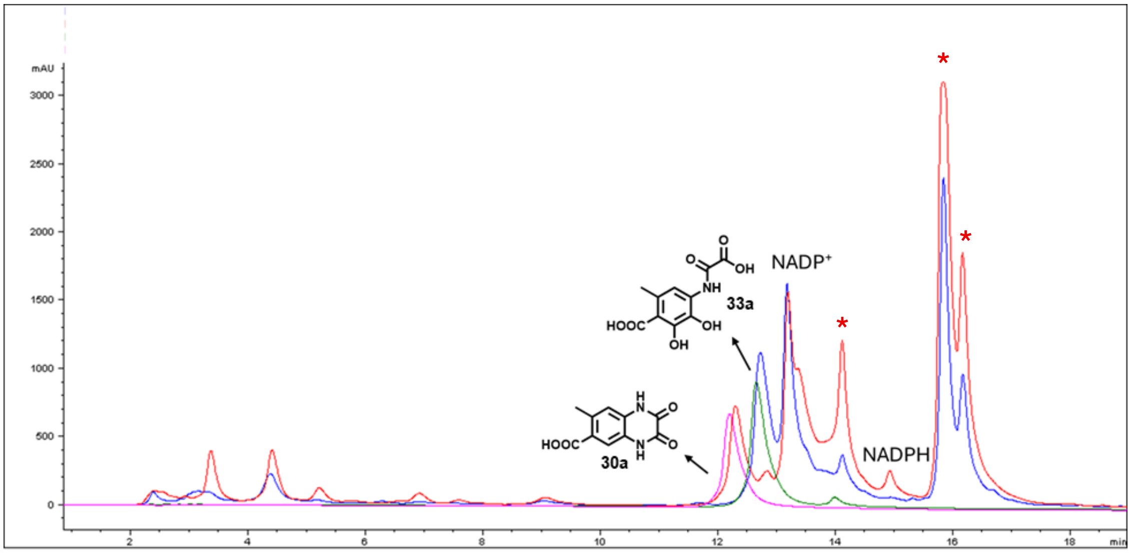

Figure S49. Full chromatogram showing LumA, LumB, LumC, and LumD activity with native substrate (**30a**) (280 nm). Blue trace represents LumA, LumB, LumC, and LumD reaction. Red trace is the no enzyme control. Pink trace is the substrate standard and green trace is the product standard. Red asterisks indicate impurities in the NADP.

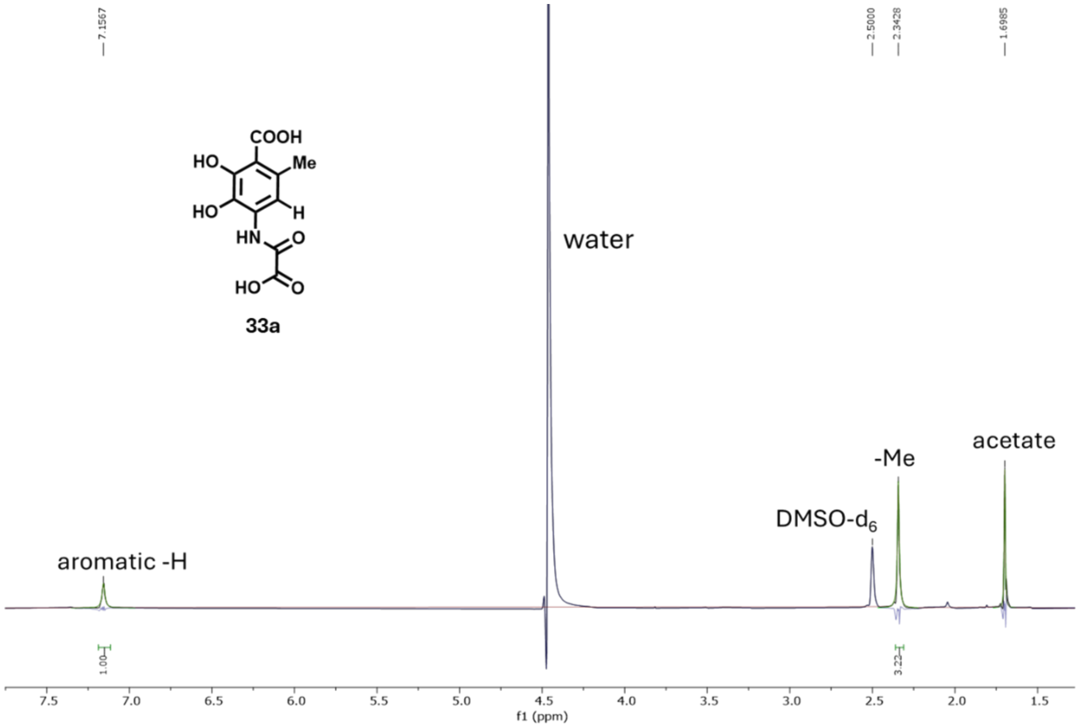

Figure S50. ^{1}H NMR of the product of the LumA, LumB, LumC, and LumD-catalyzed reaction, **33a** (Reference spectrum Figure S47).

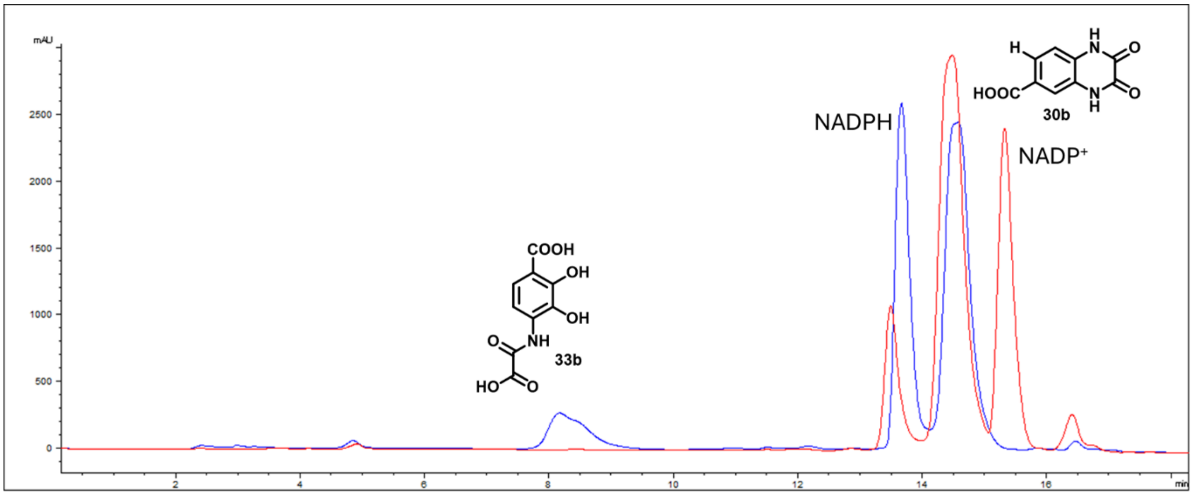

Figure S51. Chromatogram showing LumA, LumB, LumC, and LumD activity with substrate analog **30b** (280 nm). Blue trace represents LumA, LumB, LumC, and LumD reaction; red trace is the no enzyme control.

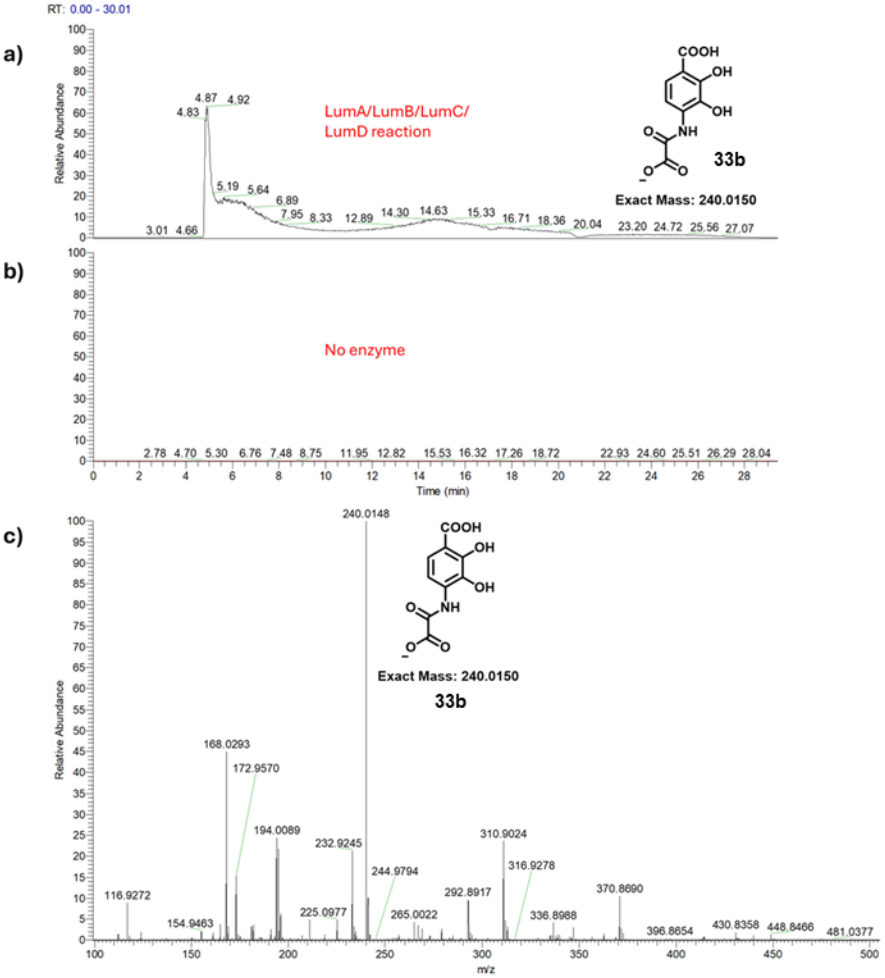

Figure S52. LC-MS data for the LumA, LumB, LumC, and LumD product **33b** with substrate analog **30b**. EIC of LumA, LumB, LumC, and LumD desmethyl product (*m/z* 240.0150 Da) a) in the presence of LumA, LumB, LumC, and LumD and; b) in the absence of LumA, LumB, LumC, and LumD; c) MS of LumA, LumB, LumC, and LumD reaction showing the *m/z* 240.0148 Da peak. The product trailed on the LC column (hydrophilic interaction liquid chromatography column HILIC).

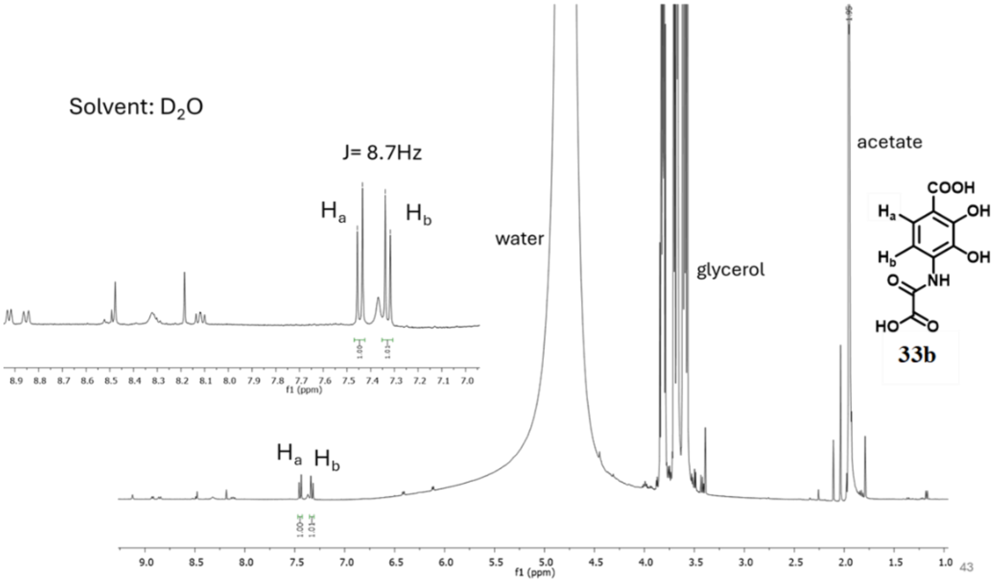

Figure S53. ^{1}H NMR of the product (**33b**) of the LumA, LumB, LumC, and LumD-catalyzed reaction using substrate analog (**30b**). The aromatic peaks have a J value of 8.7 Hz, indicating the product has *o*-coupled protons rather than *m*-coupled protons.

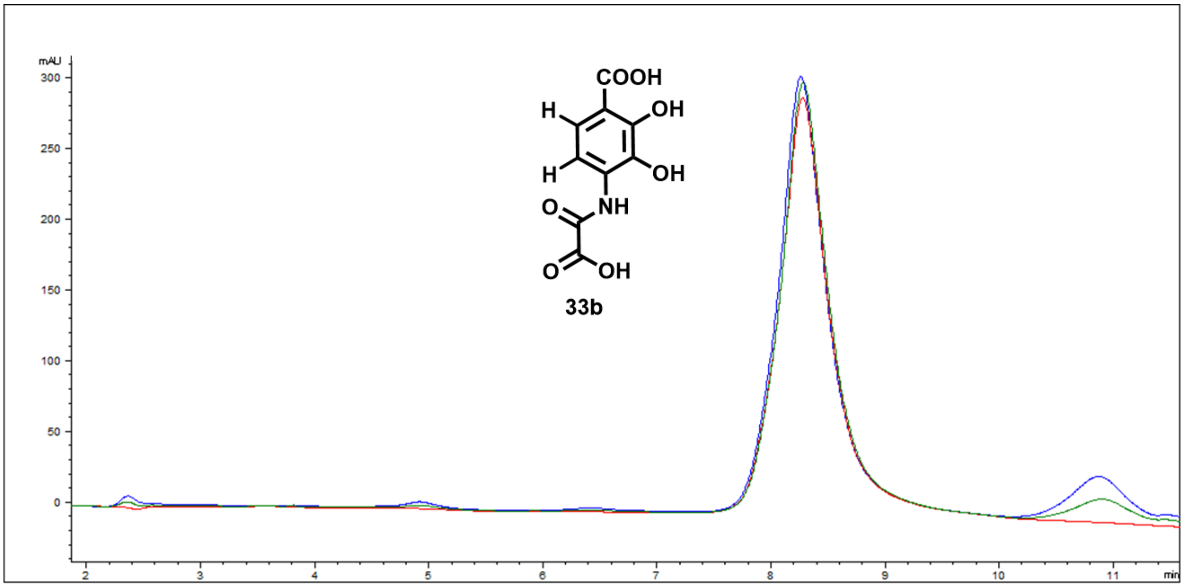

Figure S54. LumA, LumB, LumC, and LumD product with substrate analog **30b** co-elutes with synthetic standard **33b**. Blue trace is the collected product of the LumA, LumB, LumC, and LumD reaction. Red trace is a 1:1 mixture of the collected product and the synthetic standard. Green trace is the synthetic standard. The retention time of **33a** is 3.4 minutes under the same conditions and method.

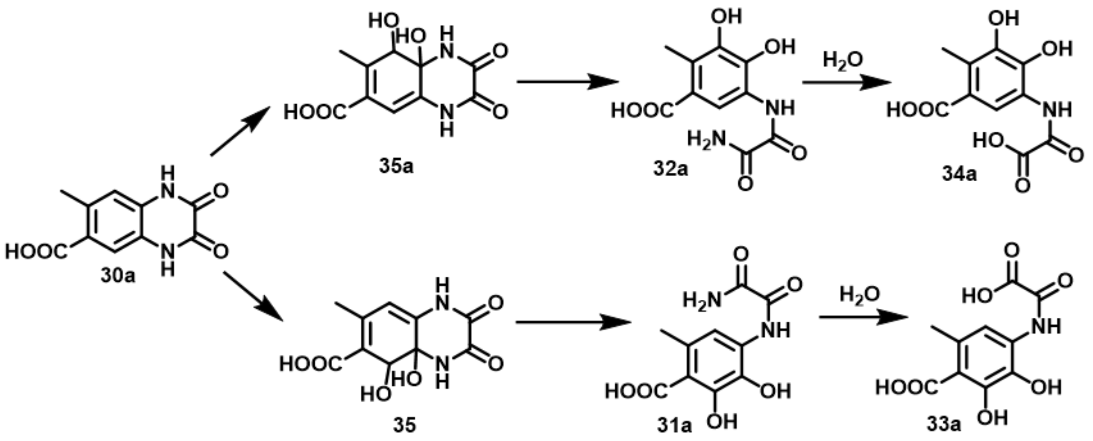

Figure S55. Possible regiosomers from dihydroxylation and heterocyclic ring opening of the substrate (**30a**).

Synthesis of 4-methyl-2-oxo-2H-pyran-6-carboxylic acid (37a)

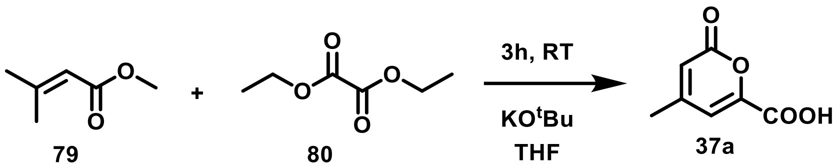

Figure S56. Synthetic scheme of LumE product **(37a)**. For other derivatives of the pyrone (**37**) see Figure S61.

The pyrone carboxylic acid **(37a)** was prepared according to a reported procedure.[4] A suspension of potassium tert-butoxide (197 mg, 1.76 mmol, 2 eq) was prepared in dry THF under argon atmosphere at 0 °C. Ethyl 3,3-dimethylacrylate **(79)** (100 mg, 0.88 mmol, 1 eq) and diethyl oxalate **(80)** (129 mg, 0.88 mmol, 1 eq) were added to this and the mixture was stirred at room temperature for 3 h. This was then extracted with water and the aqueous layer was acidified by H_2SO_4 to pH 4. This was further extracted with ethyl acetate, and both the organic layers (ethyl acetate and THF) were dried over sodium sulfate and concentrated *in vacuo*. The resulting sticky orange precipitate was washed with diethyl ether until the orange color went away giving a light pink colored solid. $[M-H]^+$:155.0335. ^{1}H NMR (400 MHz, DMSO-d_6) δ 7.05 (1H, d, J= 1.44 Hz), 6.41 (1H, m), 2.20 (3H, d, J=1.36 Hz). ^{13}C NMR (400 MHz, DMSO-d_6) δ 160.44, 160.13, 155. 52, 148.22, 116.64, 112.84, 20.58

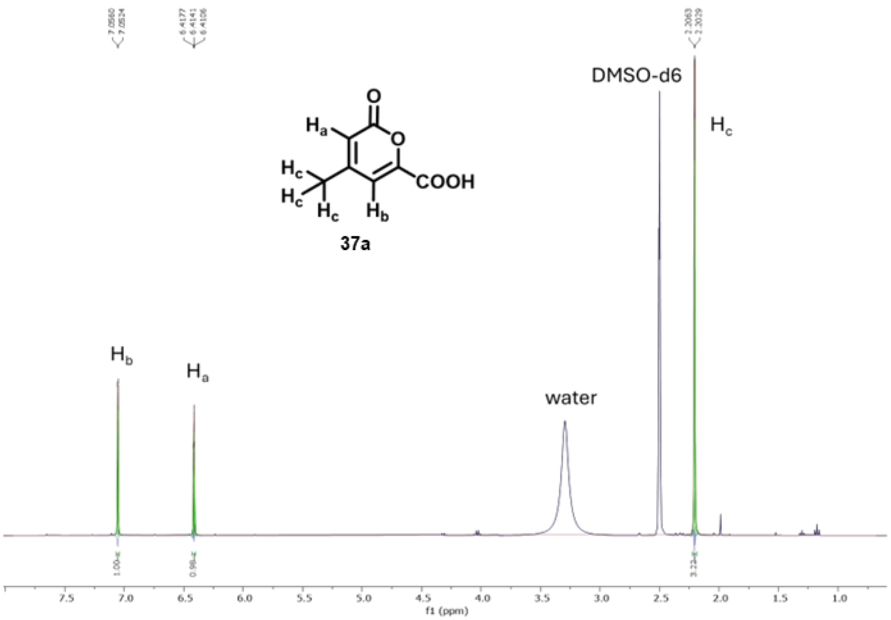

Figure S57. ^{1}H NMR of compound **37a.**

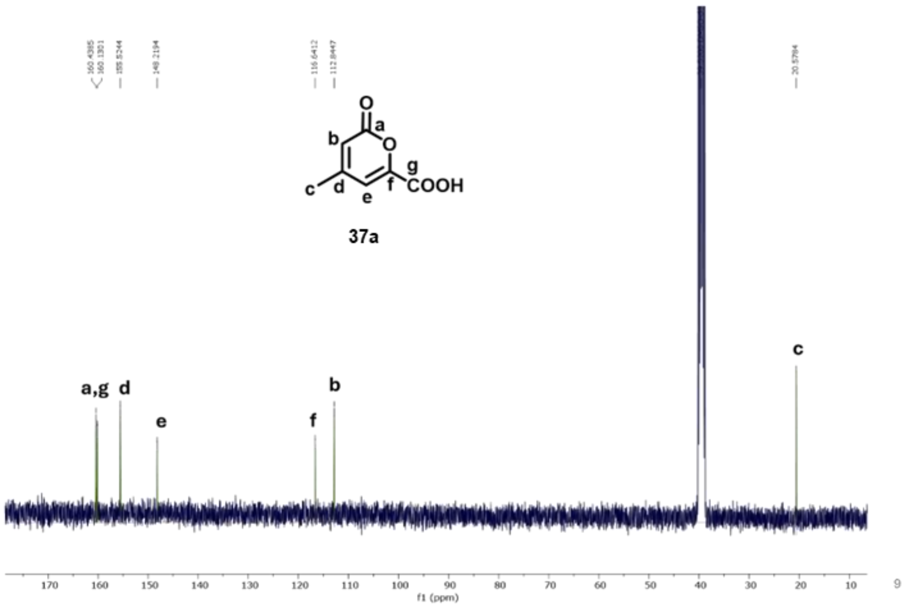

Figure S58. ^{13}C NMR of compound **37a.**

Characterization of LumE

Overexpression and Purification of LumE

The gene cloned in pTHT (a derivative of the pET28b vector with a TEV protease cleavage site after the N-terminal His-tag) was synthesized by GenScript. This was transformed into *E.coli* BL21(DE3) competent cells. Colonies obtained were grown in a 100 mL culture of LB with kanamycin (40 μg/mL) for 12 h at 37 °C (220 rpm). This culture was used to inoculate 1.5 L of

LB media at 37 °C. At about OD_{600} of 0.2, 100 mg iron(II) ammonium sulfate were added to 1.5 L of culture. When OD_{600} of 0.6 was reached, 0.5 mM of IPTG was added and the cells were grown for 18 h at 15 °C (120 rpm). This was followed by centrifugation to harvest the cells for 15 min at 5000 rpm and the cells were stored in liquid nitrogen. Typical yields were 10 g of cells (wet weight) from 3 L of cell culture.

For purification, harvested cells were thawed and resuspended in 65 mL phosphate lysis buffer (100 mM KPi, 150 mM NaCl, pH 7.5). Lysozyme (30 mg) and benzonase nuclease (2 µL) were added to this and the suspension was stirred in an ice bath for 30 min. Cells were lysed by sonication and the mixture subjected to centrifugation at 15,000 rpm for 40 min to remove the cell debris. The supernatant was filtered using 0.22 µm filters and loaded onto a Ni-NTA His-trap column pre-equilibrated in lysis buffer. The column was then washed with 100 mL of wash buffer (100 mM KPi, 20 mM imidazole, 150 mM NaCl, pH 7.5) and 50 mL of the same buffer with increased imidazole concentration (70 mM). To elute protein, elution buffer (100 mM KPi, 250 mM imidazole, 150 mM NaCl, pH 7.5) was run through the column and fractions of the elute were collected. The fractions were tested with Bradford reagent and the ones containing the protein were pooled and concentrated using 15 mL 10 kDa filters. The buffer of the concentrated protein was exchanged using Cytiva PD-10 desalting columns to 100 mM KPi, 30% glycerol, pH 7.5. The desalted enzyme was pipetted to make aliquots and flash-frozen with liquid nitrogen and stored at -80 °C. Protein concentration was determined using the absorbance at 280 nm (A_{280}) and the extinction coefficient calculated by the ProtParam tool of the ExPASy proteomics server (ε_{280}= 51340 M^{-1} cm^{-1}).

Assay conditions

0.2 mM of substrate was incubated with 100 uM enzyme in 100 Kpi buffer (pH 7.5) with 150 µM iron(II) ammonium sulfate for 1 h at 37 °C. The mixture was passed through 10 kDa PES filters to quench the enzymatic reaction and then analyzed by HPLC. For NMR studies, the quenched reaction was vortexed with chelex resin to remove residual Fe(II).

HPLC conditions

A. Water

B. 100 mM Potassium phosphate buffer, pH 6.6

C. Methanol

HPLC method

(Flow rate: 1 mL/min)

0 min – 100% B, 5 min – 10%A 90%B, 12 min – 48% A 40% B 12% C, 14 min – 50% A 30% B 20% C, 18 min - 30% A 10% B 60%C, 20 min – 100% B, 25 min – 100% B.

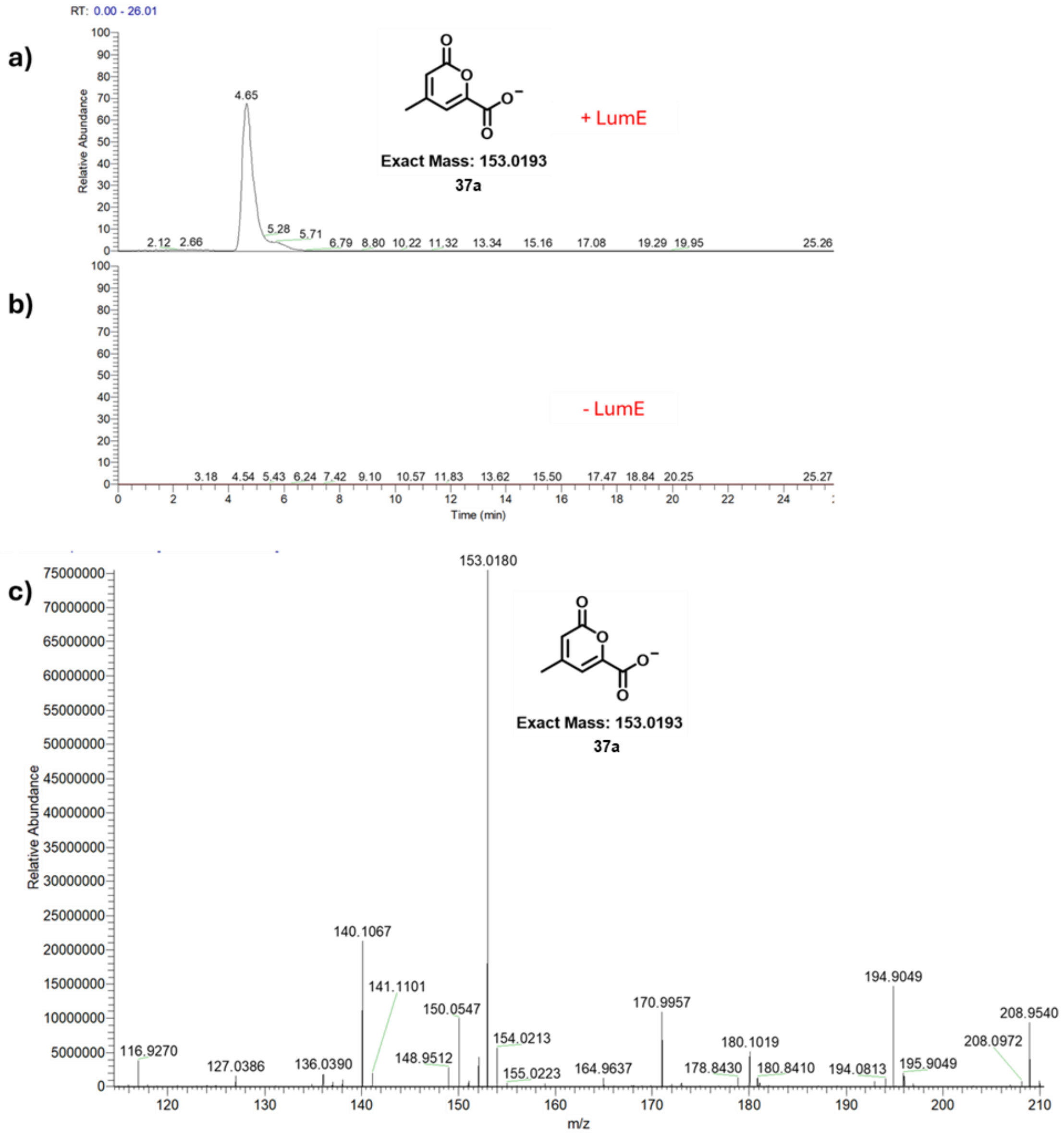

Figure S59. LC-MS data for the LumE product **37a**. EIC of LumE product (*m/z* 153.0193 Da) a) in the presence of LumE and; b) in the absence of LumE; c) MS of the LumE reaction showing the *m/z* 153.0180 Da peak.

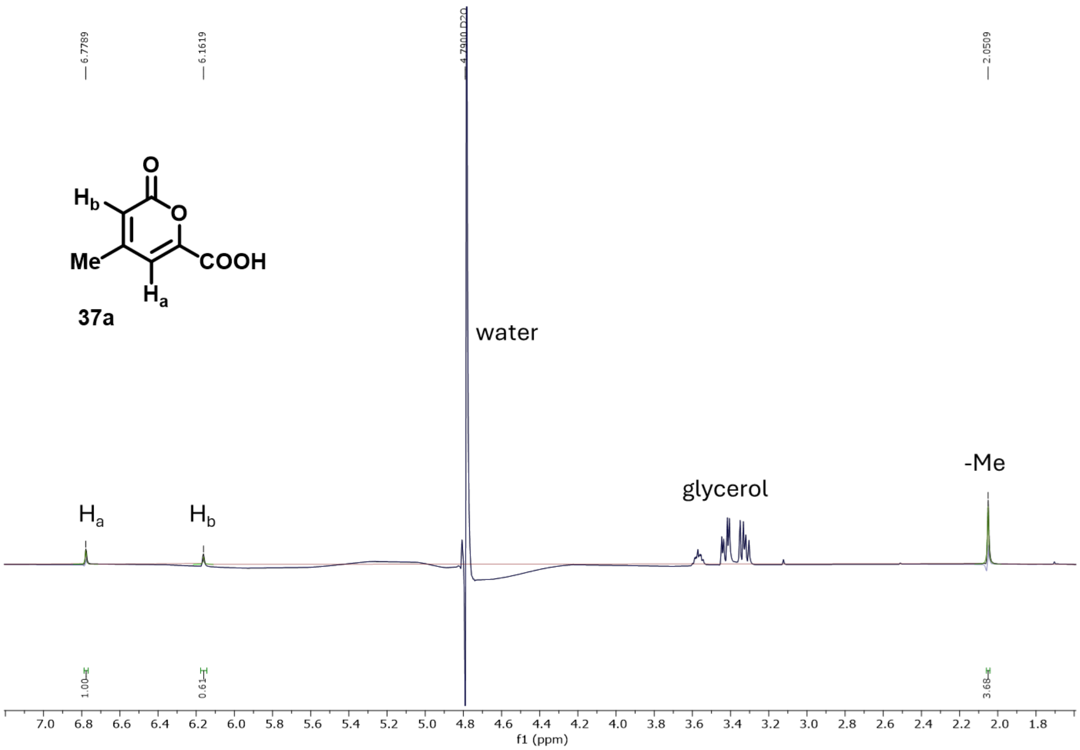

Figure S60. ^{1}H NMR of the product of the LumE-catalyzed reaction, **37a** (Reference spectrum Figure S57).

The substrate analog (**33c**) provided the same pyrone product (**37a**) on incubation with LumE, and that helped us define the steps leading to the pyrone formation. Cyclization to pyrone depends on the substituents present in the linear molecule, and a -H instead of methyl (**33b**) leads to a lower ratio of the pyrone product to the linear molecule.[5] It is plausible that the presence of the methyl group restricts unwanted isomerization in the linear molecule, forming higher amounts of the lactone-forming tautomer. Additionally, spontaneous cyclization of the linear product in solution provides a good strategy for eliminating the amide side chain during catabolism, removing the need for another hydrolase to do this chemistry.

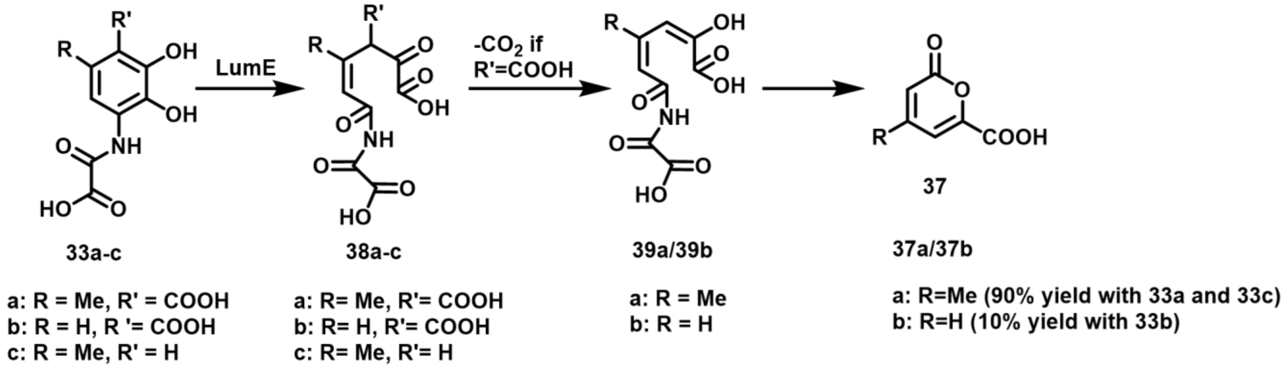

Figure S61. LumE-catalyzed reaction with **33a-c**. Yield of pyrone product **37a** is 90% with both substrates **33a** and **33c**. Yield of pyrone product **37b** is 10% with **33b**.

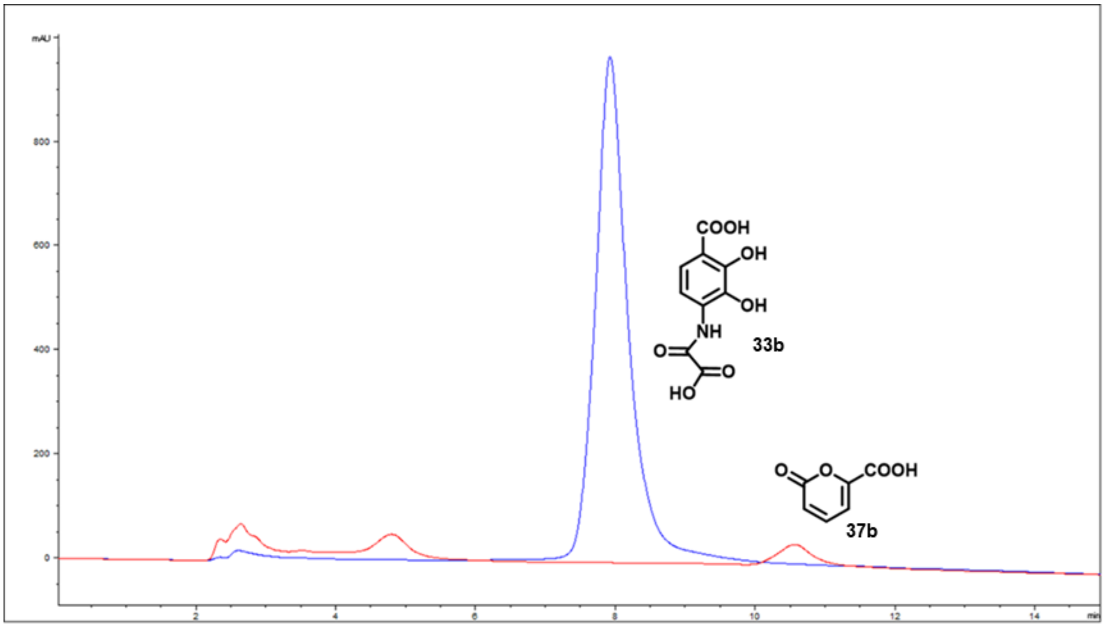

Figure S62. LumE activity with substrate analog **33b**. Red trace represents LumE activity, and blue trace is the no enzyme control.

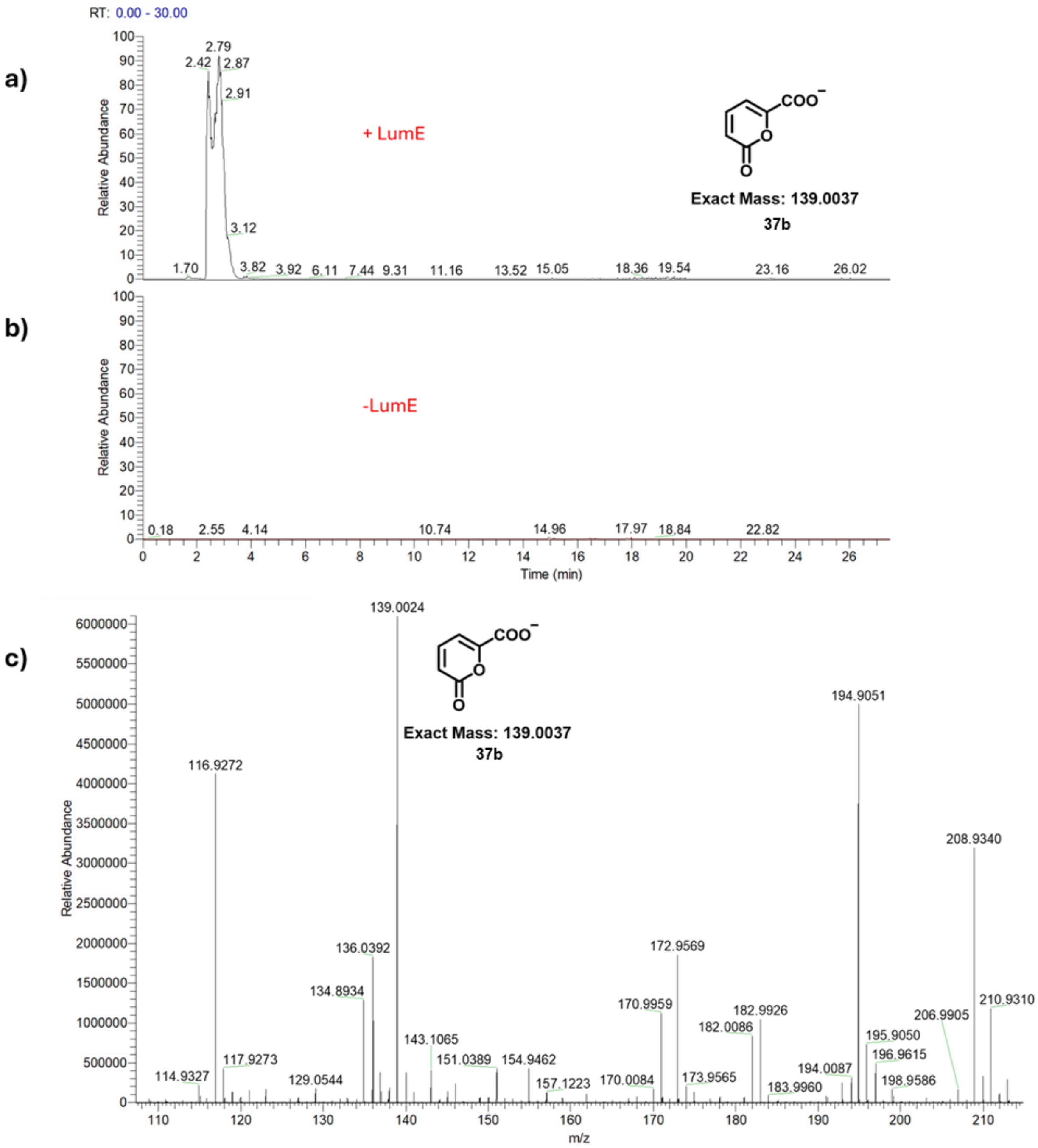

Figure S63. LC-MS data for the LumE product using substrate analog **33b**. EIC of LumE product **37b** (*m/z* 139.0037 Da) a) in the presence of LumE and; b) in the absence of LumE; c) MS of LumE reaction showing the *m/z* 139.0024 Da peak.

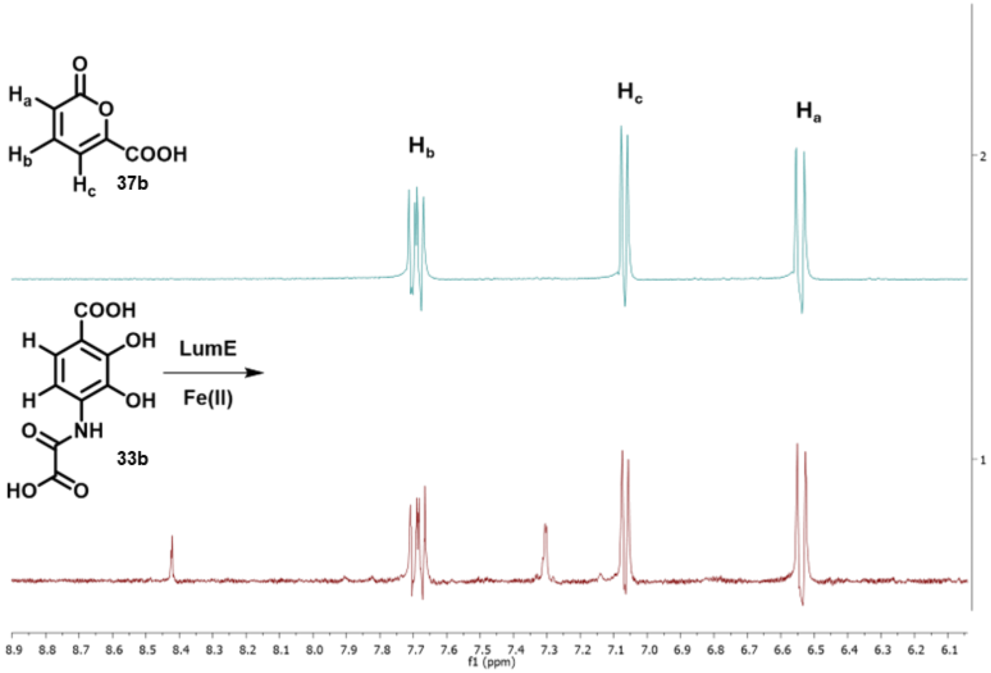

Figure S64. Comparison of ^{1}H NMRs of LumE product from reaction with **33b** and synthetic standard (**37b**) thus confirming formation and structure of pyrone product (**37b**).

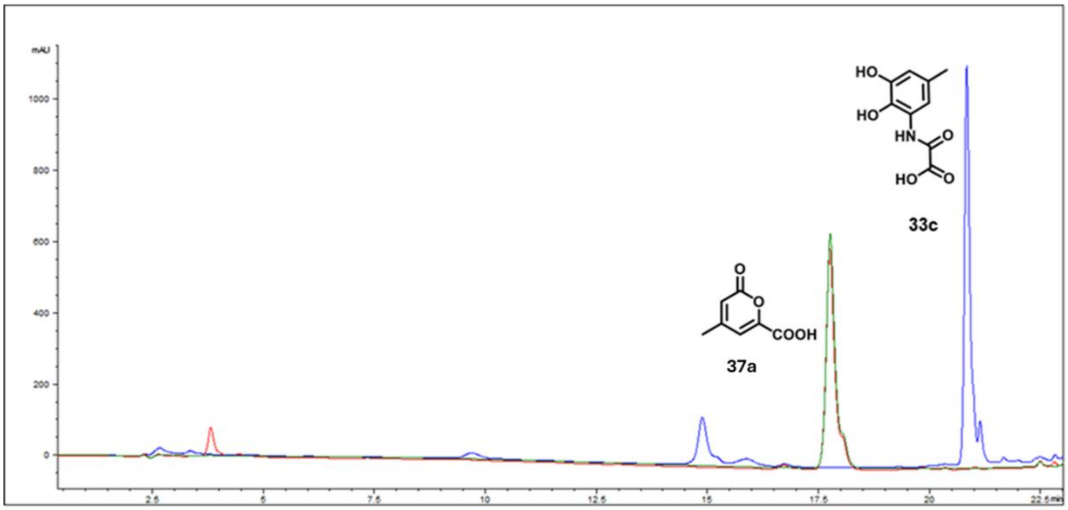

Figure S65. Chromatogram of LumE activity with the substrate analog **33c** forms stoichiometric amounts of lactone **37a** and is the major product in the LumE reaction. Red trace represents the enzymatic reaction. Blue trace is the no enzyme control. Green trace represents the synthetic standard of **37a**.

Synthesis of (Z)-3-methyl-5-oxohex-2-enedioic acid (40) and (Z)-4-methyl-2-oxohex-3-enedioic acid (41)

100 mM of 4-methyl-2-oxo-2H-pyran-6-carboxylic acid (**37a**) was hydrolyzed with 0.5 N NaOH for 15 min. This was diluted 20 times into 100 mM KPi, pH 7.5 (final concentration: 5mM). This mixture was analyzed by ^{1}H NMR.

^{1}H NMR (400 MHz, H_2O+D_2O_salt) δ 6.52 (s, 1H), 6.29 (s, 1H), 3.50 (s, 4H), 3.14 (s, 5H), 2.12 (s, 4H), 2.00 (s, 3H).

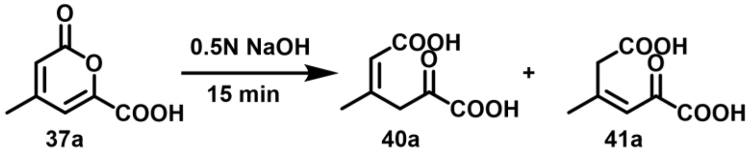

Figure S66. Synthetic scheme for (Z)-3-methyl-5-oxohex-2-enedioic acid (**40a**) and (Z)-4-methyl-2-oxohex-3-enedioic acid (**41a**).

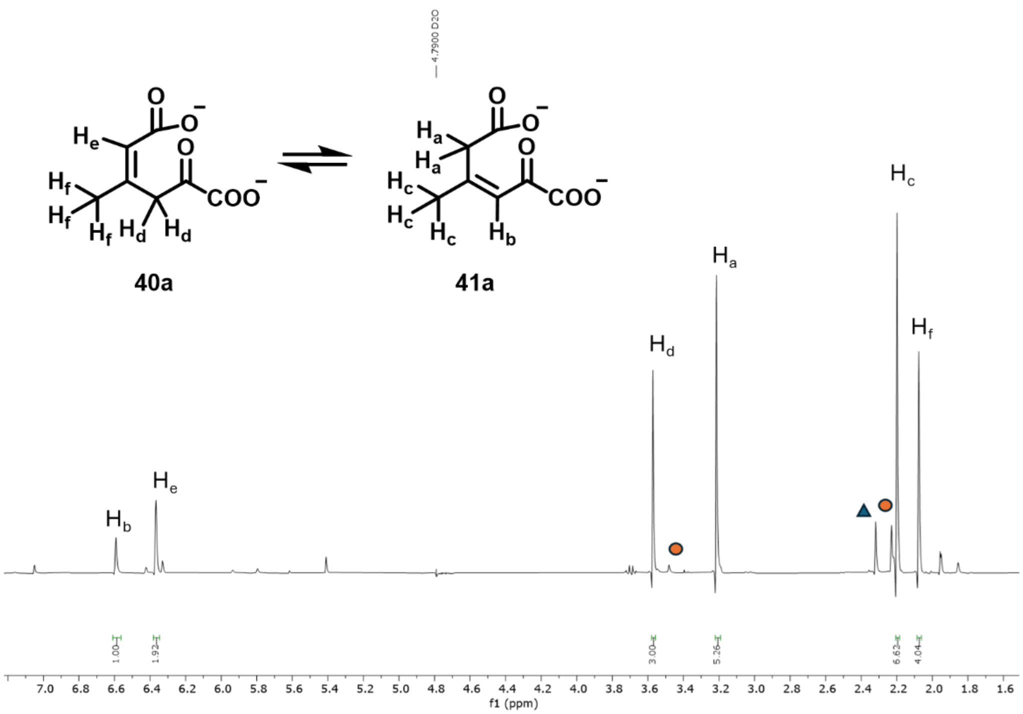

Figure S67. ^{1}H NMR from NaOH hydrolysis of LumW substrate (**37a**) shows the formation of 2-keto isomers of the hydrolyzed product**.** During alkaline hydrolysis, low amounts (<5%) of pyruvate (blue triangle) and acetoacetate (red circles) were also formed.

Characterization of LumW

Overexpression and Purification of LumW

The gene cloned in pTHT (a derivative of the pET28b vector with a TEV protease cleavage site after the N-terminal His-tag) was synthesized by GenScript. This was transformed into *E.coli* C43(DE3) chemical competent cells. Colonies obtained were grown in a 100 mL culture of LB with kanamycin (40 µg/mL) for 12 h at 37 °C (220 rpm). This culture was used to inoculate 1.5 L of LB media at 37 °C. When OD_{600} of 0.6 was reached, 0.5 mM of IPTG was added and the cells were grown for 18 h at 15 °C (120 rpm). This was followed by centrifugation to harvest the cells for 15 min at 5000 rpm and the cells were stored in liquid nitrogen. Typical yields were 10 g of cells (wet weight) from 3 L of cell culture.

For purification, harvested cells were thawed and resuspended in 65 mL phosphate lysis buffer (100 mM KPi, 150 mM NaCl, pH 7.5). Lysozyme (30 mg) and benzonase nuclease (2 µL) were added to this and the suspension was stirred in an ice bath for 30 min. Cells were lysed by sonication, and the mixture was subjected to centrifugation at 15,000 rpm for 40 min to remove the cell debris. The supernatant was filtered using 0.22 µm filters and loaded onto a Ni-NTA His-trap column pre-equilibrated in lysis buffer. The column was then washed with 100 mL of wash buffer (100 mM KPi, 20 mM imidazole, 150 mM NaCl, pH 7.5) and 50 mL of the same buffer with increased imidazole concentration (70 mM). To elute protein, elution buffer (100 mM KPi, 250 mM imidazole, 150 mM NaCl, pH 7.5) was run through the column and fractions of the elute were collected. The fractions were tested with Bradford reagent and the ones containing the protein were pooled and concentrated using 15 mL 10 kDa filters. The buffer of the concentrated protein was exchanged using Cytiva PD-10 desalting columns to 100 mM KPi, 30% glycerol, pH 7.5. The desalted enzyme was pipetted to make aliquots and flash-frozen with liquid nitrogen and stored at -80 °C. Protein concentration was determined using the absorbance at 280 nm (A_{280}) and the extinction coefficient was calculated by the ProtParam tool of the ExPASy proteomics server (ε_{280}=22460 M^{-1} cm^{-1}).

Assay conditions

0.5 mM of substrate was incubated with 100 µM enzyme, 200 µM Co(II) overnight at 37 °C in 0.5 M CHES buffer, pH 8.6. These were passed through 10 kDa PES filters to quench the enzymatic reaction and then analyzed by HPLC.

HPLC conditions

A. Water

B. 100 mM Potassium phosphate buffer, pH 6.6

C. Methanol

HPLC method

(Flow rate: 1 mL/min)

0 min – 100% B, 5 min – 10%A 90%B, 12 min – 48% A 40% B 12% C, 14 min – 50% A 30% B 20% C, 18 min - 30% A 10% B 60%C, 20 min – 100% B, 25 min – 100% B.

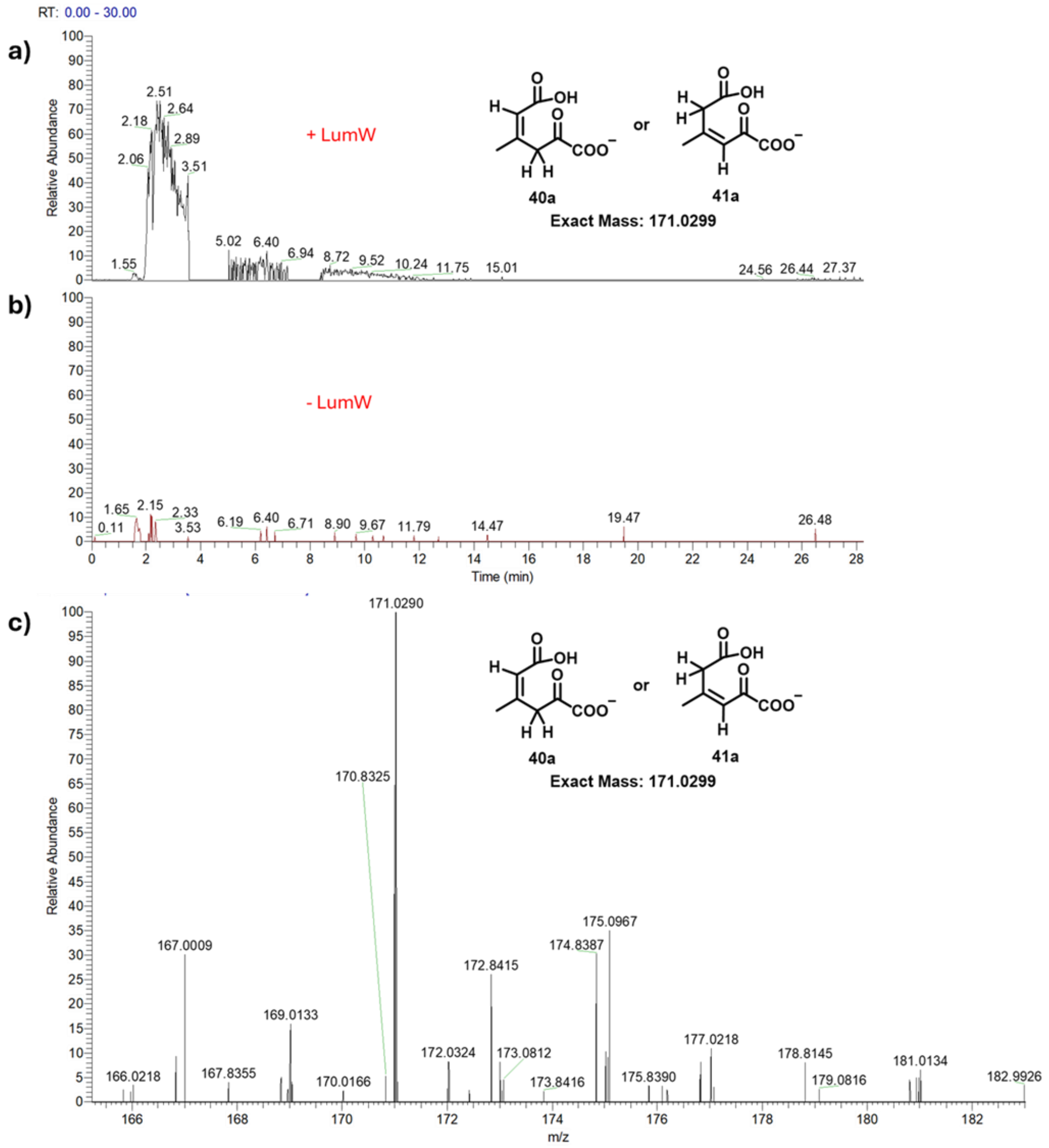

Figure S68. LC-MS data for the LumW native reaction. EIC of LumW product, **40a** or **41a** (*m/z* 171.0299 Da) a) in the presence of LumW and; b) in the absence of LumW; c) MS spectrum of LumW reaction showing the *m/z* 171.0290 Da peak. The existence of the product as a mixture of **40a** and **41a** leads to broadening of the EIC peak on LC-MS.

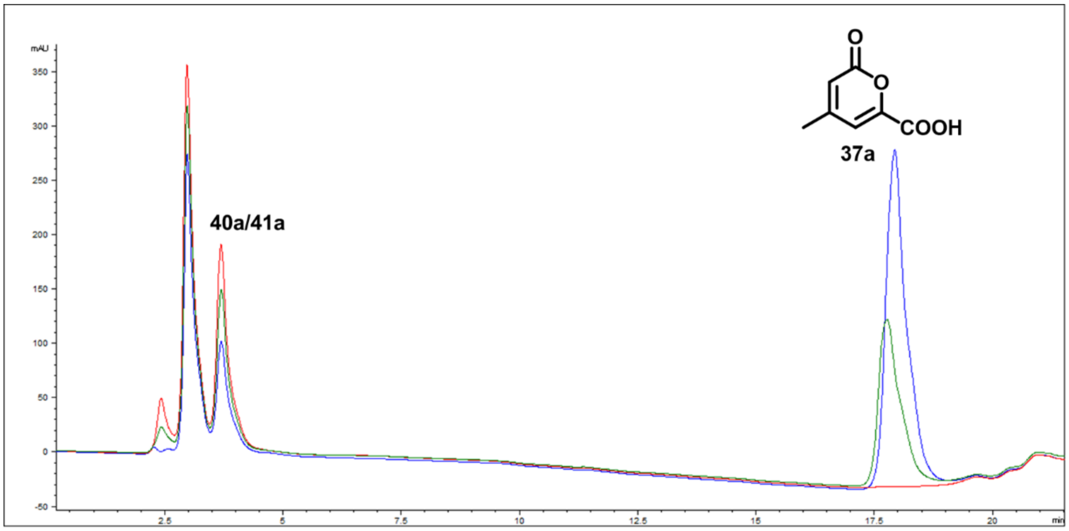

Figure S69. The products from LumW-catalyzed reaction (**40a** and **41a**) co-elutes with products from NaOH hydrolysis of **37a**. Blue trace is LumW enzymatic reaction, red trace is hydrolysis of the lactone from NaOH, green trace is co-elution of the LumW hydrolysis and the synthetic hydrolysis of **37a**.

Characterization of LumX and LumY

Overexpression and Purification of LumX and LumY

The gene cloned in pTHT (a derivative of the pET28b vector with a TEV protease cleavage site after the N-terminal His-tag) was synthesized by GenScript. This was transformed into *E.coli* BL21(DE3) competent cells. Colonies obtained were grown in a 100 mL culture of LB with kanamycin (40 µg/mL) for 12 h at 37 °C (220 rpm). This culture was used to inoculate 1.5 L of LB media at 37 °C. When OD_{600} of 0.6 was reached, 0.5 mM of IPTG was added and the cells were grown for 18 h at 15 °C (120 rpm). This was followed by centrifugation to harvest the cells for 15 min at 5000 rpm and the cells were stored in liquid nitrogen. Typical yields were 10 g of cells (wet weight) from 3 L of cell culture.

For purification, harvested cells were thawed and resuspended in 65 mL phosphate lysis buffer (100 mM KPi, 150 mM NaCl, pH 7.5). Lysozyme (30 mg) and benzonase nuclease (2 µL) were added to this and the suspension was stirred in an ice bath for 30 min. Cells were lysed by sonication and the mixture subjected to centrifugation at 15,000 rpm for 40 min to remove the cell debris. The supernatant was filtered using 0.22 µm filters and loaded onto a Ni-NTA His-trap column pre-equilibrated in lysis buffer. The column was then washed with 100 mL of wash buffer (100 mM KPi, 20 mM imidazole, 150 mM NaCl, pH 7.5) and 50 mL of the same buffer with increased imidazole concentration (70 mM). To elute protein, elution buffer (100 mM KPi, 250 mM imidazole, 150 mM NaCl, pH 7.5) was run through the column and fractions of the elute were collected. The fractions were tested with Bradford reagent and the ones containing the protein were pooled and concentrated using 15 mL 10 kDa filters. The buffer of the concentrated protein was exchanged using Cytiva PD-10 desalting columns to 100 mM KPi, 30% glycerol, pH 7.5. The desalted enzyme was pipetted to make aliquots and flash-frozen with liquid nitrogen and stored at

-80 °C. Protein concentration was determined using the absorbance at 280 nm (A_{280}) and the extinction coefficient calculated by the ProtParam tool of the ExPASy proteomics server (For LumY, ε_{280}= 9970 M^{-1} cm^{-1} and for LumX, ε_{280}= 7115 M^{-1} cm^{-1}).

Assay conditions for NMR experiments

37a was hydrolyzed with 0.5 N NaOH for 15 min. This was diluted 20 times into 100 mM KPi, pH 7.5 (final concentration: 5mM). 25 µM of LumY and 25 µM of Co^{2+} were added to this solution and NMR was done.

For LumY-LumX coupled reaction, 25 µM of LumX was added to the above mixture with other conditions remaining the same.

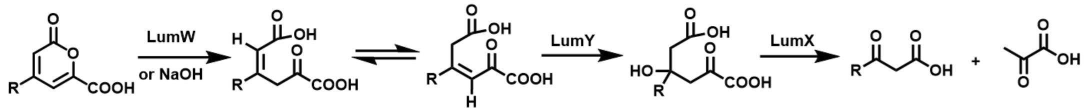

a: R = Me	37a	40a	41a	42a	8	9
b: R = H	37b	40b	41b	42b	8b	

Figure S70. The final steps of lumichrome catabolism with native substrate (**37a**) and substrate analog (**37b**).

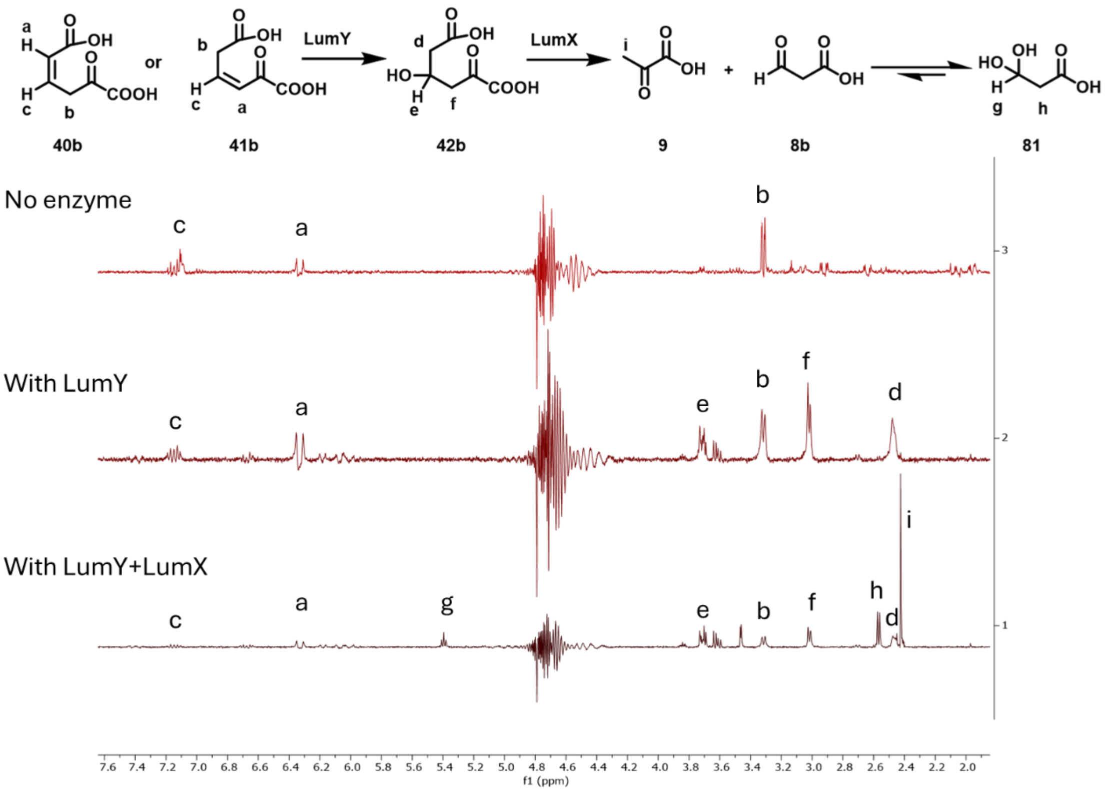

Figure S71. ^{1}H NMR for LumY and LumX reactions with a substrate analog **40b/41b**. a) No enzyme control; b) LumY reaction with 70% substrate consumption. Signals d, e, and f represent the hydrated product; c) Coupling LumY with LumX forms pyruvate (**9**), and 3-oxopropanoate **(8b)**, which exists in its acetal form (signals g and h) **(81)**. The lack of the methyl group in the LumY product (**42b)** retards the retro aldol reaction, making it easier to characterize the product of LumY. The noise around 4.4-4.8 ppm is because of water suppression method employed in these samples.

Culture metabolite analysis for lumichrome catabolic intermediates

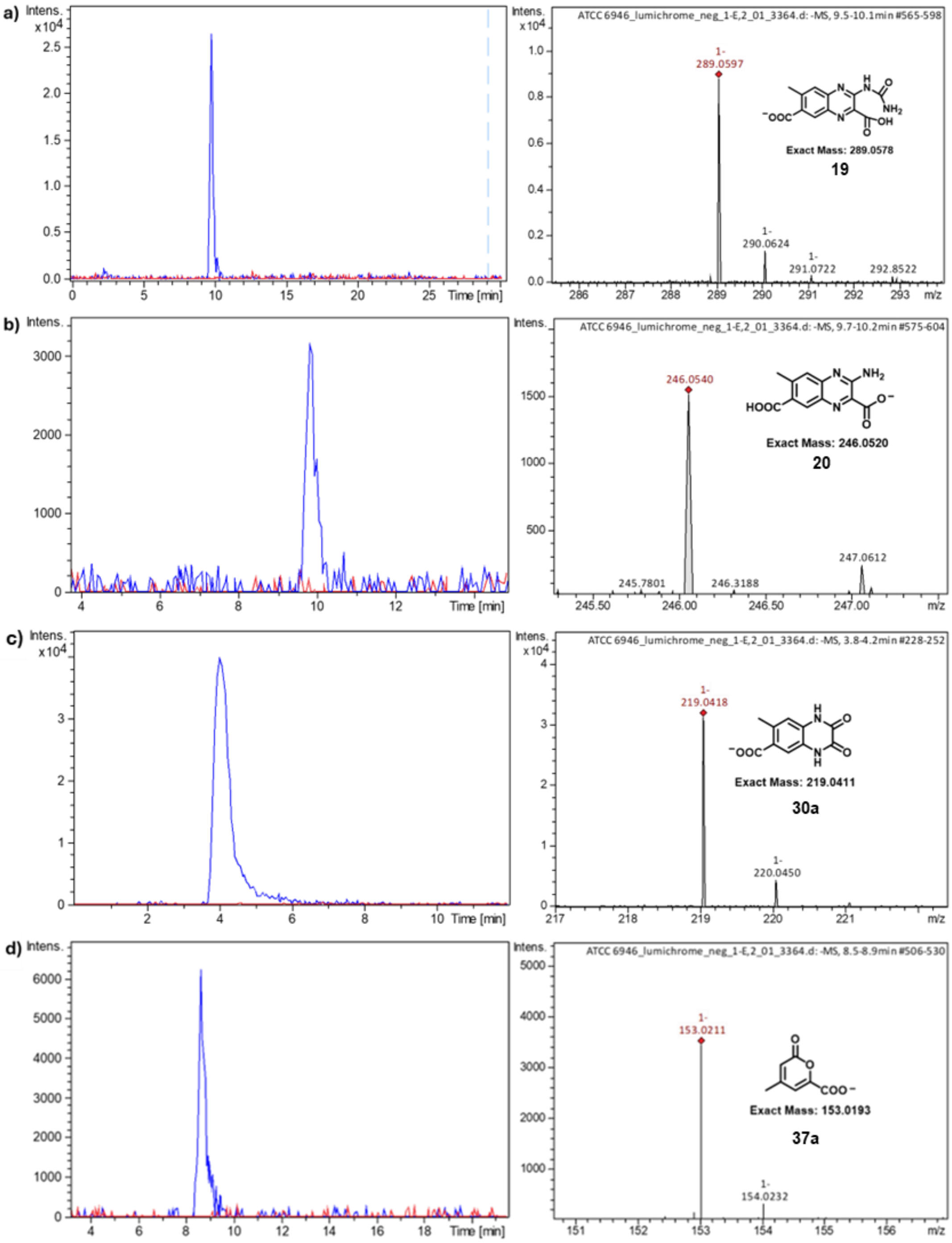

Figure S72. EICs for catabolic pathway intermediates found in metabolomic analysis of the *Nocardioides simplex* ATCC 6946 culture. Blue trace represents the culture containing the catabolic strain while red trace is the control with no catabolic strain. a) EIC of **19** (LumH product); b) EIC of **20** (LumI product); c) EIC of **30a**, (LumJ product); d) EIC of **37a**, (LumE product).

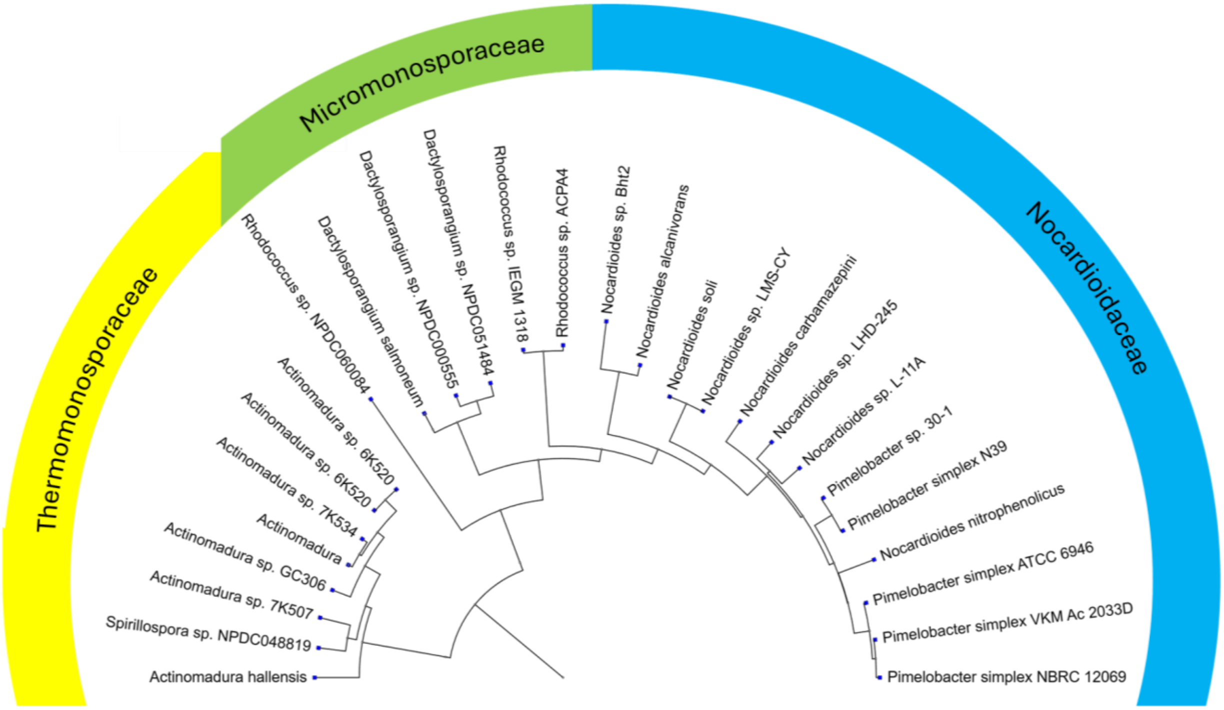

Figure S73. Phylogenetic tree of the lumichrome catabolic pathway, assuming LumU, LumK and LumL, LumJ, LumA, LumB, LumC, and LumD, and LumE are the essential enzymes.

References

(1) Puvvala, S.; Jadhav, V. D.; Narkhede, U. C.; Karun, M. A.; Reddy, C. V. R. First Total Synthesis of a Cytotoxic Derivative of the Natural Product Aaptamine. *Synthesis* **2017**, *49* (12), 2768-2774.

(2) Michael K. Ameriks, G. C., Chaofeng Huang, Brian Ngo LAFORTEZA, Suchitra Ravula, Emma Helen SOUTHGATE, Wei Zhang. Monoacylglycerol lipase modulators. WO2020065613A1, **2019**.

(3) Chen, Y.; Liu, J.; Liu, X.; Jiang, Z.; Wang, S.; Zhang, Y.; Yu, M. Metal-free, late-stage nitration strategy to construct 6-(ortho-nitroaryl) amino purine derivatives enabled by t-BuONO. *Tetrahedron Letters* **2024**, *137*, 154959.

(4) Matsumoto, T.; Harima, S.; Weng, J.-K.; Nihei, K.-i. Systematic approach to the chemical synthesis of arabidopyrones, the unique α-pyrones of Arabidopsis metabolites. *Synthetic Communications* **2020**, *50* (19), 2981-2987.

(5) Higginbotham, L.; Lapworth, A. CLIV.—γ-Oxalyl derivatives of ββ-and αβ-dimethylacrylic acids. *Journal of the Chemical Society, Transactions* **1923**, *123*, 1325-1332.
